# Supplementary material for: Insight into the maintenance of odontogenic potential in mouse dental mesenchymal cells based on transcriptomic analysis
Source: PeerJ. 2016 Feb 22;4:e1684. doi: 10.7717/peerj.1684 (PMC4768683; doi:10.7717/peerj.1684)
Supplement: Data S1 [file peerj-04-1684-s001.doc]

| ensg | mean_P0 | err_P0 | mean_P1 | err_P1 |
| --- | --- | --- | --- | --- |
| ENSMUSG00000000058 | 0 | 0 | 3.432175 | 0.063525 |
| ENSMUSG00000000078 | 0 | 0 | -1.54716 | 0.170826 |
| ENSMUSG00000000120 | 0 | 0 | -8.20465 | 0.322995 |
| ENSMUSG00000000126 | 0 | 0 | 1.906071 | 0.169874 |
| ENSMUSG00000000142 | 0 | 0 | -5.35479 | 0.325357 |
| ENSMUSG00000000223 | 0 | 0 | -2.42581 | 0.155058 |
| ENSMUSG00000000275 | 0 | 0 | 1.531646 | 0.098032 |
| ENSMUSG00000000359 | 0 | 0 | 2.482843 | 0.069784 |
| ENSMUSG00000000409 | 0 | 0 | -1.70553 | 0.196357 |
| ENSMUSG00000000693 | 0 | 0 | 1.707823 | 0.17487 |
| ENSMUSG00000000739 | 0 | 0 | -6.06928 | 0.036165 |
| ENSMUSG00000000753 | 0 | 0 | 1.633231 | 0.21699 |
| ENSMUSG00000000861 | 0 | 0 | -1.60266 | 0.272986 |
| ENSMUSG00000001020 | 0 | 0 | 5.015745 | 0.108353 |
| ENSMUSG00000001025 | 0 | 0 | 3.802798 | 0.090178 |
| ENSMUSG00000001029 | 0 | 0 | -5.47043 | 0.60241 |
| ENSMUSG00000001053 | 0 | 0 | -4.04523 | 0.026869 |
| ENSMUSG00000001123 | 0 | 0 | 2.560854 | 0.329075 |
| ENSMUSG00000001131 | 0 | 0 | 3.857535 | 0.370499 |
| ENSMUSG00000001227 | 0 | 0 | -3.62011 | 0.199793 |
| ENSMUSG00000001240 | 0 | 0 | -4.1594 | 0.150776 |
| ENSMUSG00000001333 | 0 | 0 | 3.217219 | 0.185332 |
| ENSMUSG00000001348 | 0 | 0 | -4.07195 | 0.32915 |
| ENSMUSG00000001349 | 0 | 0 | 11.03416 | 0.26809 |
| ENSMUSG00000001473 | 0 | 0 | 2.692159 | 0.129232 |
| ENSMUSG00000001493 | 0 | 0 | -1.94015 | 0.214603 |
| ENSMUSG00000001506 | 0 | 0 | 1.634423 | 0.077363 |
| ENSMUSG00000001507 | 0 | 0 | 2.752695 | 0.145146 |
| ENSMUSG00000001525 | 0 | 0 | -1.85021 | 0.168492 |
| ENSMUSG00000001555 | 0 | 0 | 1.65625 | 0.030254 |
| ENSMUSG00000001604 | 0 | 0 | 1.91032 | 0.64551 |
| ENSMUSG00000001665 | 0 | 0 | 1.625968 | 0.22738 |
| ENSMUSG00000001751 | 0 | 0 | 2.10885 | 0.101566 |
| ENSMUSG00000001802 | 0 | 0 | -1.87869 | 0.19121 |
| ENSMUSG00000001946 | 0 | 0 | -7.13672 | 0.62269 |
| ENSMUSG00000002020 | 0 | 0 | 6.325241 | 0.070681 |
| ENSMUSG00000002058 | 0 | 0 | -1.86201 | 0.121146 |
| ENSMUSG00000002083 | 0 | 0 | 1.882055 | 0.186481 |
| ENSMUSG00000002105 | 0 | 0 | 1.78555 | 0.041111 |
| ENSMUSG00000002250 | 0 | 0 | 1.566192 | 0.058746 |
| ENSMUSG00000002257 | 0 | 0 | 2.121326 | 0.135812 |
| ENSMUSG00000002289 | 0 | 0 | -1.99672 | 0.250672 |
| ENSMUSG00000002578 | 0 | 0 | -3.04431 | 0.154132 |
| ENSMUSG00000002580 | 0 | 0 | 1.510107 | 0.13124 |
| ENSMUSG00000002799 | 0 | 0 | -5.73762 | 0.670015 |
| ENSMUSG00000002897 | 0 | 0 | 1.576168 | 0.19371 |
| ENSMUSG00000002980 | 0 | 0 | -1.9359 | 0.202347 |
| ENSMUSG00000002985 | 0 | 0 | -2.10709 | 0.255261 |
| ENSMUSG00000003038 | 0 | 0 | -1.6089 | 0.006938 |
| ENSMUSG00000003070 | 0 | 0 | -1.77651 | 0.147017 |
| ENSMUSG00000003575 | 0 | 0 | -1.63865 | 0.200843 |
| ENSMUSG00000003746 | 0 | 0 | 2.205238 | 0.112583 |
| ENSMUSG00000003824 | 0 | 0 | -1.93606 | 0.127703 |
| ENSMUSG00000003849 | 0 | 0 | 2.077201 | 0.157521 |
| ENSMUSG00000003873 | 0 | 0 | 2.095375 | 0.144501 |
| ENSMUSG00000003934 | 0 | 0 | -6.83793 | 0.518515 |
| ENSMUSG00000004085 | 0 | 0 | 2.42303 | 0.155531 |
| ENSMUSG00000004098 | 0 | 0 | 5.034002 | 0.15727 |
| ENSMUSG00000004328 | 0 | 0 | -4.45859 | 0.242424 |
| ENSMUSG00000004415 | 0 | 0 | -7.23093 | 0.317718 |
| ENSMUSG00000004446 | 0 | 0 | 1.703522 | 0.158896 |
| ENSMUSG00000004558 | 0 | 0 | -2.43068 | 0.401599 |
| ENSMUSG00000004633 | 0 | 0 | -3.21259 | 0.055091 |
| ENSMUSG00000004665 | 0 | 0 | 1.569921 | 0.016134 |
| ENSMUSG00000004951 | 0 | 0 | 3.795192 | 0.494835 |
| ENSMUSG00000004994 | 0 | 0 | 1.736736 | 0.140231 |
| ENSMUSG00000005043 | 0 | 0 | 1.610944 | 0.279342 |
| ENSMUSG00000005087 | 0 | 0 | 2.191787 | 0.208235 |
| ENSMUSG00000005124 | 0 | 0 | 2.906669 | 0.032096 |
| ENSMUSG00000005220 | 0 | 0 | -3.19548 | 0.037976 |
| ENSMUSG00000005225 | 0 | 0 | -1.56986 | 0.073332 |
| ENSMUSG00000005237 | 0 | 0 | -4.43102 | 0.416995 |
| ENSMUSG00000005357 | 0 | 0 | 4.231588 | 0.411289 |
| ENSMUSG00000005413 | 0 | 0 | 2.819171 | 0.162154 |
| ENSMUSG00000005483 | 0 | 0 | -1.56824 | 0.176182 |
| ENSMUSG00000005611 | 0 | 0 | 4.481112 | 0.190391 |
| ENSMUSG00000005667 | 0 | 0 | 1.514317 | 0.222649 |
| ENSMUSG00000005672 | 0 | 0 | -3.16628 | 0.487879 |
| ENSMUSG00000005718 | 0 | 0 | -1.89734 | 0.142417 |
| ENSMUSG00000005803 | 0 | 0 | 2.578131 | 0.002259 |
| ENSMUSG00000006205 | 0 | 0 | 3.801108 | 0.07002 |
| ENSMUSG00000006301 | 0 | 0 | 2.302365 | 0.098954 |
| ENSMUSG00000006356 | 0 | 0 | 1.789045 | 0.187008 |
| ENSMUSG00000006369 | 0 | 0 | -1.81924 | 0.189056 |
| ENSMUSG00000006386 | 0 | 0 | -3.54061 | 0.423182 |
| ENSMUSG00000006390 | 0 | 0 | 1.749876 | 0.125216 |
| ENSMUSG00000006445 | 0 | 0 | 1.966298 | 0.331983 |
| ENSMUSG00000006717 | 0 | 0 | 1.828988 | 0.019348 |
| ENSMUSG00000006720 | 0 | 0 | -1.75072 | 0.043646 |
| ENSMUSG00000006731 | 0 | 0 | 1.571041 | 0.203853 |
| ENSMUSG00000006931 | 0 | 0 | 1.627677 | 0.065514 |
| ENSMUSG00000007655 | 0 | 0 | 4.708107 | 0.279782 |
| ENSMUSG00000007805 | 0 | 0 | -2.2184 | 0.080518 |
| ENSMUSG00000007817 | 0 | 0 | -1.92708 | 0.097483 |
| ENSMUSG00000007891 | 0 | 0 | 1.905911 | 0.011702 |
| ENSMUSG00000008136 | 0 | 0 | 3.209589 | 0.092728 |
| ENSMUSG00000008153 | 0 | 0 | -4.70453 | 0.097961 |
| ENSMUSG00000008167 | 0 | 0 | 1.567528 | 0.036452 |
| ENSMUSG00000008575 | 0 | 0 | -2.02305 | 0.116342 |
| ENSMUSG00000008999 | 0 | 0 | -6.91733 | 0.396414 |
| ENSMUSG00000009246 | 0 | 0 | -3.74046 | 0.866238 |
| ENSMUSG00000009376 | 0 | 0 | 2.592398 | 0.610317 |
| ENSMUSG00000009687 | 0 | 0 | 3.701393 | 0.192125 |
| ENSMUSG00000009772 | 0 | 0 | 2.957715 | 0.282993 |
| ENSMUSG00000010154 | 0 | 0 | 4.405602 | 0.103569 |
| ENSMUSG00000010358 | 0 | 0 | 2.72108 | 0.002111 |
| ENSMUSG00000010476 | 0 | 0 | -1.68958 | 0.01234 |
| ENSMUSG00000010529 | 0 | 0 | -2.53927 | 0.344649 |
| ENSMUSG00000011118 | 0 | 0 | -3.31839 | 0.079303 |
| ENSMUSG00000011256 | 0 | 0 | 2.047664 | 0.064672 |
| ENSMUSG00000011463 | 0 | 0 | 1.794107 | 0.432407 |
| ENSMUSG00000012519 | 0 | 0 | 3.288005 | 0.039671 |
| ENSMUSG00000012889 | 0 | 0 | 2.699648 | 0.024689 |
| ENSMUSG00000013584 | 0 | 0 | -4.38453 | 0.055273 |
| ENSMUSG00000014599 | 0 | 0 | 2.676646 | 0.031924 |
| ENSMUSG00000014602 | 0 | 0 | 2.152447 | 0.104524 |
| ENSMUSG00000014850 | 0 | 0 | -1.59628 | 0.176717 |
| ENSMUSG00000015094 | 0 | 0 | 1.805931 | 0.039263 |
| ENSMUSG00000015133 | 0 | 0 | 2.454694 | 0.026632 |
| ENSMUSG00000015143 | 0 | 0 | 1.674756 | 0.059685 |
| ENSMUSG00000015217 | 0 | 0 | -2.24902 | 0.046766 |
| ENSMUSG00000015335 | 0 | 0 | 2.927717 | 0.048883 |
| ENSMUSG00000015396 | 0 | 0 | -5.18869 | 0.030695 |
| ENSMUSG00000015468 | 0 | 0 | -6.45659 | 0.07642 |
| ENSMUSG00000015647 | 0 | 0 | 2.142144 | 0.025971 |
| ENSMUSG00000015652 | 0 | 0 | 3.844061 | 0.145086 |
| ENSMUSG00000015653 | 0 | 0 | 2.28773 | 0.041104 |
| ENSMUSG00000015850 | 0 | 0 | 4.990344 | 0.632596 |
| ENSMUSG00000015932 | 0 | 0 | 1.875443 | 0.13249 |
| ENSMUSG00000015957 | 0 | 0 | -3.80953 | 0.097532 |
| ENSMUSG00000015980 | 0 | 0 | 2.410216 | 0.022558 |
| ENSMUSG00000016028 | 0 | 0 | -3.48301 | 0.257774 |
| ENSMUSG00000016128 | 0 | 0 | 1.600117 | 0.053868 |
| ENSMUSG00000016179 | 0 | 0 | -4.70516 | 0.562207 |
| ENSMUSG00000016382 | 0 | 0 | 1.779209 | 0.36621 |
| ENSMUSG00000016386 | 0 | 0 | -2.39279 | 0.279275 |
| ENSMUSG00000016487 | 0 | 0 | 1.710269 | 0.100523 |
| ENSMUSG00000016559 | 0 | 0 | -1.53262 | 0.119761 |
| ENSMUSG00000016756 | 0 | 0 | -1.55669 | 0.146695 |
| ENSMUSG00000016763 | 0 | 0 | -3.25871 | 0.289366 |
| ENSMUSG00000016918 | 0 | 0 | 2.319849 | 0.035763 |
| ENSMUSG00000017002 | 0 | 0 | 5.244396 | 0.049209 |
| ENSMUSG00000017466 | 0 | 0 | 1.637783 | 0.037372 |
| ENSMUSG00000017491 | 0 | 0 | -2.42852 | 0.228692 |
| ENSMUSG00000017639 | 0 | 0 | -4.95276 | 0.096198 |
| ENSMUSG00000017734 | 0 | 0 | 3.120749 | 0.225305 |
| ENSMUSG00000017737 | 0 | 0 | -5.36635 | 0.156172 |
| ENSMUSG00000017754 | 0 | 0 | 1.500517 | 0.090268 |
| ENSMUSG00000017764 | 0 | 0 | 2.076993 | 0.18092 |
| ENSMUSG00000017774 | 0 | 0 | 2.345482 | 0.165228 |
| ENSMUSG00000017817 | 0 | 0 | 7.440025 | 0.49382 |
| ENSMUSG00000017978 | 0 | 0 | -4.22918 | 0.045335 |
| ENSMUSG00000018012 | 0 | 0 | -1.55826 | 0.053322 |
| ENSMUSG00000018042 | 0 | 0 | 1.602999 | 0.023256 |
| ENSMUSG00000018166 | 0 | 0 | -3.75893 | 0.143334 |
| ENSMUSG00000018171 | 0 | 0 | 2.083078 | 0.038056 |
| ENSMUSG00000018334 | 0 | 0 | -2.76852 | 0.125561 |
| ENSMUSG00000018451 | 0 | 0 | -4.8004 | 0.144131 |
| ENSMUSG00000018476 | 0 | 0 | -1.65107 | 0.172399 |
| ENSMUSG00000018507 | 0 | 0 | 4.674782 | 0.222581 |
| ENSMUSG00000018593 | 0 | 0 | 2.783293 | 0.569285 |
| ENSMUSG00000018819 | 0 | 0 | 2.280307 | 0.086552 |
| ENSMUSG00000018821 | 0 | 0 | 2.365362 | 0.168845 |
| ENSMUSG00000018830 | 0 | 0 | 5.168575 | 0.624319 |
| ENSMUSG00000018899 | 0 | 0 | -2.86876 | 0.121967 |
| ENSMUSG00000018909 | 0 | 0 | -2.71321 | 0.073926 |
| ENSMUSG00000018983 | 0 | 0 | -3.96761 | 0.041485 |
| ENSMUSG00000019027 | 0 | 0 | -2.90655 | 0.373518 |
| ENSMUSG00000019080 | 0 | 0 | 2.595689 | 0.462092 |
| ENSMUSG00000019124 | 0 | 0 | 3.324926 | 0.011694 |
| ENSMUSG00000019194 | 0 | 0 | 3.0714 | 0.046529 |
| ENSMUSG00000019230 | 0 | 0 | 5.116395 | 0.164437 |
| ENSMUSG00000019278 | 0 | 0 | 3.651111 | 0.257945 |
| ENSMUSG00000019647 | 0 | 0 | -2.96598 | 0.109681 |
| ENSMUSG00000019737 | 0 | 0 | -2.81613 | 0.587471 |
| ENSMUSG00000019775 | 0 | 0 | 1.840353 | 0.060409 |
| ENSMUSG00000019796 | 0 | 0 | 1.830917 | 0.214822 |
| ENSMUSG00000019838 | 0 | 0 | -2.24479 | 0.073646 |
| ENSMUSG00000019842 | 0 | 0 | 1.875177 | 0.097237 |
| ENSMUSG00000019850 | 0 | 0 | -2.20308 | 0.333547 |
| ENSMUSG00000019851 | 0 | 0 | 4.661339 | 0.712844 |
| ENSMUSG00000019888 | 0 | 0 | -4.35769 | 0.034058 |
| ENSMUSG00000019906 | 0 | 0 | -1.53425 | 0.124323 |
| ENSMUSG00000019944 | 0 | 0 | -1.50693 | 0.096046 |
| ENSMUSG00000019961 | 0 | 0 | -1.80381 | 0.108968 |
| ENSMUSG00000019990 | 0 | 0 | -2.7725 | 0.616775 |
| ENSMUSG00000019997 | 0 | 0 | 2.849453 | 0.514124 |
| ENSMUSG00000020019 | 0 | 0 | 3.923259 | 0.031927 |
| ENSMUSG00000020023 | 0 | 0 | 2.13254 | 0.254487 |
| ENSMUSG00000020027 | 0 | 0 | -1.90949 | 0.024692 |
| ENSMUSG00000020032 | 0 | 0 | 1.703867 | 0.260121 |
| ENSMUSG00000020056 | 0 | 0 | 1.515611 | 0.059696 |
| ENSMUSG00000020057 | 0 | 0 | 4.477402 | 0.161981 |
| ENSMUSG00000020092 | 0 | 0 | -1.62738 | 0.040841 |
| ENSMUSG00000020100 | 0 | 0 | 1.892133 | 0.084891 |
| ENSMUSG00000020120 | 0 | 0 | -1.50328 | 0.121845 |
| ENSMUSG00000020132 | 0 | 0 | 1.634849 | 0.034485 |
| ENSMUSG00000020140 | 0 | 0 | -4.57645 | 0.077888 |
| ENSMUSG00000020142 | 0 | 0 | 1.641765 | 0.182212 |
| ENSMUSG00000020154 | 0 | 0 | 4.508522 | 0.004308 |
| ENSMUSG00000020183 | 0 | 0 | -1.71099 | 0.158784 |
| ENSMUSG00000020184 | 0 | 0 | 1.713492 | 0.249618 |
| ENSMUSG00000020212 | 0 | 0 | -1.84507 | 0.070676 |
| ENSMUSG00000020218 | 0 | 0 | -7.10348 | 0.052427 |
| ENSMUSG00000020225 | 0 | 0 | 1.652132 | 0.134264 |
| ENSMUSG00000020256 | 0 | 0 | 5.136843 | 0.417911 |
| ENSMUSG00000020263 | 0 | 0 | -1.64737 | 0.124018 |
| ENSMUSG00000020303 | 0 | 0 | 4.961165 | 0.171914 |
| ENSMUSG00000020325 | 0 | 0 | 3.370412 | 0.025313 |
| ENSMUSG00000020326 | 0 | 0 | 4.926163 | 0.133792 |
| ENSMUSG00000020357 | 0 | 0 | -2.58695 | 0.695555 |
| ENSMUSG00000020388 | 0 | 0 | -2.25115 | 0.072055 |
| ENSMUSG00000020411 | 0 | 0 | 6.364184 | 0.35543 |
| ENSMUSG00000020423 | 0 | 0 | -1.61418 | 0.088738 |
| ENSMUSG00000020424 | 0 | 0 | 4.351969 | 0.205046 |
| ENSMUSG00000020439 | 0 | 0 | 1.579483 | 0.05556 |
| ENSMUSG00000020467 | 0 | 0 | 1.917015 | 0.058238 |
| ENSMUSG00000020473 | 0 | 0 | 1.757754 | 0.094356 |
| ENSMUSG00000020547 | 0 | 0 | -1.57849 | 0.102122 |
| ENSMUSG00000020577 | 0 | 0 | -4.06854 | 0.246378 |
| ENSMUSG00000020580 | 0 | 0 | 2.237424 | 0.291492 |
| ENSMUSG00000020601 | 0 | 0 | -1.55487 | 0.031656 |
| ENSMUSG00000020607 | 0 | 0 | -2.20451 | 0.646522 |
| ENSMUSG00000020614 | 0 | 0 | 2.495358 | 0.391249 |
| ENSMUSG00000020623 | 0 | 0 | -2.67361 | 0.366144 |
| ENSMUSG00000020642 | 0 | 0 | -1.91519 | 0.266416 |
| ENSMUSG00000020644 | 0 | 0 | -2.09194 | 0.129843 |
| ENSMUSG00000020653 | 0 | 0 | -3.30398 | 0.044305 |
| ENSMUSG00000020658 | 0 | 0 | -1.54733 | 0.181225 |
| ENSMUSG00000020661 | 0 | 0 | -1.78057 | 0.185053 |
| ENSMUSG00000020689 | 0 | 0 | 1.522475 | 0.152224 |
| ENSMUSG00000020717 | 0 | 0 | -6.92295 | 0.021919 |
| ENSMUSG00000020723 | 0 | 0 | -4.6197 | 0.195581 |
| ENSMUSG00000020736 | 0 | 0 | 1.726985 | 0.147523 |
| ENSMUSG00000020758 | 0 | 0 | 3.529315 | 0.496134 |
| ENSMUSG00000020773 | 0 | 0 | 1.664912 | 0.106404 |
| ENSMUSG00000020806 | 0 | 0 | 2.775709 | 0.000729 |
| ENSMUSG00000020810 | 0 | 0 | -5.67852 | 0.140955 |
| ENSMUSG00000020814 | 0 | 0 | 2.166019 | 0.034403 |
| ENSMUSG00000020828 | 0 | 0 | 1.828883 | 0.108623 |
| ENSMUSG00000020829 | 0 | 0 | 1.535922 | 0.058726 |
| ENSMUSG00000020866 | 0 | 0 | -2.05697 | 0.305254 |
| ENSMUSG00000020882 | 0 | 0 | -1.54166 | 0.372314 |
| ENSMUSG00000020893 | 0 | 0 | -2.01188 | 0.01726 |
| ENSMUSG00000020902 | 0 | 0 | -1.76619 | 0.034576 |
| ENSMUSG00000020937 | 0 | 0 | 2.017842 | 0.199861 |
| ENSMUSG00000021009 | 0 | 0 | 2.464869 | 0.107704 |
| ENSMUSG00000021062 | 0 | 0 | -1.54306 | 0.165309 |
| ENSMUSG00000021069 | 0 | 0 | -1.72961 | 0.340341 |
| ENSMUSG00000021094 | 0 | 0 | 2.098954 | 0.329408 |
| ENSMUSG00000021097 | 0 | 0 | -2.34602 | 0.510494 |
| ENSMUSG00000021108 | 0 | 0 | -2.89582 | 0.171291 |
| ENSMUSG00000021130 | 0 | 0 | -1.67663 | 0.111742 |
| ENSMUSG00000021171 | 0 | 0 | 1.933113 | 0.095021 |
| ENSMUSG00000021182 | 0 | 0 | -1.98699 | 0.331461 |
| ENSMUSG00000021185 | 0 | 0 | 3.634015 | 0.055877 |
| ENSMUSG00000021186 | 0 | 0 | 2.701482 | 0.086255 |
| ENSMUSG00000021196 | 0 | 0 | 2.550198 | 0.103296 |
| ENSMUSG00000021214 | 0 | 0 | 2.999537 | 0.090515 |
| ENSMUSG00000021223 | 0 | 0 | -3.86198 | 0.145931 |
| ENSMUSG00000021256 | 0 | 0 | -4.18658 | 0.043715 |
| ENSMUSG00000021281 | 0 | 0 | 3.143991 | 0.267037 |
| ENSMUSG00000021294 | 0 | 0 | -3.18838 | 0.129262 |
| ENSMUSG00000021367 | 0 | 0 | 2.982006 | 0.745252 |
| ENSMUSG00000021388 | 0 | 0 | 1.92236 | 0.478863 |
| ENSMUSG00000021458 | 0 | 0 | 2.17349 | 0.337584 |
| ENSMUSG00000021466 | 0 | 0 | -4.03537 | 0.121612 |
| ENSMUSG00000021477 | 0 | 0 | 1.536409 | 0.078122 |
| ENSMUSG00000021493 | 0 | 0 | 1.792426 | 0.10175 |
| ENSMUSG00000021575 | 0 | 0 | 1.524471 | 0.311648 |
| ENSMUSG00000021585 | 0 | 0 | 1.950976 | 0.053663 |
| ENSMUSG00000021662 | 0 | 0 | 1.842119 | 0.270005 |
| ENSMUSG00000021668 | 0 | 0 | 2.497063 | 0.281893 |
| ENSMUSG00000021676 | 0 | 0 | -1.50819 | 0.091339 |
| ENSMUSG00000021678 | 0 | 0 | 3.783981 | 0.782922 |
| ENSMUSG00000021702 | 0 | 0 | -2.2614 | 0.362031 |
| ENSMUSG00000021759 | 0 | 0 | 2.734739 | 0.042462 |
| ENSMUSG00000021763 | 0 | 0 | -1.57733 | 0.142228 |
| ENSMUSG00000021767 | 0 | 0 | -1.63733 | 0.193934 |
| ENSMUSG00000021773 | 0 | 0 | 1.738769 | 0.239419 |
| ENSMUSG00000021798 | 0 | 0 | 6.595072 | 0.378407 |
| ENSMUSG00000021814 | 0 | 0 | 1.535403 | 0.060735 |
| ENSMUSG00000021822 | 0 | 0 | 2.887394 | 0.353879 |
| ENSMUSG00000021823 | 0 | 0 | 2.654875 | 0.082212 |
| ENSMUSG00000021866 | 0 | 0 | 2.163519 | 0.324583 |
| ENSMUSG00000021870 | 0 | 0 | 2.194382 | 0.2152 |
| ENSMUSG00000021871 | 0 | 0 | 1.504309 | 0.107153 |
| ENSMUSG00000021876 | 0 | 0 | 3.232924 | 0.085068 |
| ENSMUSG00000021895 | 0 | 0 | -1.98331 | 0.130304 |
| ENSMUSG00000021904 | 0 | 0 | -2.48945 | 0.451486 |
| ENSMUSG00000021943 | 0 | 0 | -3.62789 | 0.00197 |
| ENSMUSG00000021950 | 0 | 0 | 8.177158 | 0.578965 |
| ENSMUSG00000021998 | 0 | 0 | -4.15107 | 0.013697 |
| ENSMUSG00000022010 | 0 | 0 | -1.56416 | 0.06747 |
| ENSMUSG00000022015 | 0 | 0 | -2.14571 | 0.110468 |
| ENSMUSG00000022021 | 0 | 0 | 1.535992 | 0.299292 |
| ENSMUSG00000022032 | 0 | 0 | -4.05689 | 0.595202 |
| ENSMUSG00000022055 | 0 | 0 | -3.2217 | 0.09528 |
| ENSMUSG00000022074 | 0 | 0 | 1.808181 | 0.048725 |
| ENSMUSG00000022090 | 0 | 0 | 2.319218 | 0.073576 |
| ENSMUSG00000022112 | 0 | 0 | 1.847346 | 0.220034 |
| ENSMUSG00000022122 | 0 | 0 | -5.33716 | 0.391935 |
| ENSMUSG00000022146 | 0 | 0 | 6.691897 | 0.019654 |
| ENSMUSG00000022184 | 0 | 0 | 1.754469 | 0.080008 |
| ENSMUSG00000022199 | 0 | 0 | -2.53584 | 0.079524 |
| ENSMUSG00000022203 | 0 | 0 | -1.7183 | 0.075127 |
| ENSMUSG00000022206 | 0 | 0 | 2.024264 | 0.186918 |
| ENSMUSG00000022220 | 0 | 0 | -1.86333 | 0.193584 |
| ENSMUSG00000022231 | 0 | 0 | -2.67416 | 0.41377 |
| ENSMUSG00000022330 | 0 | 0 | -2.72137 | 0.419096 |
| ENSMUSG00000022371 | 0 | 0 | -2.7766 | 0.176026 |
| ENSMUSG00000022376 | 0 | 0 | -6.07486 | 0.066877 |
| ENSMUSG00000022385 | 0 | 0 | 2.06899 | 0.368801 |
| ENSMUSG00000022425 | 0 | 0 | -5.513 | 0.02967 |
| ENSMUSG00000022443 | 0 | 0 | 2.385164 | 0.08044 |
| ENSMUSG00000022449 | 0 | 0 | -2.37421 | 0.041121 |
| ENSMUSG00000022456 | 0 | 0 | -3.07773 | 0.052732 |
| ENSMUSG00000022483 | 0 | 0 | -2.48277 | 0.165169 |
| ENSMUSG00000022489 | 0 | 0 | -4.52002 | 0.057014 |
| ENSMUSG00000022519 | 0 | 0 | -2.14719 | 0.207091 |
| ENSMUSG00000022533 | 0 | 0 | 1.642327 | 0.208167 |
| ENSMUSG00000022544 | 0 | 0 | 1.513312 | 0.162049 |
| ENSMUSG00000022558 | 0 | 0 | 1.785799 | 0.054951 |
| ENSMUSG00000022565 | 0 | 0 | 1.884945 | 0.002159 |
| ENSMUSG00000022575 | 0 | 0 | 2.003491 | 0.116743 |
| ENSMUSG00000022577 | 0 | 0 | -7.28009 | 0.683449 |
| ENSMUSG00000022587 | 0 | 0 | 1.500396 | 0.11605 |
| ENSMUSG00000022594 | 0 | 0 | 4.31024 | 0.308984 |
| ENSMUSG00000022602 | 0 | 0 | -4.5601 | 0.362312 |
| ENSMUSG00000022623 | 0 | 0 | -3.76302 | 0.447394 |
| ENSMUSG00000022629 | 0 | 0 | -1.54416 | 0.009039 |
| ENSMUSG00000022658 | 0 | 0 | -3.20545 | 0.160971 |
| ENSMUSG00000022665 | 0 | 0 | 4.340355 | 0.045408 |
| ENSMUSG00000022673 | 0 | 0 | -1.69065 | 0.025272 |
| ENSMUSG00000022758 | 0 | 0 | 5.475959 | 0.915441 |
| ENSMUSG00000022762 | 0 | 0 | -1.61597 | 0.114392 |
| ENSMUSG00000022773 | 0 | 0 | -2.90509 | 0.480439 |
| ENSMUSG00000022844 | 0 | 0 | 1.708323 | 0.114158 |
| ENSMUSG00000022861 | 0 | 0 | 4.639225 | 0.507934 |
| ENSMUSG00000022865 | 0 | 0 | -2.44069 | 0.281291 |
| ENSMUSG00000022876 | 0 | 0 | -3.50968 | 0.20524 |
| ENSMUSG00000022883 | 0 | 0 | -2.34485 | 0.168664 |
| ENSMUSG00000022885 | 0 | 0 | -1.86371 | 0.179167 |
| ENSMUSG00000022887 | 0 | 0 | 3.822862 | 0.137111 |
| ENSMUSG00000022892 | 0 | 0 | 1.714746 | 0.010133 |
| ENSMUSG00000022894 | 0 | 0 | 3.398833 | 0.223144 |
| ENSMUSG00000022935 | 0 | 0 | -2.16448 | 0.03031 |
| ENSMUSG00000022969 | 0 | 0 | 2.31808 | 0.008351 |
| ENSMUSG00000022999 | 0 | 0 | -1.55486 | 0.04941 |
| ENSMUSG00000023034 | 0 | 0 | -2.76238 | 0.316992 |
| ENSMUSG00000023046 | 0 | 0 | 7.366488 | 0.452325 |
| ENSMUSG00000023048 | 0 | 0 | 1.523324 | 0.023128 |
| ENSMUSG00000023067 | 0 | 0 | 5.918874 | 0.166823 |
| ENSMUSG00000023087 | 0 | 0 | -1.94239 | 0.030605 |
| ENSMUSG00000023088 | 0 | 0 | 1.949663 | 0.106621 |
| ENSMUSG00000023092 | 0 | 0 | 2.995051 | 0.41194 |
| ENSMUSG00000023150 | 0 | 0 | -1.5605 | 0.058073 |
| ENSMUSG00000023224 | 0 | 0 | 1.518663 | 0.020238 |
| ENSMUSG00000023232 | 0 | 0 | -3.20122 | 0.247536 |
| ENSMUSG00000023249 | 0 | 0 | 3.824086 | 0.024602 |
| ENSMUSG00000023805 | 0 | 0 | 2.230846 | 0.084196 |
| ENSMUSG00000023886 | 0 | 0 | -2.66109 | 0.293989 |
| ENSMUSG00000023904 | 0 | 0 | 2.956034 | 0.107419 |
| ENSMUSG00000023905 | 0 | 0 | 2.511862 | 0.416422 |
| ENSMUSG00000023911 | 0 | 0 | -1.91336 | 0.011112 |
| ENSMUSG00000024008 | 0 | 0 | -4.02753 | 0.017205 |
| ENSMUSG00000024014 | 0 | 0 | -2.29304 | 0.307827 |
| ENSMUSG00000024030 | 0 | 0 | -2.67638 | 0.097953 |
| ENSMUSG00000024042 | 0 | 0 | -2.25 | 0.123095 |
| ENSMUSG00000024049 | 0 | 0 | 1.871008 | 0.330401 |
| ENSMUSG00000024063 | 0 | 0 | 1.877681 | 0.061563 |
| ENSMUSG00000024065 | 0 | 0 | 1.515222 | 0.019693 |
| ENSMUSG00000024074 | 0 | 0 | 1.843101 | 0.327759 |
| ENSMUSG00000024087 | 0 | 0 | 2.310258 | 0.15963 |
| ENSMUSG00000024112 | 0 | 0 | -2.21847 | 0.434348 |
| ENSMUSG00000024134 | 0 | 0 | -1.70375 | 0.031619 |
| ENSMUSG00000024140 | 0 | 0 | -1.99204 | 0.106647 |
| ENSMUSG00000024151 | 0 | 0 | -1.52165 | 0.002203 |
| ENSMUSG00000024172 | 0 | 0 | -5.24236 | 0.447304 |
| ENSMUSG00000024190 | 0 | 0 | -1.89352 | 0.355608 |
| ENSMUSG00000024232 | 0 | 0 | -1.80602 | 0.165571 |
| ENSMUSG00000024247 | 0 | 0 | -2.88805 | 0.142792 |
| ENSMUSG00000024302 | 0 | 0 | 2.416461 | 0.268998 |
| ENSMUSG00000024339 | 0 | 0 | 3.493442 | 0.296022 |
| ENSMUSG00000024376 | 0 | 0 | 2.84865 | 0.319893 |
| ENSMUSG00000024427 | 0 | 0 | -1.98215 | 0.288302 |
| ENSMUSG00000024436 | 0 | 0 | 1.631613 | 0.183406 |
| ENSMUSG00000024440 | 0 | 0 | -7.14625 | 1.001641 |
| ENSMUSG00000024451 | 0 | 0 | -4.82541 | 0.076226 |
| ENSMUSG00000024472 | 0 | 0 | -1.56171 | 0.041808 |
| ENSMUSG00000024511 | 0 | 0 | 2.035936 | 0.030942 |
| ENSMUSG00000024521 | 0 | 0 | 4.638378 | 0.188596 |
| ENSMUSG00000024529 | 0 | 0 | 4.113129 | 0.256064 |
| ENSMUSG00000024534 | 0 | 0 | -2.51815 | 0.107897 |
| ENSMUSG00000024590 | 0 | 0 | -2.10716 | 0.00474 |
| ENSMUSG00000024601 | 0 | 0 | -1.65177 | 0.205887 |
| ENSMUSG00000024621 | 0 | 0 | -4.0247 | 0.109489 |
| ENSMUSG00000024659 | 0 | 0 | 3.848446 | 0.116808 |
| ENSMUSG00000024664 | 0 | 0 | 2.449929 | 0.207756 |
| ENSMUSG00000024713 | 0 | 0 | -1.9125 | 0.370473 |
| ENSMUSG00000024725 | 0 | 0 | 2.747194 | 0.01564 |
| ENSMUSG00000024754 | 0 | 0 | -1.50921 | 0.180849 |
| ENSMUSG00000024769 | 0 | 0 | 1.819727 | 0.06823 |
| ENSMUSG00000024778 | 0 | 0 | 3.242593 | 0.136377 |
| ENSMUSG00000024805 | 0 | 0 | 2.963925 | 0.099501 |
| ENSMUSG00000024851 | 0 | 0 | 2.686811 | 0.204002 |
| ENSMUSG00000024854 | 0 | 0 | 1.715715 | 0.107412 |
| ENSMUSG00000024868 | 0 | 0 | -5.29616 | 1.005513 |
| ENSMUSG00000024873 | 0 | 0 | -4.0852 | 0.035511 |
| ENSMUSG00000024875 | 0 | 0 | 1.695566 | 0.027669 |
| ENSMUSG00000024883 | 0 | 0 | 6.756203 | 0.022973 |
| ENSMUSG00000024937 | 0 | 0 | 3.517171 | 0.135011 |
| ENSMUSG00000024942 | 0 | 0 | 1.883973 | 0.137522 |
| ENSMUSG00000024955 | 0 | 0 | 1.580891 | 0.227154 |
| ENSMUSG00000024968 | 0 | 0 | -1.86987 | 0.124789 |
| ENSMUSG00000024975 | 0 | 0 | -1.64583 | 0.424714 |
| ENSMUSG00000024981 | 0 | 0 | 1.550266 | 0.149481 |
| ENSMUSG00000025006 | 0 | 0 | 2.380139 | 0.074255 |
| ENSMUSG00000025026 | 0 | 0 | -2.00414 | 0.277858 |
| ENSMUSG00000025068 | 0 | 0 | 1.596443 | 0.392358 |
| ENSMUSG00000025083 | 0 | 0 | 1.765395 | 0.322988 |
| ENSMUSG00000025085 | 0 | 0 | 2.639516 | 0.129251 |
| ENSMUSG00000025140 | 0 | 0 | 1.903558 | 0.514499 |
| ENSMUSG00000025185 | 0 | 0 | 4.382043 | 0.180181 |
| ENSMUSG00000025212 | 0 | 0 | 1.649187 | 0.025998 |
| ENSMUSG00000025225 | 0 | 0 | 1.619292 | 0.006048 |
| ENSMUSG00000025270 | 0 | 0 | -7.73246 | 0.052625 |
| ENSMUSG00000025278 | 0 | 0 | 2.186912 | 0.174312 |
| ENSMUSG00000025287 | 0 | 0 | 2.174596 | 0.191732 |
| ENSMUSG00000025324 | 0 | 0 | 1.820378 | 0.136402 |
| ENSMUSG00000025348 | 0 | 0 | 3.15741 | 0.643607 |
| ENSMUSG00000025353 | 0 | 0 | 1.531513 | 0.34898 |
| ENSMUSG00000025355 | 0 | 0 | 4.419157 | 0.376691 |
| ENSMUSG00000025375 | 0 | 0 | -5.04486 | 0.3261 |
| ENSMUSG00000034762 | 0 | 0 | -3.64946 | 0.379348 |
| ENSMUSG00000025408 | 0 | 0 | 1.640878 | 0.246549 |
| ENSMUSG00000025422 | 0 | 0 | -7.40801 | 0.013785 |
| ENSMUSG00000025478 | 0 | 0 | -2.75629 | 0.039615 |
| ENSMUSG00000025492 | 0 | 0 | 3.905542 | 0.30319 |
| ENSMUSG00000025504 | 0 | 0 | 1.841718 | 0.064382 |
| ENSMUSG00000025507 | 0 | 0 | 2.475985 | 0.366972 |
| ENSMUSG00000025509 | 0 | 0 | 2.736669 | 0.137873 |
| ENSMUSG00000025521 | 0 | 0 | 2.060744 | 0.00733 |
| ENSMUSG00000025529 | 0 | 0 | -2.01124 | 0.008246 |
| ENSMUSG00000025577 | 0 | 0 | -1.81333 | 0.077076 |
| ENSMUSG00000025582 | 0 | 0 | -5.35872 | 0.000823 |
| ENSMUSG00000025586 | 0 | 0 | 5.743976 | 0.03172 |
| ENSMUSG00000025608 | 0 | 0 | -7.41156 | 0.682602 |
| ENSMUSG00000025658 | 0 | 0 | -1.58248 | 0.045476 |
| ENSMUSG00000025757 | 0 | 0 | 2.076726 | 0.264636 |
| ENSMUSG00000025776 | 0 | 0 | -2.30557 | 0.073838 |
| ENSMUSG00000025780 | 0 | 0 | -2.73203 | 0.286779 |
| ENSMUSG00000025809 | 0 | 0 | 1.585023 | 0.305438 |
| ENSMUSG00000025813 | 0 | 0 | 1.508328 | 0.338241 |
| ENSMUSG00000025854 | 0 | 0 | 1.7439 | 0.207253 |
| ENSMUSG00000025856 | 0 | 0 | 1.892421 | 0.161049 |
| ENSMUSG00000025875 | 0 | 0 | 4.133748 | 0.736138 |
| ENSMUSG00000025887 | 0 | 0 | 2.714255 | 0.06878 |
| ENSMUSG00000025927 | 0 | 0 | -1.85404 | 0.107994 |
| ENSMUSG00000025937 | 0 | 0 | 2.601695 | 0.148505 |
| ENSMUSG00000026034 | 0 | 0 | -2.37287 | 0.134 |
| ENSMUSG00000026069 | 0 | 0 | 6.342569 | 0.61105 |
| ENSMUSG00000026072 | 0 | 0 | 2.220741 | 0.259472 |
| ENSMUSG00000026090 | 0 | 0 | -4.46254 | 0.138856 |
| ENSMUSG00000026109 | 0 | 0 | 1.787401 | 0.227174 |
| ENSMUSG00000026110 | 0 | 0 | -2.14046 | 0.021168 |
| ENSMUSG00000026123 | 0 | 0 | 2.002076 | 0.073874 |
| ENSMUSG00000026142 | 0 | 0 | 2.928304 | 0.238926 |
| ENSMUSG00000026193 | 0 | 0 | 1.646192 | 0.037608 |
| ENSMUSG00000026202 | 0 | 0 | 1.826889 | 0.414932 |
| ENSMUSG00000026204 | 0 | 0 | 5.086805 | 0.54039 |
| ENSMUSG00000026288 | 0 | 0 | -1.81627 | 0.127737 |
| ENSMUSG00000026305 | 0 | 0 | 2.238147 | 0.160849 |
| ENSMUSG00000026308 | 0 | 0 | 5.212173 | 0.760754 |
| ENSMUSG00000026315 | 0 | 0 | 1.642818 | 0.144265 |
| ENSMUSG00000026321 | 0 | 0 | 2.670846 | 0.002435 |
| ENSMUSG00000026344 | 0 | 0 | -3.29549 | 0.032904 |
| ENSMUSG00000026360 | 0 | 0 | -3.76896 | 0.097822 |
| ENSMUSG00000026389 | 0 | 0 | 3.35291 | 0.112034 |
| ENSMUSG00000026398 | 0 | 0 | -3.64795 | 0.237063 |
| ENSMUSG00000026399 | 0 | 0 | 1.77375 | 0.175066 |
| ENSMUSG00000026421 | 0 | 0 | 2.312508 | 0.00205 |
| ENSMUSG00000026430 | 0 | 0 | 3.390783 | 0.369968 |
| ENSMUSG00000026456 | 0 | 0 | 2.217867 | 0.176537 |
| ENSMUSG00000026473 | 0 | 0 | -2.83149 | 0.017197 |
| ENSMUSG00000026479 | 0 | 0 | 1.607265 | 0.551708 |
| ENSMUSG00000026494 | 0 | 0 | -1.63715 | 0.337368 |
| ENSMUSG00000026519 | 0 | 0 | 1.925877 | 0.003055 |
| ENSMUSG00000026532 | 0 | 0 | -2.17593 | 0.019747 |
| ENSMUSG00000026535 | 0 | 0 | 1.80028 | 0.167732 |
| ENSMUSG00000026536 | 0 | 0 | 3.16731 | 0.486341 |
| ENSMUSG00000026547 | 0 | 0 | 1.536293 | 0.214019 |
| ENSMUSG00000026556 | 0 | 0 | -1.70643 | 0.130218 |
| ENSMUSG00000026564 | 0 | 0 | 2.061485 | 0.129323 |
| ENSMUSG00000026576 | 0 | 0 | 1.88081 | 0.710874 |
| ENSMUSG00000026587 | 0 | 0 | -4.50634 | 0.011083 |
| ENSMUSG00000026589 | 0 | 0 | 2.975796 | 0.385393 |
| ENSMUSG00000026594 | 0 | 0 | -3.75345 | 0.012051 |
| ENSMUSG00000026604 | 0 | 0 | 1.825903 | 0.045743 |
| ENSMUSG00000026630 | 0 | 0 | -5.53295 | 0.273087 |
| ENSMUSG00000026637 | 0 | 0 | 1.72222 | 0.104331 |
| ENSMUSG00000026638 | 0 | 0 | -4.95049 | 0.054114 |
| ENSMUSG00000026672 | 0 | 0 | 1.815533 | 0.051023 |
| ENSMUSG00000026676 | 0 | 0 | -2.93452 | 0.316025 |
| ENSMUSG00000026678 | 0 | 0 | -5.09188 | 0.296304 |
| ENSMUSG00000026688 | 0 | 0 | 1.838671 | 0.065933 |
| ENSMUSG00000026712 | 0 | 0 | -3.45956 | 0.086336 |
| ENSMUSG00000026725 | 0 | 0 | 2.215725 | 0.397098 |
| ENSMUSG00000026727 | 0 | 0 | 1.73441 | 0.162589 |
| ENSMUSG00000026728 | 0 | 0 | 2.249061 | 0 |
| ENSMUSG00000026748 | 0 | 0 | 1.988918 | 0.007769 |
| ENSMUSG00000026767 | 0 | 0 | 1.585392 | 0.162748 |
| ENSMUSG00000026768 | 0 | 0 | 3.076102 | 0.262263 |
| ENSMUSG00000026786 | 0 | 0 | 2.549999 | 0.340345 |
| ENSMUSG00000026796 | 0 | 0 | 1.683384 | 0.147672 |
| ENSMUSG00000026817 | 0 | 0 | 4.106885 | 0.165403 |
| ENSMUSG00000026824 | 0 | 0 | -4.78823 | 0.063846 |
| ENSMUSG00000026825 | 0 | 0 | -1.76155 | 0.284747 |
| ENSMUSG00000026826 | 0 | 0 | -2.62497 | 0.197831 |
| ENSMUSG00000026828 | 0 | 0 | -3.17153 | 0.203578 |
| ENSMUSG00000026837 | 0 | 0 | 1.548301 | 0 |
| ENSMUSG00000026840 | 0 | 0 | -3.70967 | 0.168169 |
| ENSMUSG00000026873 | 0 | 0 | 1.676999 | 0.031667 |
| ENSMUSG00000026885 | 0 | 0 | 2.975128 | 0.133221 |
| ENSMUSG00000026890 | 0 | 0 | -1.76698 | 0.038583 |
| ENSMUSG00000026904 | 0 | 0 | 4.633773 | 0.080097 |
| ENSMUSG00000026921 | 0 | 0 | -2.78459 | 0.052285 |
| ENSMUSG00000026932 | 0 | 0 | 2.772645 | 0.15845 |
| ENSMUSG00000026956 | 0 | 0 | 2.914919 | 0.029359 |
| ENSMUSG00000026980 | 0 | 0 | 1.784221 | 0.188563 |
| ENSMUSG00000027004 | 0 | 0 | -5.18493 | 0.157942 |
| ENSMUSG00000027087 | 0 | 0 | 1.710447 | 0.13261 |
| ENSMUSG00000027111 | 0 | 0 | -2.53772 | 0.432426 |
| ENSMUSG00000027134 | 0 | 0 | 1.792155 | 0.041005 |
| ENSMUSG00000027171 | 0 | 0 | 4.69152 | 0.512576 |
| ENSMUSG00000027188 | 0 | 0 | 3.292557 | 0.021011 |
| ENSMUSG00000027200 | 0 | 0 | -2.51121 | 0.195706 |
| ENSMUSG00000027204 | 0 | 0 | 1.824692 | 0.053188 |
| ENSMUSG00000027208 | 0 | 0 | 2.620117 | 0.07079 |
| ENSMUSG00000027217 | 0 | 0 | -2.69817 | 0.219676 |
| ENSMUSG00000027221 | 0 | 0 | -2.46764 | 0.049291 |
| ENSMUSG00000027230 | 0 | 0 | 2.393126 | 0.199032 |
| ENSMUSG00000027239 | 0 | 0 | -2.32773 | 0.00605 |
| ENSMUSG00000027253 | 0 | 0 | -1.51742 | 0.036497 |
| ENSMUSG00000027254 | 0 | 0 | 2.426874 | 0.066915 |
| ENSMUSG00000027313 | 0 | 0 | 5.693465 | 0.323532 |
| ENSMUSG00000027316 | 0 | 0 | -2.71646 | 0.561442 |
| ENSMUSG00000027329 | 0 | 0 | -1.7926 | 0.069352 |
| ENSMUSG00000027335 | 0 | 0 | 7.01698 | 0.030002 |
| ENSMUSG00000027338 | 0 | 0 | -5.37181 | 0.584816 |
| ENSMUSG00000027339 | 0 | 0 | -1.64937 | 0.203819 |
| ENSMUSG00000027342 | 0 | 0 | -1.56112 | 0.022433 |
| ENSMUSG00000027358 | 0 | 0 | -1.95605 | 0.052223 |
| ENSMUSG00000027368 | 0 | 0 | -6.08885 | 0.860846 |
| ENSMUSG00000027416 | 0 | 0 | -1.52213 | 0.432875 |
| ENSMUSG00000027435 | 0 | 0 | -5.32603 | 0.047643 |
| ENSMUSG00000027459 | 0 | 0 | 1.65333 | 0.179167 |
| ENSMUSG00000027478 | 0 | 0 | -1.52652 | 0.040431 |
| ENSMUSG00000027500 | 0 | 0 | 2.990203 | 0.02464 |
| ENSMUSG00000027524 | 0 | 0 | -5.74125 | 0.583866 |
| ENSMUSG00000027544 | 0 | 0 | -3.47707 | 0.299178 |
| ENSMUSG00000027560 | 0 | 0 | 1.708766 | 0.17408 |
| ENSMUSG00000027605 | 0 | 0 | 1.672767 | 0.364218 |
| ENSMUSG00000027620 | 0 | 0 | -1.55764 | 0.087237 |
| ENSMUSG00000027656 | 0 | 0 | 7.85449 | 0.048211 |
| ENSMUSG00000027663 | 0 | 0 | 3.04497 | 0.19349 |
| ENSMUSG00000027698 | 0 | 0 | 2.323558 | 0.164779 |
| ENSMUSG00000027716 | 0 | 0 | -3.89353 | 0.227111 |
| ENSMUSG00000027748 | 0 | 0 | -2.75517 | 0.372143 |
| ENSMUSG00000027763 | 0 | 0 | 1.653127 | 0.146609 |
| ENSMUSG00000027765 | 0 | 0 | -2.44081 | 0.194844 |
| ENSMUSG00000027777 | 0 | 0 | 1.743894 | 0.258316 |
| ENSMUSG00000027796 | 0 | 0 | -1.87163 | 0.05367 |
| ENSMUSG00000027800 | 0 | 0 | 3.789952 | 0.900438 |
| ENSMUSG00000027820 | 0 | 0 | -2.38438 | 0.220131 |
| ENSMUSG00000027827 | 0 | 0 | 2.064095 | 0.60246 |
| ENSMUSG00000027829 | 0 | 0 | -1.85986 | 0.048652 |
| ENSMUSG00000027832 | 0 | 0 | 3.493541 | 0.055724 |
| ENSMUSG00000027858 | 0 | 0 | 3.074053 | 0.081379 |
| ENSMUSG00000027859 | 0 | 0 | 1.96327 | 0.146666 |
| ENSMUSG00000027861 | 0 | 0 | 2.457566 | 0.577543 |
| ENSMUSG00000027907 | 0 | 0 | 1.82416 | 0.0627 |
| ENSMUSG00000027954 | 0 | 0 | -2.20904 | 0.249476 |
| ENSMUSG00000027955 | 0 | 0 | 2.664596 | 0.679411 |
| ENSMUSG00000027956 | 0 | 0 | 2.93154 | 0.184946 |
| ENSMUSG00000027985 | 0 | 0 | -3.15045 | 0.137383 |
| ENSMUSG00000027996 | 0 | 0 | -1.972 | 0.228924 |
| ENSMUSG00000028007 | 0 | 0 | 1.852014 | 0.07268 |
| ENSMUSG00000028019 | 0 | 0 | 1.66426 | 0.002442 |
| ENSMUSG00000028023 | 0 | 0 | -2.69274 | 0.555883 |
| ENSMUSG00000028024 | 0 | 0 | -3.69845 | 0.566385 |
| ENSMUSG00000028031 | 0 | 0 | -1.78386 | 0.19118 |
| ENSMUSG00000028042 | 0 | 0 | 5.920468 | 0.012798 |
| ENSMUSG00000028076 | 0 | 0 | -1.79639 | 0.467457 |
| ENSMUSG00000028108 | 0 | 0 | 8.171155 | 1.042631 |
| ENSMUSG00000028128 | 0 | 0 | 3.776503 | 0.430851 |
| ENSMUSG00000028159 | 0 | 0 | 1.621618 | 0.096217 |
| ENSMUSG00000028194 | 0 | 0 | 1.527756 | 0.259026 |
| ENSMUSG00000028197 | 0 | 0 | -2.2428 | 0.095895 |
| ENSMUSG00000028211 | 0 | 0 | 2.424599 | 0.119284 |
| ENSMUSG00000028214 | 0 | 0 | -3.08574 | 0.201466 |
| ENSMUSG00000028251 | 0 | 0 | 2.191779 | 0.064051 |
| ENSMUSG00000028259 | 0 | 0 | 4.425432 | 0.02122 |
| ENSMUSG00000028270 | 0 | 0 | 3.493246 | 0.401438 |
| ENSMUSG00000028273 | 0 | 0 | 4.375732 | 0.07379 |
| ENSMUSG00000028278 | 0 | 0 | -1.84471 | 0.08604 |
| ENSMUSG00000028339 | 0 | 0 | -4.22838 | 0.39677 |
| ENSMUSG00000028341 | 0 | 0 | -2.29779 | 0.158271 |
| ENSMUSG00000028358 | 0 | 0 | -1.64134 | 0.227624 |
| ENSMUSG00000028360 | 0 | 0 | -3.33407 | 0.143503 |
| ENSMUSG00000028369 | 0 | 0 | 2.512553 | 0.06754 |
| ENSMUSG00000028370 | 0 | 0 | 4.206778 | 0.059262 |
| ENSMUSG00000028464 | 0 | 0 | 3.161131 | 0.00652 |
| ENSMUSG00000028466 | 0 | 0 | 1.655143 | 0.045787 |
| ENSMUSG00000028480 | 0 | 0 | 1.990824 | 0.128688 |
| ENSMUSG00000028497 | 0 | 0 | 2.142617 | 0.268329 |
| ENSMUSG00000028524 | 0 | 0 | -2.36218 | 0.1973 |
| ENSMUSG00000028539 | 0 | 0 | 4.30974 | 0.619791 |
| ENSMUSG00000028545 | 0 | 0 | -1.81861 | 0.286066 |
| ENSMUSG00000028565 | 0 | 0 | -2.05003 | 0.13654 |
| ENSMUSG00000028581 | 0 | 0 | -3.70471 | 0.033149 |
| ENSMUSG00000028583 | 0 | 0 | 2.086062 | 0.020071 |
| ENSMUSG00000028600 | 0 | 0 | 1.605128 | 0.035362 |
| ENSMUSG00000028613 | 0 | 0 | 1.757315 | 0.290967 |
| ENSMUSG00000028626 | 0 | 0 | -5.58334 | 0.213016 |
| ENSMUSG00000028640 | 0 | 0 | -4.04123 | 0.520664 |
| ENSMUSG00000028654 | 0 | 0 | -2.20642 | 0.413694 |
| ENSMUSG00000028656 | 0 | 0 | 1.556364 | 0.05239 |
| ENSMUSG00000028671 | 0 | 0 | 2.463274 | 0.081512 |
| ENSMUSG00000028693 | 0 | 0 | -2.07908 | 0.078078 |
| ENSMUSG00000028744 | 0 | 0 | 3.299872 | 0.08688 |
| ENSMUSG00000028766 | 0 | 0 | -4.56534 | 0.406206 |
| ENSMUSG00000028773 | 0 | 0 | 3.882483 | 0.285394 |
| ENSMUSG00000028776 | 0 | 0 | 5.568429 | 0.535167 |
| ENSMUSG00000028780 | 0 | 0 | 4.989661 | 0.088754 |
| ENSMUSG00000028782 | 0 | 0 | -3.84472 | 0.310199 |
| ENSMUSG00000028820 | 0 | 0 | -1.63584 | 0.059847 |
| ENSMUSG00000028832 | 0 | 0 | -1.64312 | 0.065685 |
| ENSMUSG00000028871 | 0 | 0 | -4.40207 | 0.150774 |
| ENSMUSG00000028893 | 0 | 0 | 2.158128 | 0.223418 |
| ENSMUSG00000028906 | 0 | 0 | -1.91355 | 0.119483 |
| ENSMUSG00000028909 | 0 | 0 | -2.56186 | 0.075479 |
| ENSMUSG00000028919 | 0 | 0 | 3.203343 | 0.028653 |
| ENSMUSG00000028931 | 0 | 0 | -2.06016 | 0.114581 |
| ENSMUSG00000028970 | 0 | 0 | 5.138536 | 0.660973 |
| ENSMUSG00000028978 | 0 | 0 | -6.5185 | 0.367804 |
| ENSMUSG00000029007 | 0 | 0 | 1.744256 | 0.227371 |
| ENSMUSG00000029061 | 0 | 0 | 4.845518 | 0.177149 |
| ENSMUSG00000029070 | 0 | 0 | 1.7283 | 0.168919 |
| ENSMUSG00000029086 | 0 | 0 | -5.31466 | 0.204059 |
| ENSMUSG00000029090 | 0 | 0 | -1.7459 | 0.065962 |
| ENSMUSG00000029094 | 0 | 0 | 1.71087 | 0.098806 |
| ENSMUSG00000029119 | 0 | 0 | 2.33624 | 0.455932 |
| ENSMUSG00000029161 | 0 | 0 | 4.329831 | 0.005425 |
| ENSMUSG00000029175 | 0 | 0 | 1.616161 | 0.096308 |
| ENSMUSG00000029178 | 0 | 0 | 1.962208 | 0.047365 |
| ENSMUSG00000029185 | 0 | 0 | 1.537068 | 0.179323 |
| ENSMUSG00000029228 | 0 | 0 | -2.36389 | 0.348128 |
| ENSMUSG00000029231 | 0 | 0 | -1.70116 | 0.365568 |
| ENSMUSG00000029283 | 0 | 0 | -1.67005 | 0.15964 |
| ENSMUSG00000029287 | 0 | 0 | -1.73755 | 0.008861 |
| ENSMUSG00000029304 | 0 | 0 | 2.594368 | 0.286571 |
| ENSMUSG00000029306 | 0 | 0 | -2.51527 | 0.128071 |
| ENSMUSG00000029309 | 0 | 0 | -2.07555 | 0.21133 |
| ENSMUSG00000029313 | 0 | 0 | 1.637504 | 0.177621 |
| ENSMUSG00000029333 | 0 | 0 | -2.481 | 0.175506 |
| ENSMUSG00000029335 | 0 | 0 | -2.5173 | 0.258673 |
| ENSMUSG00000029348 | 0 | 0 | -1.61035 | 0.127549 |
| ENSMUSG00000029364 | 0 | 0 | 1.999877 | 0.121112 |
| ENSMUSG00000029377 | 0 | 0 | 5.243001 | 0.329411 |
| ENSMUSG00000029381 | 0 | 0 | 3.192456 | 0.034169 |
| ENSMUSG00000029438 | 0 | 0 | -1.76565 | 0.032936 |
| ENSMUSG00000029446 | 0 | 0 | 1.760177 | 0.052199 |
| ENSMUSG00000029470 | 0 | 0 | 1.92574 | 0.044045 |
| ENSMUSG00000029482 | 0 | 0 | 1.876757 | 0.005075 |
| ENSMUSG00000029484 | 0 | 0 | 4.160083 | 0.438123 |
| ENSMUSG00000029510 | 0 | 0 | -2.01746 | 0.186851 |
| ENSMUSG00000029552 | 0 | 0 | 1.71033 | 0.073037 |
| ENSMUSG00000029557 | 0 | 0 | 1.617346 | 0.274809 |
| ENSMUSG00000029576 | 0 | 0 | 2.167163 | 0.020395 |
| ENSMUSG00000029591 | 0 | 0 | -1.61238 | 0.288415 |
| ENSMUSG00000029603 | 0 | 0 | -5.04846 | 0.133536 |
| ENSMUSG00000029634 | 0 | 0 | 1.997886 | 0.165055 |
| ENSMUSG00000029651 | 0 | 0 | 1.532633 | 0.111068 |
| ENSMUSG00000029659 | 0 | 0 | 2.274815 | 0.137856 |
| ENSMUSG00000029671 | 0 | 0 | 1.723126 | 0.193053 |
| ENSMUSG00000029673 | 0 | 0 | -2.48177 | 0.369295 |
| ENSMUSG00000029675 | 0 | 0 | -1.60312 | 0.140564 |
| ENSMUSG00000029708 | 0 | 0 | -1.57946 | 0.131565 |
| ENSMUSG00000029718 | 0 | 0 | 1.930104 | 0.239353 |
| ENSMUSG00000029752 | 0 | 0 | 2.31682 | 0.172188 |
| ENSMUSG00000029761 | 0 | 0 | 2.653276 | 0.131917 |
| ENSMUSG00000029762 | 0 | 0 | 2.528342 | 0.123949 |
| ENSMUSG00000029777 | 0 | 0 | 1.591416 | 0.149337 |
| ENSMUSG00000029816 | 0 | 0 | 6.128555 | 0.020068 |
| ENSMUSG00000029826 | 0 | 0 | 2.227797 | 0.199016 |
| ENSMUSG00000029860 | 0 | 0 | 2.014944 | 0.121543 |
| ENSMUSG00000030075 | 0 | 0 | -2.08939 | 0.128386 |
| ENSMUSG00000030077 | 0 | 0 | 1.637509 | 0.145173 |
| ENSMUSG00000030089 | 0 | 0 | 3.244435 | 0.12281 |
| ENSMUSG00000030091 | 0 | 0 | -3.21782 | 0.037979 |
| ENSMUSG00000030092 | 0 | 0 | -2.42345 | 0.05905 |
| ENSMUSG00000030116 | 0 | 0 | 6.092272 | 0.616111 |
| ENSMUSG00000030123 | 0 | 0 | -3.08021 | 0.135758 |
| ENSMUSG00000030203 | 0 | 0 | -1.89122 | 0.100635 |
| ENSMUSG00000030220 | 0 | 0 | 2.100118 | 0.176617 |
| ENSMUSG00000030226 | 0 | 0 | -3.13122 | 0.083668 |
| ENSMUSG00000030247 | 0 | 0 | -6.84788 | 0.347305 |
| ENSMUSG00000030249 | 0 | 0 | -4.54524 | 0.208908 |
| ENSMUSG00000030268 | 0 | 0 | 3.119912 | 0.174695 |
| ENSMUSG00000030279 | 0 | 0 | -1.65766 | 0.14193 |
| ENSMUSG00000030281 | 0 | 0 | 5.458524 | 0.061946 |
| ENSMUSG00000030284 | 0 | 0 | 3.447431 | 0.286354 |
| ENSMUSG00000030306 | 0 | 0 | 2.218363 | 0.777663 |
| ENSMUSG00000030315 | 0 | 0 | -1.53419 | 0.01282 |
| ENSMUSG00000030347 | 0 | 0 | 1.571554 | 0.112309 |
| ENSMUSG00000030353 | 0 | 0 | 2.175392 | 0.395944 |
| ENSMUSG00000030376 | 0 | 0 | -4.2825 | 0.273495 |
| ENSMUSG00000030409 | 0 | 0 | 2.718001 | 0.061618 |
| ENSMUSG00000030468 | 0 | 0 | 7.8673 | 2.211661 |
| ENSMUSG00000030493 | 0 | 0 | 1.653898 | 0.012388 |
| ENSMUSG00000030522 | 0 | 0 | 2.508934 | 0.467982 |
| ENSMUSG00000030538 | 0 | 0 | 1.754655 | 0.005482 |
| ENSMUSG00000030592 | 0 | 0 | -1.84223 | 0.407052 |
| ENSMUSG00000030598 | 0 | 0 | 1.629442 | 0.083468 |
| ENSMUSG00000030600 | 0 | 0 | -2.1918 | 0.018298 |
| ENSMUSG00000030605 | 0 | 0 | 1.837181 | 0.166055 |
| ENSMUSG00000030607 | 0 | 0 | 4.783674 | 0.488369 |
| ENSMUSG00000030609 | 0 | 0 | 1.941094 | 0.265373 |
| ENSMUSG00000030615 | 0 | 0 | 1.532775 | 0.175647 |
| ENSMUSG00000030616 | 0 | 0 | 2.301492 | 0.045945 |
| ENSMUSG00000030621 | 0 | 0 | -2.34635 | 0.280753 |
| ENSMUSG00000030623 | 0 | 0 | 2.859956 | 0.266697 |
| ENSMUSG00000030630 | 0 | 0 | 2.713395 | 0.205963 |
| ENSMUSG00000030638 | 0 | 0 | -1.78602 | 0.15714 |
| ENSMUSG00000030641 | 0 | 0 | 1.661041 | 0.176705 |
| ENSMUSG00000030653 | 0 | 0 | -5.60371 | 0.04859 |
| ENSMUSG00000030659 | 0 | 0 | 1.663722 | 0.058528 |
| ENSMUSG00000030669 | 0 | 0 | -4.84462 | 0.331893 |
| ENSMUSG00000030681 | 0 | 0 | 3.304583 | 0.045024 |
| ENSMUSG00000030707 | 0 | 0 | -2.93111 | 0.000864 |
| ENSMUSG00000030717 | 0 | 0 | 7.090241 | 0.839429 |
| ENSMUSG00000030725 | 0 | 0 | -1.55229 | 0.222466 |
| ENSMUSG00000030748 | 0 | 0 | 1.545848 | 0.067982 |
| ENSMUSG00000030772 | 0 | 0 | 2.406086 | 0.119347 |
| ENSMUSG00000030782 | 0 | 0 | 3.524841 | 0.01115 |
| ENSMUSG00000030790 | 0 | 0 | 5.702463 | 0.04593 |
| ENSMUSG00000030811 | 0 | 0 | -1.76315 | 0.005177 |
| ENSMUSG00000030862 | 0 | 0 | 3.172005 | 0.099479 |
| ENSMUSG00000030894 | 0 | 0 | 2.632856 | 0.017976 |
| ENSMUSG00000030905 | 0 | 0 | -3.00025 | 0.478603 |
| ENSMUSG00000030946 | 0 | 0 | -1.71657 | 0.279507 |
| ENSMUSG00000031007 | 0 | 0 | 1.539805 | 0.069644 |
| ENSMUSG00000031026 | 0 | 0 | 4.729391 | 0.04889 |
| ENSMUSG00000031028 | 0 | 0 | -2.7673 | 0.606948 |
| ENSMUSG00000031066 | 0 | 0 | -1.71809 | 0.359718 |
| ENSMUSG00000031070 | 0 | 0 | 4.998566 | 0.132593 |
| ENSMUSG00000031099 | 0 | 0 | -1.83208 | 0.156705 |
| ENSMUSG00000031176 | 0 | 0 | 2.543727 | 0.050093 |
| ENSMUSG00000031207 | 0 | 0 | 2.157449 | 0.294874 |
| ENSMUSG00000031209 | 0 | 0 | -1.83789 | 0.106002 |
| ENSMUSG00000031253 | 0 | 0 | 3.712535 | 0.201621 |
| ENSMUSG00000031273 | 0 | 0 | 1.777125 | 0.117637 |
| ENSMUSG00000031274 | 0 | 0 | 1.941466 | 0.053494 |
| ENSMUSG00000031289 | 0 | 0 | 3.513468 | 0.43356 |
| ENSMUSG00000031297 | 0 | 0 | 4.097236 | 0.107089 |
| ENSMUSG00000031302 | 0 | 0 | -4.83352 | 0.061721 |
| ENSMUSG00000031328 | 0 | 0 | 1.881711 | 0 |
| ENSMUSG00000031342 | 0 | 0 | 1.836853 | 0.029385 |
| ENSMUSG00000031349 | 0 | 0 | 1.617459 | 0.108736 |
| ENSMUSG00000031351 | 0 | 0 | -1.53892 | 0.014628 |
| ENSMUSG00000031373 | 0 | 0 | 2.899829 | 0.014903 |
| ENSMUSG00000031375 | 0 | 0 | 1.850257 | 0.074126 |
| ENSMUSG00000031387 | 0 | 0 | 1.834935 | 0.208063 |
| ENSMUSG00000031451 | 0 | 0 | 1.648928 | 0.042928 |
| ENSMUSG00000031461 | 0 | 0 | 1.768376 | 0.602521 |
| ENSMUSG00000031465 | 0 | 0 | 4.007282 | 0.61546 |
| ENSMUSG00000031480 | 0 | 0 | -2.57926 | 0.214421 |
| ENSMUSG00000031490 | 0 | 0 | 1.894082 | 0.002589 |
| ENSMUSG00000031517 | 0 | 0 | -3.72551 | 0.946682 |
| ENSMUSG00000031519 | 0 | 0 | 1.824065 | 0.018441 |
| ENSMUSG00000031521 | 0 | 0 | 2.210963 | 0.041959 |
| ENSMUSG00000031555 | 0 | 0 | 2.123364 | 0.023699 |
| ENSMUSG00000031558 | 0 | 0 | 1.757185 | 0.182454 |
| ENSMUSG00000031591 | 0 | 0 | 1.999128 | 0.053902 |
| ENSMUSG00000031595 | 0 | 0 | 3.608296 | 0.459753 |
| ENSMUSG00000031616 | 0 | 0 | -3.0843 | 0.227174 |
| ENSMUSG00000031626 | 0 | 0 | 2.882387 | 0.344389 |
| ENSMUSG00000031661 | 0 | 0 | -2.83401 | 0.200928 |
| ENSMUSG00000031714 | 0 | 0 | -2.14893 | 0.194219 |
| ENSMUSG00000031762 | 0 | 0 | 5.536342 | 0.025529 |
| ENSMUSG00000031765 | 0 | 0 | 4.808539 | 0.313834 |
| ENSMUSG00000031767 | 0 | 0 | 1.628658 | 0.241487 |
| ENSMUSG00000031772 | 0 | 0 | -3.55319 | 0.121411 |
| ENSMUSG00000031778 | 0 | 0 | 4.248844 | 0.658293 |
| ENSMUSG00000031790 | 0 | 0 | -3.36434 | 0.074677 |
| ENSMUSG00000031822 | 0 | 0 | -1.8526 | 0.082957 |
| ENSMUSG00000031837 | 0 | 0 | -5.09994 | 0.423683 |
| ENSMUSG00000031871 | 0 | 0 | -11.3789 | 1.251387 |
| ENSMUSG00000031887 | 0 | 0 | 2.191284 | 0.013352 |
| ENSMUSG00000031897 | 0 | 0 | 1.760017 | 0.006051 |
| ENSMUSG00000031906 | 0 | 0 | -4.28765 | 0.044504 |
| ENSMUSG00000031972 | 0 | 0 | 3.047442 | 0.346439 |
| ENSMUSG00000031994 | 0 | 0 | -2.12086 | 0.138478 |
| ENSMUSG00000032006 | 0 | 0 | 2.140773 | 0.131431 |
| ENSMUSG00000032011 | 0 | 0 | 3.148303 | 0.652476 |
| ENSMUSG00000032020 | 0 | 0 | -1.95704 | 0.033922 |
| ENSMUSG00000032024 | 0 | 0 | -1.64713 | 0.289179 |
| ENSMUSG00000032026 | 0 | 0 | 2.728717 | 0.050372 |
| ENSMUSG00000032060 | 0 | 0 | 6.052794 | 0.327775 |
| ENSMUSG00000032085 | 0 | 0 | 6.46712 | 0.433819 |
| ENSMUSG00000032101 | 0 | 0 | -2.06187 | 0.181624 |
| ENSMUSG00000032177 | 0 | 0 | 2.40029 | 0.32284 |
| ENSMUSG00000032179 | 0 | 0 | -2.19319 | 0.360534 |
| ENSMUSG00000032228 | 0 | 0 | -1.75499 | 0.048288 |
| ENSMUSG00000032232 | 0 | 0 | -1.74209 | 0.086973 |
| ENSMUSG00000032243 | 0 | 0 | 1.673545 | 0.297631 |
| ENSMUSG00000032249 | 0 | 0 | -1.7852 | 0.082815 |
| ENSMUSG00000032271 | 0 | 0 | 3.148727 | 0.148583 |
| ENSMUSG00000032280 | 0 | 0 | -1.70944 | 0.079415 |
| ENSMUSG00000032291 | 0 | 0 | 1.963456 | 0.197002 |
| ENSMUSG00000032332 | 0 | 0 | 2.255958 | 0.119905 |
| ENSMUSG00000032366 | 0 | 0 | 3.44069 | 0.025161 |
| ENSMUSG00000032372 | 0 | 0 | 1.643971 | 0.075432 |
| ENSMUSG00000032418 | 0 | 0 | 3.407854 | 0.242466 |
| ENSMUSG00000032452 | 0 | 0 | 2.290367 | 0.230384 |
| ENSMUSG00000032492 | 0 | 0 | -2.3179 | 0.10914 |
| ENSMUSG00000032501 | 0 | 0 | -2.06172 | 0.460324 |
| ENSMUSG00000032502 | 0 | 0 | -2.19229 | 0.01886 |
| ENSMUSG00000032504 | 0 | 0 | 1.510503 | 0.059726 |
| ENSMUSG00000032515 | 0 | 0 | -3.18537 | 0.491188 |
| ENSMUSG00000032554 | 0 | 0 | -2.59499 | 0.090321 |
| ENSMUSG00000032565 | 0 | 0 | 1.908159 | 0.095819 |
| ENSMUSG00000032578 | 0 | 0 | -1.93918 | 0.237553 |
| ENSMUSG00000032609 | 0 | 0 | -1.6514 | 0.229281 |
| ENSMUSG00000032624 | 0 | 0 | -1.97063 | 0.106238 |
| ENSMUSG00000032643 | 0 | 0 | 1.83072 | 0.350038 |
| ENSMUSG00000032649 | 0 | 0 | -3.41772 | 0.367498 |
| ENSMUSG00000032656 | 0 | 0 | 1.789696 | 0.02223 |
| ENSMUSG00000032698 | 0 | 0 | -4.22888 | 0.009205 |
| ENSMUSG00000032715 | 0 | 0 | 8.284304 | 0.329261 |
| ENSMUSG00000032717 | 0 | 0 | -2.84608 | 0.091095 |
| ENSMUSG00000032719 | 0 | 0 | 3.375345 | 0.133784 |
| ENSMUSG00000032754 | 0 | 0 | 1.740299 | 0.101828 |
| ENSMUSG00000032802 | 0 | 0 | 1.772845 | 0.181059 |
| ENSMUSG00000032827 | 0 | 0 | -2.38499 | 0.286059 |
| ENSMUSG00000032849 | 0 | 0 | 2.355252 | 0.113062 |
| ENSMUSG00000032850 | 0 | 0 | -2.70635 | 0.087012 |
| ENSMUSG00000032899 | 0 | 0 | 4.418028 | 0.354761 |
| ENSMUSG00000032911 | 0 | 0 | 1.636547 | 0.310798 |
| ENSMUSG00000032936 | 0 | 0 | -9.77149 | 1.096724 |
| ENSMUSG00000033032 | 0 | 0 | -2.64238 | 0.204098 |
| ENSMUSG00000033033 | 0 | 0 | 2.3468 | 0.118825 |
| ENSMUSG00000033060 | 0 | 0 | 2.167113 | 0.098951 |
| ENSMUSG00000033083 | 0 | 0 | -1.94427 | 0.073489 |
| ENSMUSG00000033149 | 0 | 0 | 2.501378 | 0.001235 |
| ENSMUSG00000033152 | 0 | 0 | -2.44375 | 0.306009 |
| ENSMUSG00000033174 | 0 | 0 | 1.868399 | 0.126752 |
| ENSMUSG00000033207 | 0 | 0 | 4.682586 | 0.288881 |
| ENSMUSG00000033306 | 0 | 0 | 2.78729 | 0.052303 |
| ENSMUSG00000033364 | 0 | 0 | -1.62137 | 0.008905 |
| ENSMUSG00000033386 | 0 | 0 | 3.393103 | 0.014081 |
| ENSMUSG00000033453 | 0 | 0 | -3.06853 | 0.060316 |
| ENSMUSG00000033467 | 0 | 0 | 3.138827 | 0.193882 |
| ENSMUSG00000033491 | 0 | 0 | 1.93292 | 0.257502 |
| ENSMUSG00000033502 | 0 | 0 | -1.5533 | 0.0643 |
| ENSMUSG00000033576 | 0 | 0 | 2.643607 | 0.459862 |
| ENSMUSG00000033707 | 0 | 0 | 2.344616 | 0.088331 |
| ENSMUSG00000033717 | 0 | 0 | 1.549016 | 0.390754 |
| ENSMUSG00000033739 | 0 | 0 | 2.476497 | 0.126873 |
| ENSMUSG00000033809 | 0 | 0 | 1.582135 | 0.085139 |
| ENSMUSG00000033855 | 0 | 0 | 2.107566 | 0.021452 |
| ENSMUSG00000033880 | 0 | 0 | 2.788357 | 0.882563 |
| ENSMUSG00000033965 | 0 | 0 | -1.93968 | 0.088861 |
| ENSMUSG00000033981 | 0 | 0 | -2.84969 | 0.13323 |
| ENSMUSG00000034037 | 0 | 0 | -7.66007 | 0.653275 |
| ENSMUSG00000034040 | 0 | 0 | 2.845526 | 0.046018 |
| ENSMUSG00000034127 | 0 | 0 | -4.61817 | 0.357642 |
| ENSMUSG00000034161 | 0 | 0 | 2.72666 | 0.425307 |
| ENSMUSG00000034164 | 0 | 0 | -3.3146 | 0.577513 |
| ENSMUSG00000034177 | 0 | 0 | 2.970707 | 0.01331 |
| ENSMUSG00000034245 | 0 | 0 | -1.77281 | 0.204949 |
| ENSMUSG00000034255 | 0 | 0 | -2.63243 | 0.381318 |
| ENSMUSG00000034266 | 0 | 0 | -2.57343 | 0.396862 |
| ENSMUSG00000034275 | 0 | 0 | 4.305563 | 0.328282 |
| ENSMUSG00000034324 | 0 | 0 | -7.80462 | 0.269524 |
| ENSMUSG00000034327 | 0 | 0 | 1.562983 | 0.023281 |
| ENSMUSG00000034361 | 0 | 0 | 3.356959 | 0.060576 |
| ENSMUSG00000034394 | 0 | 0 | 5.482459 | 0.002316 |
| ENSMUSG00000034413 | 0 | 0 | -1.53122 | 0.055775 |
| ENSMUSG00000034457 | 0 | 0 | 4.404438 | 0.193587 |
| ENSMUSG00000034591 | 0 | 0 | 3.963411 | 0.032524 |
| ENSMUSG00000034648 | 0 | 0 | -2.70081 | 0.550507 |
| ENSMUSG00000034744 | 0 | 0 | 1.825504 | 0.001893 |
| ENSMUSG00000034771 | 0 | 0 | -3.25356 | 0.473691 |
| ENSMUSG00000034810 | 0 | 0 | 2.446039 | 0.598392 |
| ENSMUSG00000034845 | 0 | 0 | -9.17979 | 0.022386 |
| ENSMUSG00000034853 | 0 | 0 | 1.973782 | 0.044023 |
| ENSMUSG00000034911 | 0 | 0 | -7.6816 | 0.04967 |
| ENSMUSG00000034949 | 0 | 0 | -1.7674 | 0.104498 |
| ENSMUSG00000034957 | 0 | 0 | -2.2628 | 0.372446 |
| ENSMUSG00000034981 | 0 | 0 | 2.120382 | 0.555529 |
| ENSMUSG00000034993 | 0 | 0 | 2.385364 | 0.183404 |
| ENSMUSG00000035105 | 0 | 0 | 2.010356 | 0.339049 |
| ENSMUSG00000035165 | 0 | 0 | -9.11748 | 1.621002 |
| ENSMUSG00000035228 | 0 | 0 | -2.44337 | 0.335775 |
| ENSMUSG00000035279 | 0 | 0 | 3.452071 | 0.239462 |
| ENSMUSG00000035305 | 0 | 0 | -2.52712 | 0.223595 |
| ENSMUSG00000035356 | 0 | 0 | -2.48949 | 0.286285 |
| ENSMUSG00000035373 | 0 | 0 | 4.34718 | 0.282748 |
| ENSMUSG00000035385 | 0 | 0 | 4.420655 | 0.302746 |
| ENSMUSG00000035407 | 0 | 0 | -2.20712 | 0.154667 |
| ENSMUSG00000035441 | 0 | 0 | 2.039315 | 0.202017 |
| ENSMUSG00000035517 | 0 | 0 | 1.64323 | 0.099855 |
| ENSMUSG00000035557 | 0 | 0 | -10.4651 | 2.25733 |
| ENSMUSG00000035559 | 0 | 0 | 2.951032 | 0.181109 |
| ENSMUSG00000035566 | 0 | 0 | -1.67945 | 0.018349 |
| ENSMUSG00000035595 | 0 | 0 | 3.601644 | 0.312793 |
| ENSMUSG00000035778 | 0 | 0 | 1.715978 | 0.22974 |
| ENSMUSG00000035783 | 0 | 0 | 8.150702 | 0.245678 |
| ENSMUSG00000035828 | 0 | 0 | -1.59566 | 0.226896 |
| ENSMUSG00000035835 | 0 | 0 | -3.581 | 0.31208 |
| ENSMUSG00000035873 | 0 | 0 | 2.94253 | 0.092959 |
| ENSMUSG00000035916 | 0 | 0 | 1.954888 | 0.234351 |
| ENSMUSG00000035936 | 0 | 0 | -2.10093 | 0.030036 |
| ENSMUSG00000035964 | 0 | 0 | -7.36313 | 0.151535 |
| ENSMUSG00000036002 | 0 | 0 | 1.558102 | 0.086361 |
| ENSMUSG00000036006 | 0 | 0 | 3.573937 | 0.394259 |
| ENSMUSG00000036019 | 0 | 0 | -2.00982 | 0.1287 |
| ENSMUSG00000036067 | 0 | 0 | 5.63525 | 0.539368 |
| ENSMUSG00000036136 | 0 | 0 | 3.397793 | 0.043585 |
| ENSMUSG00000036169 | 0 | 0 | -5.1094 | 0.215685 |
| ENSMUSG00000036256 | 0 | 0 | 2.550641 | 0.007772 |
| ENSMUSG00000036292 | 0 | 0 | -2.11923 | 0.162035 |
| ENSMUSG00000036298 | 0 | 0 | -1.76525 | 0.267881 |
| ENSMUSG00000036356 | 0 | 0 | -2.33707 | 0.180491 |
| ENSMUSG00000036412 | 0 | 0 | 2.736349 | 0.054273 |
| ENSMUSG00000036452 | 0 | 0 | -1.56099 | 0.084293 |
| ENSMUSG00000036478 | 0 | 0 | -1.7273 | 0.192433 |
| ENSMUSG00000036502 | 0 | 0 | -4.65412 | 0.04948 |
| ENSMUSG00000036528 | 0 | 0 | -2.01954 | 0.162426 |
| ENSMUSG00000036533 | 0 | 0 | 2.281187 | 0.412272 |
| ENSMUSG00000036534 | 0 | 0 | 1.6212 | 0.119086 |
| ENSMUSG00000036564 | 0 | 0 | 2.891418 | 0.07889 |
| ENSMUSG00000036599 | 0 | 0 | 3.470874 | 0.102526 |
| ENSMUSG00000036632 | 0 | 0 | 1.526799 | 0.150679 |
| ENSMUSG00000036745 | 0 | 0 | 2.80482 | 0.342529 |
| ENSMUSG00000036766 | 0 | 0 | 2.28304 | 0.139487 |
| ENSMUSG00000036820 | 0 | 0 | 1.816709 | 0.062094 |
| ENSMUSG00000036875 | 0 | 0 | -1.62875 | 0.180909 |
| ENSMUSG00000036918 | 0 | 0 | 1.844971 | 0.181796 |
| ENSMUSG00000036948 | 0 | 0 | 2.495454 | 0.623367 |
| ENSMUSG00000036966 | 0 | 0 | 1.899338 | 0.059885 |
| ENSMUSG00000037031 | 0 | 0 | -1.84843 | 0.010199 |
| ENSMUSG00000037035 | 0 | 0 | 4.768326 | 0.253953 |
| ENSMUSG00000037071 | 0 | 0 | 1.709997 | 0.179456 |
| ENSMUSG00000037108 | 0 | 0 | -2.70189 | 0.256113 |
| ENSMUSG00000037169 | 0 | 0 | -6.23038 | 1.016577 |
| ENSMUSG00000037190 | 0 | 0 | 1.517481 | 0.003469 |
| ENSMUSG00000037206 | 0 | 0 | -2.74706 | 0.469894 |
| ENSMUSG00000037211 | 0 | 0 | -2.13194 | 0.043464 |
| ENSMUSG00000037239 | 0 | 0 | 1.95713 | 0.160778 |
| ENSMUSG00000037349 | 0 | 0 | 1.942279 | 0.411106 |
| ENSMUSG00000037362 | 0 | 0 | 4.840288 | 0.244465 |
| ENSMUSG00000037370 | 0 | 0 | -5.46466 | 0.143893 |
| ENSMUSG00000037405 | 0 | 0 | -1.67089 | 0.256106 |
| ENSMUSG00000037411 | 0 | 0 | 4.335163 | 0.236521 |
| ENSMUSG00000037465 | 0 | 0 | -1.83307 | 0.099091 |
| ENSMUSG00000037492 | 0 | 0 | -5.82762 | 0.801832 |
| ENSMUSG00000037594 | 0 | 0 | 2.884859 | 0.046106 |
| ENSMUSG00000037625 | 0 | 0 | -5.6842 | 0.053206 |
| ENSMUSG00000037664 | 0 | 0 | -1.85484 | 0.174442 |
| ENSMUSG00000037679 | 0 | 0 | 2.454355 | 0.172842 |
| ENSMUSG00000037754 | 0 | 0 | -2.17846 | 0.189568 |
| ENSMUSG00000037813 | 0 | 0 | -3.1576 | 0.639064 |
| ENSMUSG00000037814 | 0 | 0 | 2.303556 | 0.158924 |
| ENSMUSG00000037820 | 0 | 0 | -1.55534 | 0.061859 |
| ENSMUSG00000037855 | 0 | 0 | 2.59515 | 0.176944 |
| ENSMUSG00000037868 | 0 | 0 | -3.57173 | 0.458938 |
| ENSMUSG00000037892 | 0 | 0 | -1.5202 | 0.071415 |
| ENSMUSG00000037946 | 0 | 0 | 5.042595 | 0.459816 |
| ENSMUSG00000037990 | 0 | 0 | 2.554785 | 0.066049 |
| ENSMUSG00000037995 | 0 | 0 | -2.45134 | 0.295785 |
| ENSMUSG00000038028 | 0 | 0 | 1.774785 | 0.347859 |
| ENSMUSG00000038065 | 0 | 0 | -2.82744 | 0.384827 |
| ENSMUSG00000038068 | 0 | 0 | 1.580973 | 0.174109 |
| ENSMUSG00000038077 | 0 | 0 | -2.46307 | 0.277416 |
| ENSMUSG00000038156 | 0 | 0 | -3.71613 | 0.50732 |
| ENSMUSG00000038172 | 0 | 0 | 1.72997 | 0.082182 |
| ENSMUSG00000038188 | 0 | 0 | -9.22593 | 1.220525 |
| ENSMUSG00000038235 | 0 | 0 | -3.39181 | 0.059293 |
| ENSMUSG00000038244 | 0 | 0 | 2.022789 | 0.203288 |
| ENSMUSG00000038264 | 0 | 0 | -3.81019 | 0.214993 |
| ENSMUSG00000038267 | 0 | 0 | -2.03533 | 0.003062 |
| ENSMUSG00000038280 | 0 | 0 | 2.093608 | 0.006864 |
| ENSMUSG00000038305 | 0 | 0 | -1.61527 | 0.224254 |
| ENSMUSG00000038331 | 0 | 0 | -2.10581 | 0.071214 |
| ENSMUSG00000038375 | 0 | 0 | 1.854932 | 0.077269 |
| ENSMUSG00000038387 | 0 | 0 | 3.335111 | 0.152933 |
| ENSMUSG00000038390 | 0 | 0 | -1.67762 | 0.207767 |
| ENSMUSG00000038400 | 0 | 0 | 2.302799 | 0.038511 |
| ENSMUSG00000038486 | 0 | 0 | -3.10299 | 0.078382 |
| ENSMUSG00000038517 | 0 | 0 | -2.13012 | 0.146351 |
| ENSMUSG00000038518 | 0 | 0 | -2.18663 | 0.234566 |
| ENSMUSG00000038539 | 0 | 0 | 3.097642 | 0.185695 |
| ENSMUSG00000038623 | 0 | 0 | -2.03275 | 0.025404 |
| ENSMUSG00000038642 | 0 | 0 | -2.41756 | 0.070754 |
| ENSMUSG00000038742 | 0 | 0 | 3.462249 | 0.719456 |
| ENSMUSG00000038775 | 0 | 0 | -3.10435 | 0.087339 |
| ENSMUSG00000038776 | 0 | 0 | 6.676522 | 0.093452 |
| ENSMUSG00000038777 | 0 | 0 | -2.87431 | 0.054641 |
| ENSMUSG00000038893 | 0 | 0 | -1.51254 | 0.045062 |
| ENSMUSG00000038910 | 0 | 0 | -1.92317 | 0.000454 |
| ENSMUSG00000038932 | 0 | 0 | 2.725858 | 0.269508 |
| ENSMUSG00000039004 | 0 | 0 | -3.31696 | 0.100743 |
| ENSMUSG00000039005 | 0 | 0 | 3.226899 | 0.311928 |
| ENSMUSG00000039037 | 0 | 0 | -4.99154 | 0.19624 |
| ENSMUSG00000039058 | 0 | 0 | 3.893014 | 0.239627 |
| ENSMUSG00000039103 | 0 | 0 | 3.626055 | 0.200273 |
| ENSMUSG00000039109 | 0 | 0 | -3.69182 | 0.176992 |
| ENSMUSG00000039115 | 0 | 0 | -2.84897 | 0.206927 |
| ENSMUSG00000039126 | 0 | 0 | 3.514548 | 0.073913 |
| ENSMUSG00000039153 | 0 | 0 | -2.06577 | 0.104866 |
| ENSMUSG00000039157 | 0 | 0 | -2.10493 | 0.35964 |
| ENSMUSG00000039167 | 0 | 0 | -1.62831 | 0.166537 |
| ENSMUSG00000039182 | 0 | 0 | 1.988244 | 0.092576 |
| ENSMUSG00000039232 | 0 | 0 | 2.125382 | 0.107097 |
| ENSMUSG00000039252 | 0 | 0 | -2.39165 | 0.558735 |
| ENSMUSG00000039286 | 0 | 0 | 1.580384 | 0.152874 |
| ENSMUSG00000039323 | 0 | 0 | 2.326034 | 0.07004 |
| ENSMUSG00000039328 | 0 | 0 | -2.60726 | 0.168108 |
| ENSMUSG00000039347 | 0 | 0 | 3.349418 | 0.506014 |
| ENSMUSG00000039349 | 0 | 0 | -3.64786 | 0.467217 |
| ENSMUSG00000039361 | 0 | 0 | 1.833801 | 0.126032 |
| ENSMUSG00000039405 | 0 | 0 | 2.53775 | 0.543983 |
| ENSMUSG00000039419 | 0 | 0 | -1.77081 | 0.132862 |
| ENSMUSG00000039450 | 0 | 0 | 5.185714 | 0.487248 |
| ENSMUSG00000039461 | 0 | 0 | 1.533281 | 0.262066 |
| ENSMUSG00000039470 | 0 | 0 | 1.979922 | 0.113764 |
| ENSMUSG00000039474 | 0 | 0 | 2.574101 | 0.249967 |
| ENSMUSG00000039568 | 0 | 0 | -2.26475 | 0.155548 |
| ENSMUSG00000039579 | 0 | 0 | -2.40218 | 0.191285 |
| ENSMUSG00000039601 | 0 | 0 | 2.211626 | 0.212715 |
| ENSMUSG00000039621 | 0 | 0 | -3.20016 | 0.173852 |
| ENSMUSG00000039697 | 0 | 0 | -1.68588 | 0.062305 |
| ENSMUSG00000039706 | 0 | 0 | -1.96741 | 0.03744 |
| ENSMUSG00000039713 | 0 | 0 | -1.68177 | 0.182568 |
| ENSMUSG00000039745 | 0 | 0 | 2.298948 | 0.018739 |
| ENSMUSG00000039813 | 0 | 0 | 6.816509 | 0.055791 |
| ENSMUSG00000039910 | 0 | 0 | 1.572818 | 0.073422 |
| ENSMUSG00000039943 | 0 | 0 | 2.049719 | 0.217885 |
| ENSMUSG00000039976 | 0 | 0 | -2.8519 | 0.064419 |
| ENSMUSG00000039985 | 0 | 0 | -1.64979 | 0.213579 |
| ENSMUSG00000039994 | 0 | 0 | -1.61711 | 0.006829 |
| ENSMUSG00000040128 | 0 | 0 | -2.26293 | 0.131703 |
| ENSMUSG00000040133 | 0 | 0 | 6.636522 | 0.213939 |
| ENSMUSG00000040152 | 0 | 0 | 4.092075 | 0.206966 |
| ENSMUSG00000040181 | 0 | 0 | -3.20561 | 0.112018 |
| ENSMUSG00000040209 | 0 | 0 | -1.99249 | 0.053653 |
| ENSMUSG00000040212 | 0 | 0 | 2.034522 | 0.134874 |
| ENSMUSG00000040219 | 0 | 0 | 2.494206 | 0.077331 |
| ENSMUSG00000040260 | 0 | 0 | -1.58371 | 0.203953 |
| ENSMUSG00000040289 | 0 | 0 | -3.32309 | 0.111913 |
| ENSMUSG00000040296 | 0 | 0 | 2.886001 | 0.079298 |
| ENSMUSG00000040310 | 0 | 0 | -2.28021 | 0.113031 |
| ENSMUSG00000040329 | 0 | 0 | 7.453828 | 2.256328 |
| ENSMUSG00000040350 | 0 | 0 | 5.084888 | 0.259549 |
| ENSMUSG00000040430 | 0 | 0 | -1.58633 | 0.042389 |
| ENSMUSG00000040433 | 0 | 0 | 1.620865 | 0.130557 |
| ENSMUSG00000040537 | 0 | 0 | -1.58803 | 0.239741 |
| ENSMUSG00000040606 | 0 | 0 | -1.59236 | 0.026615 |
| ENSMUSG00000040612 | 0 | 0 | 4.085143 | 0.357014 |
| ENSMUSG00000040618 | 0 | 0 | 2.325791 | 0.459707 |
| ENSMUSG00000040624 | 0 | 0 | -3.93362 | 0.162285 |
| ENSMUSG00000040649 | 0 | 0 | -6.51344 | 0.126908 |
| ENSMUSG00000040653 | 0 | 0 | -3.22446 | 0.172272 |
| ENSMUSG00000040666 | 0 | 0 | 5.151188 | 0.163966 |
| ENSMUSG00000040710 | 0 | 0 | -2.2935 | 0.168449 |
| ENSMUSG00000040714 | 0 | 0 | 3.7227 | 0.027433 |
| ENSMUSG00000040723 | 0 | 0 | -2.58878 | 0.094091 |
| ENSMUSG00000040732 | 0 | 0 | -2.0774 | 0.317271 |
| ENSMUSG00000040746 | 0 | 0 | -1.70419 | 0.090419 |
| ENSMUSG00000040785 | 0 | 0 | -1.78998 | 0.015506 |
| ENSMUSG00000040841 | 0 | 0 | -1.66523 | 0.287774 |
| ENSMUSG00000040856 | 0 | 0 | -3.71767 | 0.230002 |
| ENSMUSG00000040867 | 0 | 0 | -3.22377 | 0.018226 |
| ENSMUSG00000040896 | 0 | 0 | -1.82225 | 0.236266 |
| ENSMUSG00000040918 | 0 | 0 | 2.406428 | 0.01301 |
| ENSMUSG00000041073 | 0 | 0 | 3.873824 | 0.116404 |
| ENSMUSG00000041112 | 0 | 0 | -2.48802 | 0.114849 |
| ENSMUSG00000041119 | 0 | 0 | -2.91401 | 0.141621 |
| ENSMUSG00000041126 | 0 | 0 | -1.69926 | 0.315006 |
| ENSMUSG00000041235 | 0 | 0 | -1.86835 | 0.055993 |
| ENSMUSG00000041301 | 0 | 0 | 1.775086 | 0.106246 |
| ENSMUSG00000041313 | 0 | 0 | 1.69757 | 0.024504 |
| ENSMUSG00000041445 | 0 | 0 | -5.31845 | 0.481453 |
| ENSMUSG00000041481 | 0 | 0 | 3.836728 | 0.118251 |
| ENSMUSG00000041482 | 0 | 0 | -3.88586 | 0.048367 |
| ENSMUSG00000041515 | 0 | 0 | -4.95564 | 0.021537 |
| ENSMUSG00000041548 | 0 | 0 | 2.980984 | 0.138872 |
| ENSMUSG00000041559 | 0 | 0 | 3.554736 | 0.448367 |
| ENSMUSG00000041577 | 0 | 0 | 2.730697 | 0.398176 |
| ENSMUSG00000041592 | 0 | 0 | -2.92668 | 0.149712 |
| ENSMUSG00000041607 | 0 | 0 | 2.544976 | 0.237194 |
| ENSMUSG00000041608 | 0 | 0 | 2.171135 | 0.425786 |
| ENSMUSG00000041625 | 0 | 0 | 2.85597 | 0.055477 |
| ENSMUSG00000041642 | 0 | 0 | -1.99938 | 0.443228 |
| ENSMUSG00000041708 | 0 | 0 | -5.84962 | 0.008424 |
| ENSMUSG00000041731 | 0 | 0 | -3.61928 | 0.107828 |
| ENSMUSG00000041736 | 0 | 0 | 3.427686 | 0.153835 |
| ENSMUSG00000041741 | 0 | 0 | -1.50247 | 0.047414 |
| ENSMUSG00000041797 | 0 | 0 | -1.68513 | 0.316516 |
| ENSMUSG00000041801 | 0 | 0 | 3.04945 | 0.132649 |
| ENSMUSG00000041828 | 0 | 0 | -4.9946 | 0.731217 |
| ENSMUSG00000041842 | 0 | 0 | 2.69556 | 0.386264 |
| ENSMUSG00000041886 | 0 | 0 | 1.662914 | 0.361335 |
| ENSMUSG00000041889 | 0 | 0 | 2.47349 | 0.125485 |
| ENSMUSG00000041911 | 0 | 0 | -1.9 | 0.041832 |
| ENSMUSG00000041936 | 0 | 0 | -1.64327 | 0.007229 |
| ENSMUSG00000041957 | 0 | 0 | 6.374226 | 0.340437 |
| ENSMUSG00000041992 | 0 | 0 | -5.00957 | 0.196319 |
| ENSMUSG00000042115 | 0 | 0 | 8.523144 | 0.352095 |
| ENSMUSG00000042116 | 0 | 0 | -2.07991 | 0.175482 |
| ENSMUSG00000042155 | 0 | 0 | -1.94233 | 0.038345 |
| ENSMUSG00000042190 | 0 | 0 | 4.449012 | 0.013597 |
| ENSMUSG00000042195 | 0 | 0 | -2.61749 | 0.233849 |
| ENSMUSG00000042215 | 0 | 0 | 2.809405 | 0.15785 |
| ENSMUSG00000042256 | 0 | 0 | 2.788495 | 0.170491 |
| ENSMUSG00000042286 | 0 | 0 | -5.81803 | 0.050192 |
| ENSMUSG00000042292 | 0 | 0 | 1.768842 | 0.103519 |
| ENSMUSG00000042349 | 0 | 0 | 2.931271 | 0.208569 |
| ENSMUSG00000042363 | 0 | 0 | 1.96072 | 0.10332 |
| ENSMUSG00000042429 | 0 | 0 | 5.722098 | 0.424751 |
| ENSMUSG00000042549 | 0 | 0 | 2.294384 | 0.387234 |
| ENSMUSG00000042599 | 0 | 0 | -1.6997 | 0.300349 |
| ENSMUSG00000042604 | 0 | 0 | -2.60043 | 0.015643 |
| ENSMUSG00000042607 | 0 | 0 | -3.85502 | 0.117158 |
| ENSMUSG00000042613 | 0 | 0 | 1.896526 | 0.146575 |
| ENSMUSG00000042750 | 0 | 0 | -3.58487 | 0.36878 |
| ENSMUSG00000042759 | 0 | 0 | 5.56071 | 0.54492 |
| ENSMUSG00000042766 | 0 | 0 | 2.389321 | 0.41454 |
| ENSMUSG00000042807 | 0 | 0 | -1.59433 | 0.077381 |
| ENSMUSG00000042834 | 0 | 0 | -1.81185 | 0.208261 |
| ENSMUSG00000042961 | 0 | 0 | -3.96099 | 0.250387 |
| ENSMUSG00000042978 | 0 | 0 | -2.3819 | 0.144854 |
| ENSMUSG00000043004 | 0 | 0 | -1.96342 | 0.239702 |
| ENSMUSG00000043008 | 0 | 0 | -4.34435 | 0.373436 |
| ENSMUSG00000043015 | 0 | 0 | -2.54184 | 0.13298 |
| ENSMUSG00000043207 | 0 | 0 | 1.523234 | 0.091658 |
| ENSMUSG00000043311 | 0 | 0 | 1.55684 | 0.159831 |
| ENSMUSG00000043336 | 0 | 0 | 1.837374 | 0.011735 |
| ENSMUSG00000043384 | 0 | 0 | -2.04007 | 0.029928 |
| ENSMUSG00000043419 | 0 | 0 | -2.35328 | 0.082346 |
| ENSMUSG00000043456 | 0 | 0 | -2.79088 | 0.114598 |
| ENSMUSG00000043496 | 0 | 0 | -6.57036 | 0.026905 |
| ENSMUSG00000043531 | 0 | 0 | 1.670593 | 0.220948 |
| ENSMUSG00000043613 | 0 | 0 | 3.33835 | 0.16508 |
| ENSMUSG00000043635 | 0 | 0 | -1.58991 | 0.158111 |
| ENSMUSG00000043822 | 0 | 0 | 7.35484 | 0.512491 |
| ENSMUSG00000043881 | 0 | 0 | -1.66949 | 0.169361 |
| ENSMUSG00000044042 | 0 | 0 | 3.739282 | 0.05357 |
| ENSMUSG00000044164 | 0 | 0 | -5.15654 | 0.01641 |
| ENSMUSG00000044177 | 0 | 0 | -2.29861 | 0.199831 |
| ENSMUSG00000044197 | 0 | 0 | -2.79862 | 0.105582 |
| ENSMUSG00000044254 | 0 | 0 | 4.061851 | 0.073136 |
| ENSMUSG00000044258 | 0 | 0 | -2.83639 | 0.044343 |
| ENSMUSG00000044338 | 0 | 0 | -11.2106 | 1.454443 |
| ENSMUSG00000044447 | 0 | 0 | 1.787075 | 0.092435 |
| ENSMUSG00000044461 | 0 | 0 | -2.72253 | 0.038789 |
| ENSMUSG00000044468 | 0 | 0 | -3.00967 | 0.220951 |
| ENSMUSG00000044551 | 0 | 0 | 1.7976 | 0.259976 |
| ENSMUSG00000044562 | 0 | 0 | -6.95804 | 0.169844 |
| ENSMUSG00000044647 | 0 | 0 | -4.44593 | 0.529078 |
| ENSMUSG00000044712 | 0 | 0 | -1.8454 | 0.005073 |
| ENSMUSG00000044881 | 0 | 0 | 2.196692 | 0.186065 |
| ENSMUSG00000044927 | 0 | 0 | -2.49094 | 0.233654 |
| ENSMUSG00000045062 | 0 | 0 | -1.62401 | 0.056575 |
| ENSMUSG00000045071 | 0 | 0 | -1.66332 | 0.021494 |
| ENSMUSG00000045251 | 0 | 0 | 1.966572 | 0.061666 |
| ENSMUSG00000045333 | 0 | 0 | -2.40596 | 0.149815 |
| ENSMUSG00000045374 | 0 | 0 | 1.548677 | 0.063994 |
| ENSMUSG00000045377 | 0 | 0 | 1.945575 | 0.125789 |
| ENSMUSG00000045394 | 0 | 0 | -7.06329 | 2.304999 |
| ENSMUSG00000045441 | 0 | 0 | 1.697491 | 0.016476 |
| ENSMUSG00000045545 | 0 | 0 | -8.61323 | 0.768664 |
| ENSMUSG00000045573 | 0 | 0 | -5.88139 | 0.059555 |
| ENSMUSG00000045589 | 0 | 0 | -2.94336 | 0.126963 |
| ENSMUSG00000045664 | 0 | 0 | 2.525949 | 0.054118 |
| ENSMUSG00000045665 | 0 | 0 | 1.785087 | 0.071568 |
| ENSMUSG00000045679 | 0 | 0 | 3.801898 | 0.184889 |
| ENSMUSG00000045730 | 0 | 0 | 2.387856 | 0.164319 |
| ENSMUSG00000045827 | 0 | 0 | 1.665098 | 0.146675 |
| ENSMUSG00000045838 | 0 | 0 | 5.272602 | 0.175616 |
| ENSMUSG00000045930 | 0 | 0 | -3.18963 | 0.303756 |
| ENSMUSG00000045954 | 0 | 0 | 4.791766 | 0.648452 |
| ENSMUSG00000045980 | 0 | 0 | 2.625782 | 0.090665 |
| ENSMUSG00000046157 | 0 | 0 | -1.56237 | 0.010379 |
| ENSMUSG00000046186 | 0 | 0 | 3.883668 | 0.160034 |
| ENSMUSG00000046223 | 0 | 0 | 3.633051 | 0.535085 |
| ENSMUSG00000046269 | 0 | 0 | -1.61335 | 0.124744 |
| ENSMUSG00000046280 | 0 | 0 | -6.8824 | 0.901685 |
| ENSMUSG00000046312 | 0 | 0 | 2.968748 | 0.238338 |
| ENSMUSG00000046314 | 0 | 0 | -1.66998 | 0.272663 |
| ENSMUSG00000046402 | 0 | 0 | -2.40963 | 0.076911 |
| ENSMUSG00000046434 | 0 | 0 | -1.70639 | 0.071154 |
| ENSMUSG00000046447 | 0 | 0 | -2.22233 | 0.124169 |
| ENSMUSG00000046618 | 0 | 0 | -4.83699 | 0.105432 |
| ENSMUSG00000046694 | 0 | 0 | 5.09283 | 0.123662 |
| ENSMUSG00000046718 | 0 | 0 | 2.12374 | 0.077251 |
| ENSMUSG00000046731 | 0 | 0 | 1.782453 | 0.278325 |
| ENSMUSG00000046761 | 0 | 0 | 3.090916 | 0.112617 |
| ENSMUSG00000046768 | 0 | 0 | 2.329534 | 0.065988 |
| ENSMUSG00000046794 | 0 | 0 | -1.71833 | 0.199665 |
| ENSMUSG00000046807 | 0 | 0 | 3.98597 | 0.166501 |
| ENSMUSG00000046814 | 0 | 0 | 4.864696 | 0.495213 |
| ENSMUSG00000046876 | 0 | 0 | 2.574346 | 0.02684 |
| ENSMUSG00000046879 | 0 | 0 | 1.612957 | 0.013417 |
| ENSMUSG00000046982 | 0 | 0 | -2.0078 | 0.184689 |
| ENSMUSG00000047085 | 0 | 0 | -5.50554 | 0.267495 |
| ENSMUSG00000047123 | 0 | 0 | 1.613149 | 0.368152 |
| ENSMUSG00000047139 | 0 | 0 | -2.33696 | 0.26292 |
| ENSMUSG00000047146 | 0 | 0 | -1.52349 | 0.037614 |
| ENSMUSG00000047181 | 0 | 0 | -1.84326 | 0.168213 |
| ENSMUSG00000047246 | 0 | 0 | -1.85992 | 0.057892 |
| ENSMUSG00000047250 | 0 | 0 | 2.292596 | 0.188292 |
| ENSMUSG00000047428 | 0 | 0 | 2.86401 | 0.386218 |
| ENSMUSG00000047443 | 0 | 0 | 3.456573 | 0.434184 |
| ENSMUSG00000047604 | 0 | 0 | -3.5018 | 0.177804 |
| ENSMUSG00000047747 | 0 | 0 | 2.99654 | 0.26241 |
| ENSMUSG00000047749 | 0 | 0 | -1.54821 | 0.069634 |
| ENSMUSG00000047786 | 0 | 0 | -2.08403 | 0.018824 |
| ENSMUSG00000047793 | 0 | 0 | -1.67087 | 0.444337 |
| ENSMUSG00000047867 | 0 | 0 | -6.0423 | 0.206265 |
| ENSMUSG00000047878 | 0 | 0 | 7.500838 | 1.774124 |
| ENSMUSG00000047907 | 0 | 0 | -2.34235 | 0.024304 |
| ENSMUSG00000047976 | 0 | 0 | -3.14524 | 0.118402 |
| ENSMUSG00000048040 | 0 | 0 | 1.538684 | 0.141204 |
| ENSMUSG00000048065 | 0 | 0 | 3.325141 | 0.48241 |
| ENSMUSG00000048096 | 0 | 0 | 7.16105 | 0.334671 |
| ENSMUSG00000048120 | 0 | 0 | -2.70466 | 0.358959 |
| ENSMUSG00000048277 | 0 | 0 | 1.693414 | 0.088367 |
| ENSMUSG00000048332 | 0 | 0 | 2.446686 | 0.13605 |
| ENSMUSG00000048458 | 0 | 0 | 5.93537 | 0.176032 |
| ENSMUSG00000048482 | 0 | 0 | 1.730026 | 0.423842 |
| ENSMUSG00000048572 | 0 | 0 | -3.47248 | 0.216204 |
| ENSMUSG00000048612 | 0 | 0 | 3.860869 | 0.041757 |
| ENSMUSG00000048706 | 0 | 0 | 4.637346 | 0.216316 |
| ENSMUSG00000048772 | 0 | 0 | 1.920305 | 0.295542 |
| ENSMUSG00000048814 | 0 | 0 | -2.17282 | 0.068864 |
| ENSMUSG00000048834 | 0 | 0 | -1.92055 | 0.046797 |
| ENSMUSG00000048960 | 0 | 0 | -2.0745 | 0.195153 |
| ENSMUSG00000048997 | 0 | 0 | -1.6968 | 0.208953 |
| ENSMUSG00000049100 | 0 | 0 | -2.1004 | 0.066394 |
| ENSMUSG00000049225 | 0 | 0 | -1.7009 | 0.058827 |
| ENSMUSG00000049252 | 0 | 0 | -2.82261 | 0.131316 |
| ENSMUSG00000049281 | 0 | 0 | 2.717242 | 0.60427 |
| ENSMUSG00000049336 | 0 | 0 | -4.05332 | 0.121639 |
| ENSMUSG00000049396 | 0 | 0 | 1.816291 | 0.110839 |
| ENSMUSG00000049404 | 0 | 0 | 4.300927 | 0.191118 |
| ENSMUSG00000049409 | 0 | 0 | -2.60117 | 0.265329 |
| ENSMUSG00000049511 | 0 | 0 | 3.46084 | 0.293608 |
| ENSMUSG00000049516 | 0 | 0 | -1.52116 | 0.170825 |
| ENSMUSG00000049717 | 0 | 0 | 1.561954 | 0.05575 |
| ENSMUSG00000049823 | 0 | 0 | -1.55244 | 0.042954 |
| ENSMUSG00000049872 | 0 | 0 | 3.884097 | 0.224716 |
| ENSMUSG00000049950 | 0 | 0 | 1.973377 | 0.199691 |
| ENSMUSG00000050071 | 0 | 0 | -3.93129 | 0.614019 |
| ENSMUSG00000050088 | 0 | 0 | 1.691094 | 0.037614 |
| ENSMUSG00000050188 | 0 | 0 | 1.803036 | 0.259936 |
| ENSMUSG00000050240 | 0 | 0 | -2.40104 | 0.007472 |
| ENSMUSG00000050335 | 0 | 0 | 5.077985 | 0.083437 |
| ENSMUSG00000050370 | 0 | 0 | -2.54973 | 0.124847 |
| ENSMUSG00000050505 | 0 | 0 | -3.05007 | 0.459436 |
| ENSMUSG00000050578 | 0 | 0 | 1.867517 | 0.244289 |
| ENSMUSG00000050666 | 0 | 0 | -1.82641 | 0.272872 |
| ENSMUSG00000050700 | 0 | 0 | -10.6488 | 2.163904 |
| ENSMUSG00000050721 | 0 | 0 | 1.823926 | 0.022877 |
| ENSMUSG00000050751 | 0 | 0 | -3.05244 | 0.218906 |
| ENSMUSG00000050777 | 0 | 0 | 4.431035 | 0.280835 |
| ENSMUSG00000050912 | 0 | 0 | 1.702136 | 0.088145 |
| ENSMUSG00000050947 | 0 | 0 | -1.56964 | 0.481594 |
| ENSMUSG00000050973 | 0 | 0 | 1.579149 | 0.135925 |
| ENSMUSG00000051041 | 0 | 0 | -2.74008 | 0.157376 |
| ENSMUSG00000051043 | 0 | 0 | -1.88547 | 0.20458 |
| ENSMUSG00000051147 | 0 | 0 | 2.406914 | 0.315452 |
| ENSMUSG00000051159 | 0 | 0 | -3.91657 | 0.16323 |
| ENSMUSG00000051236 | 0 | 0 | 2.588952 | 0.118618 |
| ENSMUSG00000051316 | 0 | 0 | -2.44109 | 0.054218 |
| ENSMUSG00000051319 | 0 | 0 | 1.847024 | 0.117145 |
| ENSMUSG00000051323 | 0 | 0 | 1.588012 | 0.025772 |
| ENSMUSG00000051339 | 0 | 0 | 1.738186 | 0.048167 |
| ENSMUSG00000051375 | 0 | 0 | -5.03436 | 0.329428 |
| ENSMUSG00000051726 | 0 | 0 | 3.794216 | 0.537279 |
| ENSMUSG00000051811 | 0 | 0 | 2.948561 | 0.475555 |
| ENSMUSG00000051817 | 0 | 0 | -2.20899 | 0.118776 |
| ENSMUSG00000051978 | 0 | 0 | 1.552338 | 0.118878 |
| ENSMUSG00000052151 | 0 | 0 | 1.873617 | 0.119339 |
| ENSMUSG00000052187 | 0 | 0 | -12.6745 | 0.125489 |
| ENSMUSG00000052295 | 0 | 0 | 2.133117 | 0.157624 |
| ENSMUSG00000052305 | 0 | 0 | -12.37 | 0 |
| ENSMUSG00000052316 | 0 | 0 | 6.449992 | 0.079228 |
| ENSMUSG00000052336 | 0 | 0 | -1.72785 | 0.04993 |
| ENSMUSG00000052353 | 0 | 0 | 6.617348 | 0.215239 |
| ENSMUSG00000052384 | 0 | 0 | -4.26916 | 0.116643 |
| ENSMUSG00000052430 | 0 | 0 | 1.661116 | 0.005814 |
| ENSMUSG00000052544 | 0 | 0 | -3.48483 | 0.05194 |
| ENSMUSG00000052609 | 0 | 0 | 2.193143 | 0.090447 |
| ENSMUSG00000052613 | 0 | 0 | -1.83209 | 0.270334 |
| ENSMUSG00000052632 | 0 | 0 | -1.60197 | 0.012434 |
| ENSMUSG00000052684 | 0 | 0 | -2.37355 | 0.074235 |
| ENSMUSG00000052688 | 0 | 0 | 2.49182 | 0.39601 |
| ENSMUSG00000052942 | 0 | 0 | 2.171653 | 0.258945 |
| ENSMUSG00000053062 | 0 | 0 | 2.113536 | 0.126832 |
| ENSMUSG00000053063 | 0 | 0 | -2.91522 | 0.422432 |
| ENSMUSG00000053113 | 0 | 0 | -2.13467 | 0.247141 |
| ENSMUSG00000053199 | 0 | 0 | -2.2349 | 0.167688 |
| ENSMUSG00000053279 | 0 | 0 | 2.992418 | 0.318715 |
| ENSMUSG00000053334 | 0 | 0 | 2.696841 | 0.004614 |
| ENSMUSG00000053399 | 0 | 0 | 2.40064 | 0.765814 |
| ENSMUSG00000053414 | 0 | 0 | -2.8917 | 0.012153 |
| ENSMUSG00000053469 | 0 | 0 | 3.657084 | 0.266711 |
| ENSMUSG00000053522 | 0 | 0 | -3.50885 | 0.058281 |
| ENSMUSG00000053552 | 0 | 0 | -1.73734 | 0.034849 |
| ENSMUSG00000053604 | 0 | 0 | -1.52357 | 0.108179 |
| ENSMUSG00000053615 | 0 | 0 | 2.542706 | 0.054278 |
| ENSMUSG00000053626 | 0 | 0 | 2.177669 | 0.029957 |
| ENSMUSG00000053647 | 0 | 0 | 2.989592 | 0.293166 |
| ENSMUSG00000053746 | 0 | 0 | 4.389697 | 0.110448 |
| ENSMUSG00000053846 | 0 | 0 | 2.223797 | 0.308123 |
| ENSMUSG00000053897 | 0 | 0 | -1.7174 | 0.094621 |
| ENSMUSG00000053965 | 0 | 0 | -1.70924 | 0.064453 |
| ENSMUSG00000054072 | 0 | 0 | -4.96229 | 0.519812 |
| ENSMUSG00000054115 | 0 | 0 | -1.55584 | 0.151695 |
| ENSMUSG00000054150 | 0 | 0 | 2.12011 | 0.133521 |
| ENSMUSG00000054252 | 0 | 0 | -4.16895 | 0.360427 |
| ENSMUSG00000054293 | 0 | 0 | 2.901919 | 0.12478 |
| ENSMUSG00000054435 | 0 | 0 | -9.31992 | 1.062533 |
| ENSMUSG00000054474 | 0 | 0 | 1.727975 | 0.321426 |
| ENSMUSG00000054484 | 0 | 0 | 1.821796 | 0.061964 |
| ENSMUSG00000054509 | 0 | 0 | 1.978842 | 0.205617 |
| ENSMUSG00000054520 | 0 | 0 | -1.69228 | 0.004096 |
| ENSMUSG00000054555 | 0 | 0 | 2.492043 | 0.074082 |
| ENSMUSG00000054580 | 0 | 0 | 2.566162 | 0.048508 |
| ENSMUSG00000054612 | 0 | 0 | 2.348598 | 0.019976 |
| ENSMUSG00000054641 | 0 | 0 | -4.67915 | 0.525466 |
| ENSMUSG00000054690 | 0 | 0 | -4.75918 | 0.166948 |
| ENSMUSG00000054717 | 0 | 0 | -2.11441 | 0.009429 |
| ENSMUSG00000054793 | 0 | 0 | -4.94793 | 0.08085 |
| ENSMUSG00000054855 | 0 | 0 | 1.649421 | 0.322822 |
| ENSMUSG00000054871 | 0 | 0 | -3.38879 | 0.025173 |
| ENSMUSG00000054889 | 0 | 0 | -3.72786 | 0.785503 |
| ENSMUSG00000054951 | 0 | 0 | 2.78802 | 0.388581 |
| ENSMUSG00000054988 | 0 | 0 | 1.943268 | 0.330147 |
| ENSMUSG00000055022 | 0 | 0 | -1.53541 | 0.167283 |
| ENSMUSG00000055041 | 0 | 0 | 1.523385 | 0.178103 |
| ENSMUSG00000055044 | 0 | 0 | 4.923807 | 0.32614 |
| ENSMUSG00000055116 | 0 | 0 | 1.732271 | 0.01998 |
| ENSMUSG00000055235 | 0 | 0 | -8.13392 | 0.436152 |
| ENSMUSG00000055254 | 0 | 0 | -5.14003 | 0.351275 |
| ENSMUSG00000055301 | 0 | 0 | 4.815957 | 0.124373 |
| ENSMUSG00000055401 | 0 | 0 | 3.492115 | 0.108869 |
| ENSMUSG00000055407 | 0 | 0 | 5.882912 | 0.008601 |
| ENSMUSG00000055447 | 0 | 0 | 2.013199 | 0.040497 |
| ENSMUSG00000055540 | 0 | 0 | -5.96914 | 0.200326 |
| ENSMUSG00000055612 | 0 | 0 | -2.01494 | 0.184529 |
| ENSMUSG00000055629 | 0 | 0 | -4.74226 | 0.005426 |
| ENSMUSG00000055670 | 0 | 0 | 1.572983 | 0.212443 |
| ENSMUSG00000055866 | 0 | 0 | -2.84034 | 0.117607 |
| ENSMUSG00000055912 | 0 | 0 | 2.140244 | 0.410644 |
| ENSMUSG00000056145 | 0 | 0 | -2.36373 | 0.378567 |
| ENSMUSG00000056222 | 0 | 0 | -5.86284 | 0.723846 |
| ENSMUSG00000056313 | 0 | 0 | -3.66709 | 0.196385 |
| ENSMUSG00000056492 | 0 | 0 | -5.08472 | 0.151064 |
| ENSMUSG00000056596 | 0 | 0 | 4.885013 | 0.194694 |
| ENSMUSG00000056737 | 0 | 0 | 1.600317 | 0.23705 |
| ENSMUSG00000056888 | 0 | 0 | 5.472771 | 0.185509 |
| ENSMUSG00000056895 | 0 | 0 | -3.51279 | 0.031614 |
| ENSMUSG00000056938 | 0 | 0 | 2.37477 | 0.03526 |
| ENSMUSG00000057098 | 0 | 0 | -2.4877 | 0.188883 |
| ENSMUSG00000057123 | 0 | 0 | -2.9305 | 0.124804 |
| ENSMUSG00000057137 | 0 | 0 | 3.034054 | 0.193419 |
| ENSMUSG00000057182 | 0 | 0 | -2.16836 | 0.21186 |
| ENSMUSG00000057329 | 0 | 0 | -2.52089 | 0.362945 |
| ENSMUSG00000057337 | 0 | 0 | -3.4354 | 0.110767 |
| ENSMUSG00000057457 | 0 | 0 | -3.19733 | 0.296855 |
| ENSMUSG00000057554 | 0 | 0 | 1.558358 | 0.10714 |
| ENSMUSG00000058145 | 0 | 0 | -3.0147 | 0.014928 |
| ENSMUSG00000058297 | 0 | 0 | -3.30944 | 0.321835 |
| ENSMUSG00000058806 | 0 | 0 | -6.48006 | 0.092023 |
| ENSMUSG00000058897 | 0 | 0 | -4.86653 | 0.191052 |
| ENSMUSG00000059005 | 0 | 0 | -1.8607 | 0.155785 |
| ENSMUSG00000059022 | 0 | 0 | -3.46017 | 0.525008 |
| ENSMUSG00000059049 | 0 | 0 | -3.96042 | 0.475989 |
| ENSMUSG00000059430 | 0 | 0 | 9.108758 | 0.149274 |
| ENSMUSG00000059456 | 0 | 0 | 3.074749 | 0.506269 |
| ENSMUSG00000059474 | 0 | 0 | -1.7493 | 0.051797 |
| ENSMUSG00000059555 | 0 | 0 | 4.208976 | 0.080374 |
| ENSMUSG00000059674 | 0 | 0 | -1.80598 | 0.529373 |
| ENSMUSG00000059714 | 0 | 0 | 1.588284 | 0.001935 |
| ENSMUSG00000059857 | 0 | 0 | -2.99478 | 0.284271 |
| ENSMUSG00000059883 | 0 | 0 | 1.789511 | 0.011606 |
| ENSMUSG00000060032 | 0 | 0 | 1.781592 | 0.094635 |
| ENSMUSG00000060044 | 0 | 0 | -3.17617 | 0.16734 |
| ENSMUSG00000060147 | 0 | 0 | 2.082091 | 0.208567 |
| ENSMUSG00000060240 | 0 | 0 | 5.010776 | 0.08095 |
| ENSMUSG00000060260 | 0 | 0 | 2.119921 | 0.068113 |
| ENSMUSG00000060284 | 0 | 0 | -4.90311 | 0.517911 |
| ENSMUSG00000060402 | 0 | 0 | -2.10646 | 0.350962 |
| ENSMUSG00000060477 | 0 | 0 | 3.002627 | 0.221225 |
| ENSMUSG00000060519 | 0 | 0 | 3.134698 | 0.017343 |
| ENSMUSG00000060534 | 0 | 0 | -2.28222 | 0.409 |
| ENSMUSG00000060548 | 0 | 0 | -5.84594 | 0.116779 |
| ENSMUSG00000060572 | 0 | 0 | -1.66778 | 0.348072 |
| ENSMUSG00000060600 | 0 | 0 | 3.092161 | 0.330825 |
| ENSMUSG00000060675 | 0 | 0 | 2.561151 | 0.35859 |
| ENSMUSG00000060703 | 0 | 0 | -1.53331 | 0.017078 |
| ENSMUSG00000060743 | 0 | 0 | -1.51516 | 0.039737 |
| ENSMUSG00000060780 | 0 | 0 | -5.46834 | 0.171119 |
| ENSMUSG00000060802 | 0 | 0 | 2.001196 | 0.009948 |
| ENSMUSG00000060961 | 0 | 0 | 1.786671 | 0.012605 |
| ENSMUSG00000061080 | 0 | 0 | -2.1654 | 0.161921 |
| ENSMUSG00000061143 | 0 | 0 | -2.39868 | 0.044954 |
| ENSMUSG00000061232 | 0 | 0 | 1.538859 | 0.240292 |
| ENSMUSG00000061527 | 0 | 0 | -9.78399 | 1.889827 |
| ENSMUSG00000061535 | 0 | 0 | -3.6064 | 0.191019 |
| ENSMUSG00000061603 | 0 | 0 | 1.912314 | 0.060185 |
| ENSMUSG00000061758 | 0 | 0 | 2.774839 | 0.110471 |
| ENSMUSG00000061878 | 0 | 0 | 2.173522 | 0.016094 |
| ENSMUSG00000062175 | 0 | 0 | -1.60337 | 0.005353 |
| ENSMUSG00000062345 | 0 | 0 | 3.881877 | 0.085784 |
| ENSMUSG00000062380 | 0 | 0 | 2.395454 | 0.044857 |
| ENSMUSG00000062753 | 0 | 0 | 2.839189 | 0.362711 |
| ENSMUSG00000062960 | 0 | 0 | -5.60873 | 0.869883 |
| ENSMUSG00000063268 | 0 | 0 | 3.378271 | 0.383695 |
| ENSMUSG00000063275 | 0 | 0 | 2.327881 | 0.173297 |
| ENSMUSG00000063415 | 0 | 0 | -2.58625 | 0.028243 |
| ENSMUSG00000063445 | 0 | 0 | -1.74864 | 0.027239 |
| ENSMUSG00000063450 | 0 | 0 | -1.95287 | 0.002126 |
| ENSMUSG00000063455 | 0 | 0 | -1.62058 | 0.205477 |
| ENSMUSG00000063506 | 0 | 0 | 4.165411 | 0.402892 |
| ENSMUSG00000063558 | 0 | 0 | 3.934673 | 0.15043 |
| ENSMUSG00000063564 | 0 | 0 | -3.94212 | 0.525882 |
| ENSMUSG00000063632 | 0 | 0 | -1.83237 | 0.107728 |
| ENSMUSG00000063689 | 0 | 0 | -2.17437 | 0.008934 |
| ENSMUSG00000063727 | 0 | 0 | 5.79491 | 0.033816 |
| ENSMUSG00000063838 | 0 | 0 | 1.570061 | 0.047331 |
| ENSMUSG00000063873 | 0 | 0 | 2.830915 | 0.030882 |
| ENSMUSG00000064141 | 0 | 0 | -1.71046 | 0.036454 |
| ENSMUSG00000064264 | 0 | 0 | -1.63514 | 0.271761 |
| ENSMUSG00000064284 | 0 | 0 | 1.864109 | 0.011839 |
| ENSMUSG00000064293 | 0 | 0 | -1.90382 | 0.035586 |
| ENSMUSG00000064373 | 0 | 0 | -2.31656 | 0.144391 |
| ENSMUSG00000066551 | 0 | 0 | -1.71004 | 0.017917 |
| ENSMUSG00000066705 | 0 | 0 | -2.45816 | 0.089312 |
| ENSMUSG00000066755 | 0 | 0 | 4.410946 | 0.917489 |
| ENSMUSG00000066952 | 0 | 0 | 3.26101 | 0.202637 |
| ENSMUSG00000067001 | 0 | 0 | 3.058165 | 0.361353 |
| ENSMUSG00000067276 | 0 | 0 | -2.00299 | 0.230702 |
| ENSMUSG00000067455 | 0 | 0 | -1.51145 | 0.006429 |
| ENSMUSG00000067786 | 0 | 0 | -3.7649 | 0.162997 |
| ENSMUSG00000067818 | 0 | 0 | 2.666987 | 0.016206 |
| ENSMUSG00000068196 | 0 | 0 | 4.095622 | 0.028536 |
| ENSMUSG00000068220 | 0 | 0 | 1.551865 | 0.069945 |
| ENSMUSG00000068335 | 0 | 0 | 2.965195 | 0.147148 |
| ENSMUSG00000068522 | 0 | 0 | -2.94611 | 0.424447 |
| ENSMUSG00000068566 | 0 | 0 | 2.568264 | 0.079289 |
| ENSMUSG00000068606 | 0 | 0 | -4.01343 | 0.296099 |
| ENSMUSG00000068614 | 0 | 0 | -1.5736 | 0.420563 |
| ENSMUSG00000068740 | 0 | 0 | -3.14062 | 0.270469 |
| ENSMUSG00000068742 | 0 | 0 | -1.52009 | 0.157144 |
| ENSMUSG00000068758 | 0 | 0 | 4.069357 | 0.234347 |
| ENSMUSG00000068874 | 0 | 0 | -3.12048 | 0.128607 |
| ENSMUSG00000069114 | 0 | 0 | -1.8106 | 0.077553 |
| ENSMUSG00000069303 | 0 | 0 | -1.63869 | 0.039801 |
| ENSMUSG00000069308 | 0 | 0 | -1.77473 | 0.103454 |
| ENSMUSG00000069633 | 0 | 0 | 1.631828 | 0.063736 |
| ENSMUSG00000069763 | 0 | 0 | -4.46613 | 0.47138 |
| ENSMUSG00000069769 | 0 | 0 | -2.17379 | 0.0282 |
| ENSMUSG00000069893 | 0 | 0 | 4.4381 | 0.762244 |
| ENSMUSG00000069917 | 0 | 0 | -14.6321 | 1.866043 |
| ENSMUSG00000069919 | 0 | 0 | -11.4468 | 0.257201 |
| ENSMUSG00000070304 | 0 | 0 | -4.42749 | 0.309605 |
| ENSMUSG00000070327 | 0 | 0 | 1.525825 | 0.199857 |
| ENSMUSG00000070348 | 0 | 0 | 1.587813 | 0.144086 |
| ENSMUSG00000070469 | 0 | 0 | 2.332685 | 0.459066 |
| ENSMUSG00000070498 | 0 | 0 | -7.31036 | 0.032914 |
| ENSMUSG00000070527 | 0 | 0 | -2.12941 | 0.128606 |
| ENSMUSG00000070713 | 0 | 0 | -1.64407 | 0.06619 |
| ENSMUSG00000070720 | 0 | 0 | -1.83633 | 0.154842 |
| ENSMUSG00000070802 | 0 | 0 | -3.51565 | 0.195803 |
| ENSMUSG00000070822 | 0 | 0 | -2.32501 | 0.276652 |
| ENSMUSG00000070942 | 0 | 0 | 1.798391 | 0.35262 |
| ENSMUSG00000071076 | 0 | 0 | -1.54941 | 0.066591 |
| ENSMUSG00000071083 | 0 | 0 | 1.722496 | 0.343208 |
| ENSMUSG00000071337 | 0 | 0 | -1.51665 | 0.091939 |
| ENSMUSG00000071369 | 0 | 0 | -1.75723 | 0.123426 |
| ENSMUSG00000071656 | 0 | 0 | 1.720313 | 0.047822 |
| ENSMUSG00000071847 | 0 | 0 | -1.83632 | 0.134196 |
| ENSMUSG00000071984 | 0 | 0 | 2.562297 | 0.248993 |
| ENSMUSG00000072437 | 0 | 0 | 1.936287 | 0.270837 |
| ENSMUSG00000072674 | 0 | 0 | 3.21047 | 0.300019 |
| ENSMUSG00000072680 | 0 | 0 | 1.580314 | 0.201974 |
| ENSMUSG00000072812 | 0 | 0 | 9.410481 | 0.34428 |
| ENSMUSG00000072893 | 0 | 0 | -1.79219 | 0.027822 |
| ENSMUSG00000072944 | 0 | 0 | 2.707916 | 0.30773 |
| ENSMUSG00000072966 | 0 | 0 | -2.97026 | 0.0024 |
| ENSMUSG00000073274 | 0 | 0 | 4.382293 | 0.337515 |
| ENSMUSG00000073489 | 0 | 0 | 2.872867 | 0.13989 |
| ENSMUSG00000073530 | 0 | 0 | -2.74539 | 0.391069 |
| ENSMUSG00000073643 | 0 | 0 | 1.581924 | 0.073036 |
| ENSMUSG00000073771 | 0 | 0 | 3.129778 | 0.249068 |
| ENSMUSG00000073802 | 0 | 0 | 7.573996 | 0.153504 |
| ENSMUSG00000073888 | 0 | 0 | -1.7532 | 0.158284 |
| ENSMUSG00000073889 | 0 | 0 | -1.5118 | 0.235624 |
| ENSMUSG00000073910 | 0 | 0 | -2.08511 | 0.015872 |
| ENSMUSG00000073940 | 0 | 0 | -12.9684 | 0.524437 |
| ENSMUSG00000074364 | 0 | 0 | 1.860926 | 0.100615 |
| ENSMUSG00000074457 | 0 | 0 | -1.89083 | 0.168385 |
| ENSMUSG00000074480 | 0 | 0 | -1.91576 | 0.116737 |
| ENSMUSG00000074505 | 0 | 0 | -2.58783 | 0.029176 |
| ENSMUSG00000074577 | 0 | 0 | -3.15354 | 0.182705 |
| ENSMUSG00000074622 | 0 | 0 | -1.6404 | 0.297306 |
| ENSMUSG00000074676 | 0 | 0 | 4.160836 | 0.488332 |
| ENSMUSG00000074743 | 0 | 0 | 1.92886 | 0.087878 |
| ENSMUSG00000074794 | 0 | 0 | -1.77211 | 0.011656 |
| ENSMUSG00000074807 | 0 | 0 | -1.74544 | 0.027388 |
| ENSMUSG00000074811 | 0 | 0 | 2.398598 | 0.062394 |
| ENSMUSG00000074813 | 0 | 0 | 5.892574 | 0.415705 |
| ENSMUSG00000074934 | 0 | 0 | 5.948227 | 0.096884 |
| ENSMUSG00000074968 | 0 | 0 | 2.921485 | 0.19077 |
| ENSMUSG00000075028 | 0 | 0 | -2.01192 | 0.050345 |
| ENSMUSG00000075334 | 0 | 0 | 2.061246 | 0.507443 |
| ENSMUSG00000075602 | 0 | 0 | 6.453143 | 0.284167 |
| ENSMUSG00000075702 | 0 | 0 | 1.594637 | 0.079124 |
| ENSMUSG00000075707 | 0 | 0 | -1.72254 | 0.194779 |
| ENSMUSG00000076441 | 0 | 0 | 5.993798 | 0.299441 |
| ENSMUSG00000078202 | 0 | 0 | -6.00088 | 0.491982 |
| ENSMUSG00000078317 | 0 | 0 | 1.793503 | 0.231144 |
| ENSMUSG00000078453 | 0 | 0 | 1.59949 | 0.207506 |
| ENSMUSG00000078747 | 0 | 0 | -3.70582 | 0.045026 |
| ENSMUSG00000078851 | 0 | 0 | -1.67169 | 0.040621 |
| ENSMUSG00000078853 | 0 | 0 | -2.33173 | 0.509213 |
| ENSMUSG00000078877 | 0 | 0 | 1.525951 | 0.077771 |
| ENSMUSG00000078891 | 0 | 0 | 2.363038 | 0.406983 |
| ENSMUSG00000078920 | 0 | 0 | 2.147552 | 0.385575 |
| ENSMUSG00000078922 | 0 | 0 | 1.973883 | 0.280644 |
| ENSMUSG00000079014 | 0 | 0 | 4.325147 | 0.40757 |
| ENSMUSG00000079037 | 0 | 0 | 1.7491 | 0.031959 |
| ENSMUSG00000079055 | 0 | 0 | -3.55418 | 0.035511 |
| ENSMUSG00000079057 | 0 | 0 | 2.103442 | 0.23948 |
| ENSMUSG00000079242 | 0 | 0 | -1.86585 | 0.047551 |
| ENSMUSG00000079317 | 0 | 0 | 1.61328 | 0.357852 |
| ENSMUSG00000079355 | 0 | 0 | 2.321815 | 0.028277 |
| ENSMUSG00000079470 | 0 | 0 | -1.69054 | 0.09025 |
| ENSMUSG00000079484 | 0 | 0 | 3.205537 | 0.550607 |
| ENSMUSG00000079499 | 0 | 0 | 2.314261 | 0.083619 |
| ENSMUSG00000079592 | 0 | 0 | 2.348463 | 0.310912 |
| ENSMUSG00000079662 | 0 | 0 | -3.39682 | 0.323583 |
| ENSMUSG00000080058 | 0 | 0 | -2.00053 | 0.272195 |
| ENSMUSG00000080115 | 0 | 0 | 2.383328 | 0.363925 |
| ENSMUSG00000080268 | 0 | 0 | 1.637626 | 0.224505 |
| ENSMUSG00000081534 | 0 | 0 | 1.782108 | 0.015174 |
| ENSMUSG00000081683 | 0 | 0 | -5.31107 | 0.325224 |
| ENSMUSG00000084910 | 0 | 0 | -1.62779 | 0.170267 |
| ENSMUSG00000084946 | 0 | 0 | -3.74615 | 0.433962 |
| ENSMUSG00000085148 | 0 | 0 | 2.961611 | 0.15742 |
| ENSMUSG00000085396 | 0 | 0 | -2.4171 | 0.416105 |
| ENSMUSG00000086070 | 0 | 0 | 3.187986 | 0.17341 |
| ENSMUSG00000086502 | 0 | 0 | 1.784144 | 0.017163 |
| ENSMUSG00000086843 | 0 | 0 | 3.086717 | 0.119566 |
| ENSMUSG00000087006 | 0 | 0 | -3.27656 | 0.032443 |
| ENSMUSG00000087141 | 0 | 0 | 2.484768 | 0.095272 |
| ENSMUSG00000087177 | 0 | 0 | -2.11871 | 0.079496 |
| ENSMUSG00000087535 | 0 | 0 | -3.87344 | 0.211743 |
| ENSMUSG00000089901 | 0 | 0 | 2.46904 | 0.010074 |
| ENSMUSG00000090063 | 0 | 0 | -3.31689 | 0.003805 |
| ENSMUSG00000090084 | 0 | 0 | 1.809576 | 0.113955 |
| ENSMUSG00000090394 | 0 | 0 | 2.31078 | 0.047791 |
| ENSMUSG00000090698 | 0 | 0 | -5.5146 | 0.007122 |
| ENSMUSG00000090841 | 0 | 0 | 1.612903 | 0.013676 |
| ENSMUSG00000091050 | 0 | 0 | -2.96068 | 0.680962 |
| ENSMUSG00000091243 | 0 | 0 | 1.971225 | 0.105958 |
| ENSMUSG00000091337 | 0 | 0 | -1.88338 | 0.081988 |
| ENSMUSG00000091562 | 0 | 0 | -1.52572 | 0.048242 |
| ENSMUSG00000091898 | 0 | 0 | 2.692646 | 0.928367 |
| ENSMUSG00000091971 | 0 | 0 | -1.59307 | 0.514381 |
| ENSMUSG00000092274 | 0 | 0 | 2.912173 | 0.006649 |
| ENSMUSG00000092572 | 0 | 0 | 2.291344 | 0.306526 |
| ENSMUSG00000093565 | 0 | 0 | -1.86891 | 0.239138 |
| ENSMUSG00000093738 | 0 | 0 | -1.68237 | 0.027632 |
| ENSMUSG00000093989 | 0 | 0 | 2.109734 | 0.221748 |
| ENSMUSG00000094103 | 0 | 0 | 3.264859 | 0.022191 |
| ENSMUSG00000094910 | 0 | 0 | -3.28175 | 0.122397 |
| ENSMUSG00000095595 | 0 | 0 | 1.50378 | 0.068288 |
| ENSMUSG00000096010 | 0 | 0 | -1.6348 | 0.132203 |
| ENSMUSG00000096054 | 0 | 0 | 2.279026 | 0.012438 |
| ENSMUSG00000096768 | 0 | 0 | -1.68606 | 0.13066 |
| ENSMUSG00000096847 | 0 | 0 | -3.39869 | 0.497933 |
| ENSMUSG00000096965 | 0 | 0 | 8.805869 | 0.821204 |
| ENSMUSG00000097039 | 0 | 0 | 2.022268 | 0.145746 |
| ENSMUSG00000097040 | 0 | 0 | -2.18999 | 0.619545 |
| ENSMUSG00000097048 | 0 | 0 | -1.93298 | 0.199733 |
| ENSMUSG00000097099 | 0 | 0 | -1.5595 | 0.088363 |
| ENSMUSG00000097142 | 0 | 0 | -1.53875 | 0.168485 |
| ENSMUSG00000097145 | 0 | 0 | 3.235775 | 0.161226 |
| ENSMUSG00000097252 | 0 | 0 | 7.513154 | 2.640464 |
| ENSMUSG00000097312 | 0 | 0 | -2.39011 | 0.307877 |
| ENSMUSG00000097324 | 0 | 0 | 3.850907 | 0.087827 |
| ENSMUSG00000097336 | 0 | 0 | -3.62663 | 0.137532 |
| ENSMUSG00000097347 | 0 | 0 | -1.9061 | 0.09355 |
| ENSMUSG00000097487 | 0 | 0 | 2.360548 | 0.066326 |
| ENSMUSG00000097554 | 0 | 0 | -2.51786 | 0.064183 |
| ENSMUSG00000097616 | 0 | 0 | 1.503539 | 0.178895 |
| ENSMUSG00000097695 | 0 | 0 | -2.08442 | 0.190769 |
| ENSMUSG00000097789 | 0 | 0 | 2.152984 | 0.02415 |
| ENSMUSG00000097810 | 0 | 0 | 2.181316 | 0.158609 |
| ENSMUSG00000097993 | 0 | 0 | 2.322729 | 0.045978 |
| ENSMUSG00000098509 | 0 | 0 | 5.852077 | 0.071426 |
| ENSMUSG00000098557 | 0 | 0 | -1.97816 | 0.234634 |
| ENSMUSG00000099937 | 0 | 0 | -1.81888 | 0.171704 |
| ENSMUSG00000100750 | 0 | 0 | -2.7381 | 0.126408 |
| ENSMUSG00000101698 | 0 | 0 | -2.26918 | 0.112549 |
