# Supplementary material for: Insight into the maintenance of odontogenic potential in mouse dental mesenchymal cells based on transcriptomic analysis
Source: PeerJ. 2016 Feb 22;4:e1684. doi: 10.7717/peerj.1684 (PMC4768683; doi:10.7717/peerj.1684)
Supplement: Data S2 [file peerj-04-1684-s002.doc]

| ensg | mean_P0 | err_P0 | mean_P2 | err_P2 |
| --- | --- | --- | --- | --- |
| ENSMUSG00000001349 | 0 | 0 | 11.44965 | 0.131538 |
| ENSMUSG00000072812 | 0 | 0 | 9.87522 | 0.24673 |
| ENSMUSG00000059430 | 0 | 0 | 9.360865 | 0.376325 |
| ENSMUSG00000096965 | 0 | 0 | 9.344734 | 1.156912 |
| ENSMUSG00000032715 | 0 | 0 | 9.152803 | 0.182934 |
| ENSMUSG00000027656 | 0 | 0 | 8.980876 | 0.083848 |
| ENSMUSG00000042115 | 0 | 0 | 8.818644 | 0.197693 |
| ENSMUSG00000021950 | 0 | 0 | 8.664312 | 0.419166 |
| ENSMUSG00000073802 | 0 | 0 | 8.583257 | 0.242284 |
| ENSMUSG00000028108 | 0 | 0 | 8.557997 | 1.182299 |
| ENSMUSG00000030468 | 0 | 0 | 8.407076 | 2.230027 |
| ENSMUSG00000024803 | 0 | 0 | 8.276201 | 2.478811 |
| ENSMUSG00000043822 | 0 | 0 | 8.163709 | 0.491657 |
| ENSMUSG00000035783 | 0 | 0 | 8.150702 | 0.245678 |
| ENSMUSG00000030717 | 0 | 0 | 8.135926 | 0.716573 |
| ENSMUSG00000047878 | 0 | 0 | 7.962726 | 1.715701 |
| ENSMUSG00000040329 | 0 | 0 | 7.683144 | 2.376279 |
| ENSMUSG00000097252 | 0 | 0 | 7.63889 | 2.504318 |
| ENSMUSG00000017817 | 0 | 0 | 7.566687 | 0.452694 |
| ENSMUSG00000048096 | 0 | 0 | 7.534146 | 0.341359 |
| ENSMUSG00000052353 | 0 | 0 | 7.404973 | 0.258849 |
| ENSMUSG00000027335 | 0 | 0 | 7.290963 | 0.131471 |
| ENSMUSG00000024883 | 0 | 0 | 7.289946 | 0.109924 |
| ENSMUSG00000075602 | 0 | 0 | 7.1531 | 0.442258 |
| ENSMUSG00000021798 | 0 | 0 | 7.101536 | 0.626213 |
| ENSMUSG00000063727 | 0 | 0 | 7.087365 | 0.096112 |
| ENSMUSG00000023046 | 0 | 0 | 7.07864 | 0.506018 |
| ENSMUSG00000038776 | 0 | 0 | 7.029836 | 0.124872 |
| ENSMUSG00000042759 | 0 | 0 | 6.884114 | 0.159737 |
| ENSMUSG00000040133 | 0 | 0 | 6.839288 | 0.295316 |
| ENSMUSG00000039813 | 0 | 0 | 6.816105 | 0.104284 |
| ENSMUSG00000022146 | 0 | 0 | 6.737744 | 0.089734 |
| ENSMUSG00000002020 | 0 | 0 | 6.683812 | 0.027252 |
| ENSMUSG00000030116 | 0 | 0 | 6.632364 | 0.487899 |
| ENSMUSG00000025586 | 0 | 0 | 6.602674 | 0.009997 |
| ENSMUSG00000041957 | 0 | 0 | 6.538747 | 0.29954 |
| ENSMUSG00000055407 | 0 | 0 | 6.48257 | 0.046837 |
| ENSMUSG00000032060 | 0 | 0 | 6.424092 | 0.370905 |
| ENSMUSG00000036067 | 0 | 0 | 6.348609 | 0.487938 |
| ENSMUSG00000026308 | 0 | 0 | 6.314831 | 0.623693 |
| ENSMUSG00000074813 | 0 | 0 | 6.313851 | 0.265434 |
| ENSMUSG00000074934 | 0 | 0 | 6.298717 | 0.021575 |
| ENSMUSG00000098509 | 0 | 0 | 6.294156 | 0.061865 |
| ENSMUSG00000032085 | 0 | 0 | 6.271681 | 0.183144 |
| ENSMUSG00000028042 | 0 | 0 | 6.261485 | 0.043277 |
| ENSMUSG00000027313 | 0 | 0 | 6.241749 | 0.19437 |
| ENSMUSG00000052316 | 0 | 0 | 6.18067 | 0.082539 |
| ENSMUSG00000035373 | 0 | 0 | 6.141383 | 0.666231 |
| ENSMUSG00000029816 | 0 | 0 | 6.1398 | 0.203822 |
| ENSMUSG00000034394 | 0 | 0 | 6.037291 | 0.809961 |
| ENSMUSG00000020411 | 0 | 0 | 6.008103 | 0.172773 |
| ENSMUSG00000023067 | 0 | 0 | 5.977791 | 0.174451 |
| ENSMUSG00000028776 | 0 | 0 | 5.952216 | 0.524938 |
| ENSMUSG00000076441 | 0 | 0 | 5.903452 | 0.148584 |
| ENSMUSG00000020256 | 0 | 0 | 5.830172 | 0.0888 |
| ENSMUSG00000042429 | 0 | 0 | 5.788733 | 0.430839 |
| ENSMUSG00000022758 | 0 | 0 | 5.760323 | 0.878755 |
| ENSMUSG00000045838 | 0 | 0 | 5.732215 | 0.197859 |
| ENSMUSG00000026069 | 0 | 0 | 5.727 | 0.058283 |
| ENSMUSG00000048458 | 0 | 0 | 5.717348 | 0.363104 |
| ENSMUSG00000031070 | 0 | 0 | 5.6703 | 0.378379 |
| ENSMUSG00000019230 | 0 | 0 | 5.659983 | 0.158287 |
| ENSMUSG00000056596 | 0 | 0 | 5.638225 | 0.508049 |
| ENSMUSG00000028370 | 0 | 0 | 5.620209 | 0.030181 |
| ENSMUSG00000037362 | 0 | 0 | 5.613428 | 0.144455 |
| ENSMUSG00000048706 | 0 | 0 | 5.606341 | 0.040529 |
| ENSMUSG00000029061 | 0 | 0 | 5.603725 | 0.100629 |
| ENSMUSG00000030281 | 0 | 0 | 5.574022 | 0.123121 |
| ENSMUSG00000022861 | 0 | 0 | 5.558535 | 0.230825 |
| ENSMUSG00000037946 | 0 | 0 | 5.527311 | 0.554081 |
| ENSMUSG00000037035 | 0 | 0 | 5.508968 | 0.047411 |
| ENSMUSG00000030790 | 0 | 0 | 5.504823 | 0.083777 |
| ENSMUSG00000031762 | 0 | 0 | 5.470149 | 0.491008 |
| ENSMUSG00000046694 | 0 | 0 | 5.42075 | 0.23008 |
| ENSMUSG00000022665 | 0 | 0 | 5.395043 | 0.000469 |
| ENSMUSG00000028780 | 0 | 0 | 5.39189 | 0.037372 |
| ENSMUSG00000037411 | 0 | 0 | 5.37672 | 0.100353 |
| ENSMUSG00000025185 | 0 | 0 | 5.361083 | 0.059344 |
| ENSMUSG00000035385 | 0 | 0 | 5.350204 | 0.493851 |
| ENSMUSG00000018830 | 0 | 0 | 5.348255 | 0.964262 |
| ENSMUSG00000029377 | 0 | 0 | 5.298082 | 0.179769 |
| ENSMUSG00000069893 | 0 | 0 | 5.29352 | 0.323534 |
| ENSMUSG00000020154 | 0 | 0 | 5.292779 | 0.151256 |
| ENSMUSG00000033207 | 0 | 0 | 5.289014 | 0.191535 |
| ENSMUSG00000015850 | 0 | 0 | 5.28065 | 0.314434 |
| ENSMUSG00000055044 | 0 | 0 | 5.276876 | 0.175715 |
| ENSMUSG00000030862 | 0 | 0 | 5.263808 | 0.15091 |
| ENSMUSG00000004098 | 0 | 0 | 5.220833 | 0.067939 |
| ENSMUSG00000020303 | 0 | 0 | 5.220283 | 0.391516 |
| ENSMUSG00000066755 | 0 | 0 | 5.216346 | 0.631819 |
| ENSMUSG00000031026 | 0 | 0 | 5.188234 | 0.388922 |
| ENSMUSG00000033880 | 0 | 0 | 5.183761 | 0.424389 |
| ENSMUSG00000026904 | 0 | 0 | 5.170059 | 0.144643 |
| ENSMUSG00000031765 | 0 | 0 | 5.153558 | 0.62922 |
| ENSMUSG00000056888 | 0 | 0 | 5.113202 | 0.231542 |
| ENSMUSG00000025355 | 0 | 0 | 5.098408 | 0.277757 |
| ENSMUSG00000028773 | 0 | 0 | 5.069303 | 0.092918 |
| ENSMUSG00000031297 | 0 | 0 | 5.052528 | 0.094389 |
| ENSMUSG00000040666 | 0 | 0 | 5.050988 | 0.09281 |
| ENSMUSG00000079014 | 0 | 0 | 5.025184 | 0.213287 |
| ENSMUSG00000024521 | 0 | 0 | 4.998103 | 0.002768 |
| ENSMUSG00000040350 | 0 | 0 | 4.98481 | 0.253667 |
| ENSMUSG00000050335 | 0 | 0 | 4.98115 | 0.086918 |
| ENSMUSG00000005357 | 0 | 0 | 4.97436 | 0.347257 |
| ENSMUSG00000019851 | 0 | 0 | 4.956004 | 0.550264 |
| ENSMUSG00000053746 | 0 | 0 | 4.950339 | 0.061574 |
| ENSMUSG00000039450 | 0 | 0 | 4.939225 | 0.508456 |
| ENSMUSG00000068196 | 0 | 0 | 4.915099 | 0.028536 |
| ENSMUSG00000042190 | 0 | 0 | 4.893011 | 0.266645 |
| ENSMUSG00000018507 | 0 | 0 | 4.891242 | 0.385592 |
| ENSMUSG00000055301 | 0 | 0 | 4.882068 | 0.118828 |
| ENSMUSG00000022594 | 0 | 0 | 4.875234 | 0.10487 |
| ENSMUSG00000045954 | 0 | 0 | 4.80381 | 0.425042 |
| ENSMUSG00000032271 | 0 | 0 | 4.786051 | 0.17039 |
| ENSMUSG00000005611 | 0 | 0 | 4.769812 | 0.443252 |
| ENSMUSG00000001020 | 0 | 0 | 4.76275 | 0.184746 |
| ENSMUSG00000031253 | 0 | 0 | 4.751893 | 0.286038 |
| ENSMUSG00000063558 | 0 | 0 | 4.740721 | 0.072482 |
| ENSMUSG00000020057 | 0 | 0 | 4.712989 | 0.021828 |
| ENSMUSG00000028970 | 0 | 0 | 4.708096 | 0.564846 |
| ENSMUSG00000025875 | 0 | 0 | 4.707651 | 0.657509 |
| ENSMUSG00000028273 | 0 | 0 | 4.686631 | 0.084034 |
| ENSMUSG00000020424 | 0 | 0 | 4.679271 | 0.175184 |
| ENSMUSG00000020326 | 0 | 0 | 4.667195 | 0.189595 |
| ENSMUSG00000006205 | 0 | 0 | 4.582453 | 0.095573 |
| ENSMUSG00000024339 | 0 | 0 | 4.569039 | 0.179289 |
| ENSMUSG00000041073 | 0 | 0 | 4.56776 | 0.130072 |
| ENSMUSG00000048065 | 0 | 0 | 4.564043 | 0.323313 |
| ENSMUSG00000029161 | 0 | 0 | 4.55791 | 0.124931 |
| ENSMUSG00000074676 | 0 | 0 | 4.555076 | 0.542664 |
| ENSMUSG00000047443 | 0 | 0 | 4.540569 | 0.332317 |
| ENSMUSG00000027171 | 0 | 0 | 4.520287 | 0.391496 |
| ENSMUSG00000060240 | 0 | 0 | 4.509731 | 0.322279 |
| ENSMUSG00000073274 | 0 | 0 | 4.505009 | 0.365056 |
| ENSMUSG00000046814 | 0 | 0 | 4.503675 | 0.190455 |
| ENSMUSG00000007655 | 0 | 0 | 4.493817 | 0.240666 |
| ENSMUSG00000059555 | 0 | 0 | 4.490428 | 0.098747 |
| ENSMUSG00000053469 | 0 | 0 | 4.48579 | 0.279286 |
| ENSMUSG00000032899 | 0 | 0 | 4.480784 | 0.334753 |
| ENSMUSG00000028128 | 0 | 0 | 4.476803 | 0.184267 |
| ENSMUSG00000034275 | 0 | 0 | 4.475287 | 0.468689 |
| ENSMUSG00000039058 | 0 | 0 | 4.427683 | 0.077066 |
| ENSMUSG00000034457 | 0 | 0 | 4.422686 | 0.215257 |
| ENSMUSG00000050777 | 0 | 0 | 4.417925 | 0.366939 |
| ENSMUSG00000024529 | 0 | 0 | 4.38819 | 0.061567 |
| ENSMUSG00000040152 | 0 | 0 | 4.367566 | 0.012164 |
| ENSMUSG00000022894 | 0 | 0 | 4.365154 | 0.224095 |
| ENSMUSG00000028259 | 0 | 0 | 4.363132 | 0.138812 |
| ENSMUSG00000049872 | 0 | 0 | 4.359742 | 0.265539 |
| ENSMUSG00000030607 | 0 | 0 | 4.350222 | 0.501485 |
| ENSMUSG00000026204 | 0 | 0 | 4.343599 | 0.499583 |
| ENSMUSG00000017002 | 0 | 0 | 4.338536 | 0.061454 |
| ENSMUSG00000010154 | 0 | 0 | 4.326919 | 0.416216 |
| ENSMUSG00000038742 | 0 | 0 | 4.322964 | 0.289183 |
| ENSMUSG00000031595 | 0 | 0 | 4.30342 | 0.355488 |
| ENSMUSG00000026430 | 0 | 0 | 4.275539 | 0.258422 |
| ENSMUSG00000023249 | 0 | 0 | 4.269794 | 0.187285 |
| ENSMUSG00000046807 | 0 | 0 | 4.264016 | 0.009618 |
| ENSMUSG00000051726 | 0 | 0 | 4.262734 | 0.389375 |
| ENSMUSG00000041559 | 0 | 0 | 4.26245 | 0.128636 |
| ENSMUSG00000029484 | 0 | 0 | 4.244628 | 0.31957 |
| ENSMUSG00000021678 | 0 | 0 | 4.235505 | 0.514709 |
| ENSMUSG00000044254 | 0 | 0 | 4.225766 | 0.27382 |
| ENSMUSG00000079484 | 0 | 0 | 4.225721 | 0.657024 |
| ENSMUSG00000040714 | 0 | 0 | 4.213525 | 0.042795 |
| ENSMUSG00000039126 | 0 | 0 | 4.212978 | 0.045707 |
| ENSMUSG00000063268 | 0 | 0 | 4.209221 | 0.323135 |
| ENSMUSG00000034591 | 0 | 0 | 4.20413 | 0.005826 |
| ENSMUSG00000022887 | 0 | 0 | 4.166173 | 0.079273 |
| ENSMUSG00000004951 | 0 | 0 | 4.16292 | 0.460876 |
| ENSMUSG00000044042 | 0 | 0 | 4.151199 | 0.145983 |
| ENSMUSG00000040612 | 0 | 0 | 4.149657 | 0.47821 |
| ENSMUSG00000031972 | 0 | 0 | 4.147423 | 0.24476 |
| ENSMUSG00000012519 | 0 | 0 | 4.108849 | 0.234092 |
| ENSMUSG00000068758 | 0 | 0 | 4.108656 | 0.176303 |
| ENSMUSG00000031778 | 0 | 0 | 4.079212 | 0.866284 |
| ENSMUSG00000039103 | 0 | 0 | 4.076223 | 0.103886 |
| ENSMUSG00000078922 | 0 | 0 | 4.07309 | 0.189711 |
| ENSMUSG00000025348 | 0 | 0 | 4.056979 | 0.229025 |
| ENSMUSG00000063506 | 0 | 0 | 4.055582 | 0.114612 |
| ENSMUSG00000049511 | 0 | 0 | 4.050677 | 0.267918 |
| ENSMUSG00000036136 | 0 | 0 | 4.050071 | 0.14123 |
| ENSMUSG00000097324 | 0 | 0 | 4.030447 | 0.772052 |
| ENSMUSG00000055401 | 0 | 0 | 4.007081 | 0.021395 |
| ENSMUSG00000027188 | 0 | 0 | 3.975585 | 0.065514 |
| ENSMUSG00000020019 | 0 | 0 | 3.972373 | 0.069458 |
| ENSMUSG00000035279 | 0 | 0 | 3.967419 | 0.080429 |
| ENSMUSG00000025492 | 0 | 0 | 3.9567 | 0.355456 |
| ENSMUSG00000026817 | 0 | 0 | 3.944495 | 0.126877 |
| ENSMUSG00000049404 | 0 | 0 | 3.937115 | 0.282654 |
| ENSMUSG00000024659 | 0 | 0 | 3.927981 | 0.060568 |
| ENSMUSG00000085148 | 0 | 0 | 3.914803 | 0.205752 |
| ENSMUSG00000028539 | 0 | 0 | 3.911205 | 0.653196 |
| ENSMUSG00000048612 | 0 | 0 | 3.8975 | 0.051975 |
| ENSMUSG00000028270 | 0 | 0 | 3.892808 | 0.38231 |
| ENSMUSG00000060519 | 0 | 0 | 3.886899 | 0.137829 |
| ENSMUSG00000021281 | 0 | 0 | 3.88534 | 0.045461 |
| ENSMUSG00000045679 | 0 | 0 | 3.872483 | 0.272071 |
| ENSMUSG00000030623 | 0 | 0 | 3.859149 | 0.260823 |
| ENSMUSG00000059456 | 0 | 0 | 3.835632 | 0.533523 |
| ENSMUSG00000034040 | 0 | 0 | 3.829555 | 0.159423 |
| ENSMUSG00000046761 | 0 | 0 | 3.828535 | 0.286941 |
| ENSMUSG00000001333 | 0 | 0 | 3.822624 | 0.220616 |
| ENSMUSG00000009687 | 0 | 0 | 3.809239 | 0.064851 |
| ENSMUSG00000031289 | 0 | 0 | 3.799567 | 0.554724 |
| ENSMUSG00000036599 | 0 | 0 | 3.796447 | 0.027975 |
| ENSMUSG00000057137 | 0 | 0 | 3.79514 | 0.175909 |
| ENSMUSG00000027955 | 0 | 0 | 3.793061 | 0.292519 |
| ENSMUSG00000026768 | 0 | 0 | 3.789624 | 0.162426 |
| ENSMUSG00000041481 | 0 | 0 | 3.784801 | 0.081533 |
| ENSMUSG00000030681 | 0 | 0 | 3.764708 | 0.041639 |
| ENSMUSG00000019124 | 0 | 0 | 3.758483 | 0.074753 |
| ENSMUSG00000020758 | 0 | 0 | 3.749626 | 0.518376 |
| ENSMUSG00000036766 | 0 | 0 | 3.746692 | 0.391454 |
| ENSMUSG00000046223 | 0 | 0 | 3.741862 | 0.309265 |
| ENSMUSG00000020325 | 0 | 0 | 3.720451 | 0.028106 |
| ENSMUSG00000035595 | 0 | 0 | 3.71404 | 0.057414 |
| ENSMUSG00000086843 | 0 | 0 | 3.702114 | 0.115454 |
| ENSMUSG00000024937 | 0 | 0 | 3.693507 | 0.230254 |
| ENSMUSG00000001025 | 0 | 0 | 3.684309 | 0.081479 |
| ENSMUSG00000024805 | 0 | 0 | 3.68178 | 0.042258 |
| ENSMUSG00000026536 | 0 | 0 | 3.676016 | 0.143962 |
| ENSMUSG00000046718 | 0 | 0 | 3.669569 | 0.018472 |
| ENSMUSG00000046186 | 0 | 0 | 3.666101 | 0.182761 |
| ENSMUSG00000027832 | 0 | 0 | 3.661328 | 0.262995 |
| ENSMUSG00000039347 | 0 | 0 | 3.659626 | 0.372843 |
| ENSMUSG00000030284 | 0 | 0 | 3.651841 | 0.293991 |
| ENSMUSG00000040296 | 0 | 0 | 3.636275 | 0.117435 |
| ENSMUSG00000033386 | 0 | 0 | 3.632084 | 0.0638 |
| ENSMUSG00000053279 | 0 | 0 | 3.599623 | 0.310294 |
| ENSMUSG00000018593 | 0 | 0 | 3.588383 | 0 |
| ENSMUSG00000001131 | 0 | 0 | 3.565152 | 0.159149 |
| ENSMUSG00000036006 | 0 | 0 | 3.560262 | 0.453806 |
| ENSMUSG00000005124 | 0 | 0 | 3.560148 | 0.092639 |
| ENSMUSG00000067001 | 0 | 0 | 3.557233 | 0.197916 |
| ENSMUSG00000039005 | 0 | 0 | 3.55431 | 0.205469 |
| ENSMUSG00000019997 | 0 | 0 | 3.551669 | 0.188818 |
| ENSMUSG00000072674 | 0 | 0 | 3.530489 | 0.215034 |
| ENSMUSG00000032011 | 0 | 0 | 3.516704 | 0.634031 |
| ENSMUSG00000092274 | 0 | 0 | 3.515569 | 0.208319 |
| ENSMUSG00000042349 | 0 | 0 | 3.51132 | 0.013878 |
| ENSMUSG00000034981 | 0 | 0 | 3.510873 | 0.59198 |
| ENSMUSG00000028744 | 0 | 0 | 3.510824 | 0.240216 |
| ENSMUSG00000038539 | 0 | 0 | 3.509583 | 0.010204 |
| ENSMUSG00000021876 | 0 | 0 | 3.506842 | 0.069847 |
| ENSMUSG00000032418 | 0 | 0 | 3.498809 | 0.215311 |
| ENSMUSG00000046312 | 0 | 0 | 3.482981 | 0.068055 |
| ENSMUSG00000015652 | 0 | 0 | 3.472643 | 0.205086 |
| ENSMUSG00000041548 | 0 | 0 | 3.468007 | 0.161564 |
| ENSMUSG00000043613 | 0 | 0 | 3.457453 | 0.528321 |
| ENSMUSG00000031373 | 0 | 0 | 3.455317 | 0.005795 |
| ENSMUSG00000030782 | 0 | 0 | 3.443493 | 0.215224 |
| ENSMUSG00000032366 | 0 | 0 | 3.44069 | 0.025161 |
| ENSMUSG00000020614 | 0 | 0 | 3.440667 | 0.322597 |
| ENSMUSG00000041577 | 0 | 0 | 3.415508 | 0.226364 |
| ENSMUSG00000037594 | 0 | 0 | 3.40748 | 0.010672 |
| ENSMUSG00000094103 | 0 | 0 | 3.391006 | 0.221639 |
| ENSMUSG00000026956 | 0 | 0 | 3.389269 | 0.032751 |
| ENSMUSG00000063873 | 0 | 0 | 3.386146 | 0.06187 |
| ENSMUSG00000041736 | 0 | 0 | 3.383074 | 0.132165 |
| ENSMUSG00000035873 | 0 | 0 | 3.381755 | 0.084475 |
| ENSMUSG00000062345 | 0 | 0 | 3.366804 | 0.045572 |
| ENSMUSG00000021185 | 0 | 0 | 3.345466 | 0.021258 |
| ENSMUSG00000017734 | 0 | 0 | 3.341103 | 0.340226 |
| ENSMUSG00000030409 | 0 | 0 | 3.333061 | 0.41935 |
| ENSMUSG00000026142 | 0 | 0 | 3.331642 | 0.173479 |
| ENSMUSG00000038387 | 0 | 0 | 3.329866 | 0.108266 |
| ENSMUSG00000019278 | 0 | 0 | 3.326068 | 0.03332 |
| ENSMUSG00000009772 | 0 | 0 | 3.32599 | 0.145842 |
| ENSMUSG00000027956 | 0 | 0 | 3.317972 | 0.118287 |
| ENSMUSG00000024778 | 0 | 0 | 3.313091 | 0.346632 |
| ENSMUSG00000030268 | 0 | 0 | 3.307231 | 0.090508 |
| ENSMUSG00000053647 | 0 | 0 | 3.296751 | 0.510361 |
| ENSMUSG00000036256 | 0 | 0 | 3.295935 | 0.143307 |
| ENSMUSG00000079592 | 0 | 0 | 3.288196 | 0.374078 |
| ENSMUSG00000033467 | 0 | 0 | 3.266105 | 0.241829 |
| ENSMUSG00000029381 | 0 | 0 | 3.265247 | 0.040643 |
| ENSMUSG00000039323 | 0 | 0 | 3.261072 | 0.002428 |
| ENSMUSG00000000058 | 0 | 0 | 3.258176 | 0.092539 |
| ENSMUSG00000021196 | 0 | 0 | 3.241967 | 0.04829 |
| ENSMUSG00000008136 | 0 | 0 | 3.239501 | 0.132745 |
| ENSMUSG00000019194 | 0 | 0 | 3.23354 | 0.069304 |
| ENSMUSG00000033306 | 0 | 0 | 3.233161 | 0.441544 |
| ENSMUSG00000036564 | 0 | 0 | 3.229581 | 0.019773 |
| ENSMUSG00000030306 | 0 | 0 | 3.223472 | 0.568831 |
| ENSMUSG00000034361 | 0 | 0 | 3.219521 | 0.027398 |
| ENSMUSG00000060477 | 0 | 0 | 3.208899 | 0.101783 |
| ENSMUSG00000027858 | 0 | 0 | 3.208097 | 0.165859 |
| ENSMUSG00000027800 | 0 | 0 | 3.200855 | 0.548626 |
| ENSMUSG00000054293 | 0 | 0 | 3.197636 | 0.103374 |
| ENSMUSG00000053615 | 0 | 0 | 3.192003 | 0.087708 |
| ENSMUSG00000070469 | 0 | 0 | 3.190961 | 0.329175 |
| ENSMUSG00000030089 | 0 | 0 | 3.18554 | 0.134378 |
| ENSMUSG00000026321 | 0 | 0 | 3.183879 | 0.140762 |
| ENSMUSG00000010358 | 0 | 0 | 3.168907 | 0.012346 |
| ENSMUSG00000026389 | 0 | 0 | 3.166956 | 0.321195 |
| ENSMUSG00000028464 | 0 | 0 | 3.160756 | 0.16445 |
| ENSMUSG00000029119 | 0 | 0 | 3.160209 | 0.2527 |
| ENSMUSG00000079355 | 0 | 0 | 3.155759 | 0.04363 |
| ENSMUSG00000028919 | 0 | 0 | 3.155291 | 0.07818 |
| ENSMUSG00000062753 | 0 | 0 | 3.151862 | 0.18225 |
| ENSMUSG00000091898 | 0 | 0 | 3.141478 | 0.931361 |
| ENSMUSG00000026885 | 0 | 0 | 3.141277 | 0.183829 |
| ENSMUSG00000019080 | 0 | 0 | 3.141202 | 0.34569 |
| ENSMUSG00000024851 | 0 | 0 | 3.138044 | 0.038683 |
| ENSMUSG00000024664 | 0 | 0 | 3.136322 | 0.247221 |
| ENSMUSG00000028369 | 0 | 0 | 3.132676 | 0.05462 |
| ENSMUSG00000021186 | 0 | 0 | 3.130005 | 0.045166 |
| ENSMUSG00000027663 | 0 | 0 | 3.125196 | 0.250219 |
| ENSMUSG00000021759 | 0 | 0 | 3.11791 | 0.030518 |
| ENSMUSG00000036745 | 0 | 0 | 3.10985 | 0.31149 |
| ENSMUSG00000061758 | 0 | 0 | 3.107934 | 0.179631 |
| ENSMUSG00000021367 | 0 | 0 | 3.107891 | 0.43735 |
| ENSMUSG00000068335 | 0 | 0 | 3.106385 | 0.047312 |
| ENSMUSG00000024725 | 0 | 0 | 3.1043 | 0.051014 |
| ENSMUSG00000035559 | 0 | 0 | 3.102641 | 0.053713 |
| ENSMUSG00000042215 | 0 | 0 | 3.100531 | 0.126777 |
| ENSMUSG00000023092 | 0 | 0 | 3.098191 | 0.289036 |
| ENSMUSG00000037990 | 0 | 0 | 3.093791 | 0.043686 |
| ENSMUSG00000073489 | 0 | 0 | 3.093486 | 0.127374 |
| ENSMUSG00000000359 | 0 | 0 | 3.091439 | 0.291433 |
| ENSMUSG00000026589 | 0 | 0 | 3.089659 | 0.311111 |
| ENSMUSG00000073771 | 0 | 0 | 3.082504 | 0.105612 |
| ENSMUSG00000032719 | 0 | 0 | 3.079195 | 0.006559 |
| ENSMUSG00000041801 | 0 | 0 | 3.065264 | 0.162462 |
| ENSMUSG00000031176 | 0 | 0 | 3.062724 | 0.010414 |
| ENSMUSG00000042766 | 0 | 0 | 3.058017 | 0.034169 |
| ENSMUSG00000054951 | 0 | 0 | 3.047077 | 0.362512 |
| ENSMUSG00000097145 | 0 | 0 | 3.04305 | 0.194142 |
| ENSMUSG00000029826 | 0 | 0 | 3.041541 | 0.180757 |
| ENSMUSG00000036412 | 0 | 0 | 3.039782 | 0.12051 |
| ENSMUSG00000027500 | 0 | 0 | 3.036137 | 0.323935 |
| ENSMUSG00000024049 | 0 | 0 | 3.034778 | 0.511209 |
| ENSMUSG00000005803 | 0 | 0 | 3.033394 | 0.166903 |
| ENSMUSG00000025887 | 0 | 0 | 3.028316 | 0.056909 |
| ENSMUSG00000060675 | 0 | 0 | 3.028222 | 0.38501 |
| ENSMUSG00000038932 | 0 | 0 | 3.027834 | 0.02561 |
| ENSMUSG00000041842 | 0 | 0 | 3.021122 | 0.392962 |
| ENSMUSG00000025937 | 0 | 0 | 3.020101 | 0.126651 |
| ENSMUSG00000086070 | 0 | 0 | 3.000825 | 0.277308 |
| ENSMUSG00000054555 | 0 | 0 | 2.997744 | 0.013593 |
| ENSMUSG00000033717 | 0 | 0 | 2.990096 | 0.063243 |
| ENSMUSG00000066952 | 0 | 0 | 2.988601 | 0.478176 |
| ENSMUSG00000015647 | 0 | 0 | 2.98776 | 0.195213 |
| ENSMUSG00000026564 | 0 | 0 | 2.984057 | 0.032144 |
| ENSMUSG00000022206 | 0 | 0 | 2.982844 | 0.269969 |
| ENSMUSG00000054580 | 0 | 0 | 2.980953 | 0.071572 |
| ENSMUSG00000039745 | 0 | 0 | 2.970059 | 0.056228 |
| ENSMUSG00000020806 | 0 | 0 | 2.962132 | 0.030707 |
| ENSMUSG00000033149 | 0 | 0 | 2.955119 | 0.039186 |
| ENSMUSG00000031465 | 0 | 0 | 2.949589 | 1.009737 |
| ENSMUSG00000074968 | 0 | 0 | 2.9333 | 0.130381 |
| ENSMUSG00000034177 | 0 | 0 | 2.93167 | 0.095156 |
| ENSMUSG00000034161 | 0 | 0 | 2.919832 | 0.22594 |
| ENSMUSG00000032026 | 0 | 0 | 2.919319 | 0.052419 |
| ENSMUSG00000090394 | 0 | 0 | 2.916886 | 0.126822 |
| ENSMUSG00000045980 | 0 | 0 | 2.876576 | 0.298435 |
| ENSMUSG00000029304 | 0 | 0 | 2.875057 | 0.373493 |
| ENSMUSG00000026932 | 0 | 0 | 2.870025 | 0.004817 |
| ENSMUSG00000027254 | 0 | 0 | 2.865531 | 0.029551 |
| ENSMUSG00000015335 | 0 | 0 | 2.863972 | 0.049527 |
| ENSMUSG00000060600 | 0 | 0 | 2.859393 | 0.517447 |
| ENSMUSG00000023905 | 0 | 0 | 2.858067 | 0.220302 |
| ENSMUSG00000016918 | 0 | 0 | 2.856421 | 0.144222 |
| ENSMUSG00000031626 | 0 | 0 | 2.855942 | 0.415097 |
| ENSMUSG00000001506 | 0 | 0 | 2.850766 | 0.357362 |
| ENSMUSG00000039405 | 0 | 0 | 2.846433 | 0.325711 |
| ENSMUSG00000040618 | 0 | 0 | 2.840594 | 0.095686 |
| ENSMUSG00000032487 | 0 | 0 | 2.835297 | 0.427326 |
| ENSMUSG00000005413 | 0 | 0 | 2.832958 | 0.188921 |
| ENSMUSG00000001123 | 0 | 0 | 2.827076 | 0.418485 |
| ENSMUSG00000030616 | 0 | 0 | 2.826192 | 0.047105 |
| ENSMUSG00000079499 | 0 | 0 | 2.824387 | 0.040246 |
| ENSMUSG00000014599 | 0 | 0 | 2.820788 | 0.006525 |
| ENSMUSG00000026456 | 0 | 0 | 2.819671 | 0.049258 |
| ENSMUSG00000061878 | 0 | 0 | 2.818204 | 0.119711 |
| ENSMUSG00000030894 | 0 | 0 | 2.814761 | 0.066225 |
| ENSMUSG00000067818 | 0 | 0 | 2.814629 | 0.060521 |
| ENSMUSG00000068566 | 0 | 0 | 2.810067 | 0.195587 |
| ENSMUSG00000051236 | 0 | 0 | 2.807152 | 0.003524 |
| ENSMUSG00000021866 | 0 | 0 | 2.783512 | 0.171946 |
| ENSMUSG00000038400 | 0 | 0 | 2.778503 | 0.05298 |
| ENSMUSG00000078920 | 0 | 0 | 2.77274 | 0.216937 |
| ENSMUSG00000047747 | 0 | 0 | 2.768947 | 0.189493 |
| ENSMUSG00000015133 | 0 | 0 | 2.761484 | 0.055514 |
| ENSMUSG00000049281 | 0 | 0 | 2.75635 | 0.704201 |
| ENSMUSG00000015980 | 0 | 0 | 2.753332 | 0.007406 |
| ENSMUSG00000029659 | 0 | 0 | 2.750981 | 0.301053 |
| ENSMUSG00000033576 | 0 | 0 | 2.746023 | 0.25525 |
| ENSMUSG00000021822 | 0 | 0 | 2.744377 | 0.006351 |
| ENSMUSG00000053626 | 0 | 0 | 2.736617 | 0.024706 |
| ENSMUSG00000055912 | 0 | 0 | 2.730902 | 0.327792 |
| ENSMUSG00000032849 | 0 | 0 | 2.73024 | 0.104191 |
| ENSMUSG00000054509 | 0 | 0 | 2.730218 | 0.238373 |
| ENSMUSG00000029671 | 0 | 0 | 2.725669 | 0.115234 |
| ENSMUSG00000001751 | 0 | 0 | 2.724831 | 0.121601 |
| ENSMUSG00000046768 | 0 | 0 | 2.720174 | 0.260057 |
| ENSMUSG00000025509 | 0 | 0 | 2.712809 | 0.00375 |
| ENSMUSG00000026202 | 0 | 0 | 2.70915 | 0.263019 |
| ENSMUSG00000027208 | 0 | 0 | 2.708911 | 0.060832 |
| ENSMUSG00000030522 | 0 | 0 | 2.703171 | 0.313257 |
| ENSMUSG00000029752 | 0 | 0 | 2.703028 | 0.00672 |
| ENSMUSG00000021009 | 0 | 0 | 2.697968 | 0.215931 |
| ENSMUSG00000040219 | 0 | 0 | 2.696903 | 0.07124 |
| ENSMUSG00000025085 | 0 | 0 | 2.694454 | 0.15358 |
| ENSMUSG00000031451 | 0 | 0 | 2.692393 | 0.003105 |
| ENSMUSG00000041607 | 0 | 0 | 2.691649 | 0.210374 |
| ENSMUSG00000031521 | 0 | 0 | 2.691019 | 0.241195 |
| ENSMUSG00000041625 | 0 | 0 | 2.682941 | 0.038678 |
| ENSMUSG00000039474 | 0 | 0 | 2.677814 | 0.269261 |
| ENSMUSG00000087141 | 0 | 0 | 2.675257 | 0.212231 |
| ENSMUSG00000001507 | 0 | 0 | 2.668795 | 0.162834 |
| ENSMUSG00000006301 | 0 | 0 | 2.661649 | 0.125375 |
| ENSMUSG00000042256 | 0 | 0 | 2.661001 | 0.139003 |
| ENSMUSG00000026786 | 0 | 0 | 2.655345 | 0.256284 |
| ENSMUSG00000021823 | 0 | 0 | 2.654875 | 0.082212 |
| ENSMUSG00000024376 | 0 | 0 | 2.650583 | 0.229346 |
| ENSMUSG00000033174 | 0 | 0 | 2.649251 | 0.003547 |
| ENSMUSG00000022090 | 0 | 0 | 2.646733 | 0.211641 |
| ENSMUSG00000030772 | 0 | 0 | 2.644388 | 0.070174 |
| ENSMUSG00000003746 | 0 | 0 | 2.643075 | 0.055434 |
| ENSMUSG00000035441 | 0 | 0 | 2.640065 | 0.215828 |
| ENSMUSG00000046876 | 0 | 0 | 2.638147 | 0.138545 |
| ENSMUSG00000051339 | 0 | 0 | 2.634212 | 0.039167 |
| ENSMUSG00000001665 | 0 | 0 | 2.629889 | 0.2416 |
| ENSMUSG00000033707 | 0 | 0 | 2.627898 | 0.056954 |
| ENSMUSG00000024302 | 0 | 0 | 2.623887 | 0.275413 |
| ENSMUSG00000022443 | 0 | 0 | 2.623449 | 0.102715 |
| ENSMUSG00000023904 | 0 | 0 | 2.619455 | 0.035272 |
| ENSMUSG00000029576 | 0 | 0 | 2.618442 | 0.213059 |
| ENSMUSG00000014602 | 0 | 0 | 2.613508 | 0.16283 |
| ENSMUSG00000074743 | 0 | 0 | 2.612221 | 0.047499 |
| ENSMUSG00000080115 | 0 | 0 | 2.611814 | 0.355308 |
| ENSMUSG00000096054 | 0 | 0 | 2.60833 | 0.247666 |
| ENSMUSG00000037814 | 0 | 0 | 2.604507 | 0.069139 |
| ENSMUSG00000097242 | 0 | 0 | 2.600765 | 0.318593 |
| ENSMUSG00000025006 | 0 | 0 | 2.593517 | 0.246379 |
| ENSMUSG00000021458 | 0 | 0 | 2.590824 | 0.394229 |
| ENSMUSG00000032006 | 0 | 0 | 2.589583 | 0.198744 |
| ENSMUSG00000063275 | 0 | 0 | 2.588451 | 0.140504 |
| ENSMUSG00000022575 | 0 | 0 | 2.588098 | 0.157461 |
| ENSMUSG00000047250 | 0 | 0 | 2.587107 | 0.268955 |
| ENSMUSG00000046879 | 0 | 0 | 2.583101 | 0.028782 |
| ENSMUSG00000038280 | 0 | 0 | 2.57257 | 0.029546 |
| ENSMUSG00000027574 | 0 | 0 | 2.57017 | 0.178926 |
| ENSMUSG00000097487 | 0 | 0 | 2.567206 | 0.170997 |
| ENSMUSG00000006731 | 0 | 0 | 2.562994 | 0.147831 |
| ENSMUSG00000029761 | 0 | 0 | 2.561794 | 0.196605 |
| ENSMUSG00000053062 | 0 | 0 | 2.55923 | 0.028724 |
| ENSMUSG00000070327 | 0 | 0 | 2.557119 | 0.070711 |
| ENSMUSG00000053334 | 0 | 0 | 2.551417 | 0.045914 |
| ENSMUSG00000072944 | 0 | 0 | 2.546388 | 0.338134 |
| ENSMUSG00000017774 | 0 | 0 | 2.546008 | 0.109703 |
| ENSMUSG00000026421 | 0 | 0 | 2.54571 | 0.132673 |
| ENSMUSG00000029762 | 0 | 0 | 2.543985 | 0.08678 |
| ENSMUSG00000033739 | 0 | 0 | 2.537958 | 0.071828 |
| ENSMUSG00000021662 | 0 | 0 | 2.535945 | 0.161887 |
| ENSMUSG00000027861 | 0 | 0 | 2.532201 | 0.71529 |
| ENSMUSG00000042549 | 0 | 0 | 2.531862 | 0.278126 |
| ENSMUSG00000029470 | 0 | 0 | 2.530128 | 0.056266 |
| ENSMUSG00000001604 | 0 | 0 | 2.529181 | 0.555472 |
| ENSMUSG00000079037 | 0 | 0 | 2.525023 | 0.001065 |
| ENSMUSG00000079057 | 0 | 0 | 2.523846 | 0.285216 |
| ENSMUSG00000031274 | 0 | 0 | 2.520645 | 0.110586 |
| ENSMUSG00000020580 | 0 | 0 | 2.519126 | 0.27838 |
| ENSMUSG00000042613 | 0 | 0 | 2.517555 | 0.042034 |
| ENSMUSG00000023805 | 0 | 0 | 2.516972 | 0.251188 |
| ENSMUSG00000056938 | 0 | 0 | 2.514801 | 0.076376 |
| ENSMUSG00000027204 | 0 | 0 | 2.507279 | 0.019883 |
| ENSMUSG00000025504 | 0 | 0 | 2.501178 | 0.113293 |
| ENSMUSG00000021388 | 0 | 0 | 2.500037 | 0.133597 |
| ENSMUSG00000004446 | 0 | 0 | 2.499746 | 0.090836 |
| ENSMUSG00000020937 | 0 | 0 | 2.499422 | 0.228434 |
| ENSMUSG00000032177 | 0 | 0 | 2.498837 | 0.227346 |
| ENSMUSG00000037679 | 0 | 0 | 2.491049 | 0.28362 |
| ENSMUSG00000074811 | 0 | 0 | 2.484972 | 0.007163 |
| ENSMUSG00000026748 | 0 | 0 | 2.48213 | 0.054579 |
| ENSMUSG00000021870 | 0 | 0 | 2.481858 | 0.202201 |
| ENSMUSG00000048332 | 0 | 0 | 2.481761 | 0.219622 |
| ENSMUSG00000031555 | 0 | 0 | 2.479753 | 0.028932 |
| ENSMUSG00000018821 | 0 | 0 | 2.476501 | 0.062947 |
| ENSMUSG00000051147 | 0 | 0 | 2.476198 | 0.446044 |
| ENSMUSG00000004085 | 0 | 0 | 2.475359 | 0.100525 |
| ENSMUSG00000026123 | 0 | 0 | 2.475233 | 0.015128 |
| ENSMUSG00000021668 | 0 | 0 | 2.471929 | 0.218327 |
| ENSMUSG00000030630 | 0 | 0 | 2.471243 | 0.258968 |
| ENSMUSG00000034993 | 0 | 0 | 2.469706 | 0.072238 |
| ENSMUSG00000052609 | 0 | 0 | 2.457148 | 0.012161 |
| ENSMUSG00000097810 | 0 | 0 | 2.454104 | 0.148659 |
| ENSMUSG00000032911 | 0 | 0 | 2.452286 | 0.299858 |
| ENSMUSG00000071984 | 0 | 0 | 2.449979 | 0.176936 |
| ENSMUSG00000022558 | 0 | 0 | 2.437887 | 0.110663 |
| ENSMUSG00000034810 | 0 | 0 | 2.433793 | 0.66173 |
| ENSMUSG00000028211 | 0 | 0 | 2.42884 | 0.023498 |
| ENSMUSG00000020773 | 0 | 0 | 2.423431 | 0.078902 |
| ENSMUSG00000020814 | 0 | 0 | 2.417547 | 0.11796 |
| ENSMUSG00000031591 | 0 | 0 | 2.416949 | 0.014096 |
| ENSMUSG00000038530 | 0 | 0 | 2.415116 | 0.119274 |
| ENSMUSG00000038375 | 0 | 0 | 2.407011 | 0.095648 |
| ENSMUSG00000054612 | 0 | 0 | 2.403754 | 0.087308 |
| ENSMUSG00000045730 | 0 | 0 | 2.39573 | 0.282368 |
| ENSMUSG00000027698 | 0 | 0 | 2.391406 | 0.141649 |
| ENSMUSG00000032754 | 0 | 0 | 2.39011 | 0.107165 |
| ENSMUSG00000030761 | 0 | 0 | 2.388083 | 0.774456 |
| ENSMUSG00000031887 | 0 | 0 | 2.38379 | 0.020715 |
| ENSMUSG00000022969 | 0 | 0 | 2.382963 | 0.082159 |
| ENSMUSG00000019796 | 0 | 0 | 2.382218 | 0.162266 |
| ENSMUSG00000026305 | 0 | 0 | 2.377876 | 0.086827 |
| ENSMUSG00000025140 | 0 | 0 | 2.374679 | 0.341586 |
| ENSMUSG00000078891 | 0 | 0 | 2.371901 | 0.120057 |
| ENSMUSG00000028251 | 0 | 0 | 2.368588 | 0.001931 |
| ENSMUSG00000033033 | 0 | 0 | 2.367361 | 0.215004 |
| ENSMUSG00000036533 | 0 | 0 | 2.365747 | 0.266515 |
| ENSMUSG00000021214 | 0 | 0 | 2.36445 | 0.081366 |
| ENSMUSG00000031387 | 0 | 0 | 2.364399 | 0.137412 |
| ENSMUSG00000037855 | 0 | 0 | 2.357569 | 0.05497 |
| ENSMUSG00000055447 | 0 | 0 | 2.348242 | 0.099555 |
| ENSMUSG00000026576 | 0 | 0 | 2.346711 | 0.611614 |
| ENSMUSG00000037239 | 0 | 0 | 2.346674 | 0.277813 |
| ENSMUSG00000052151 | 0 | 0 | 2.343504 | 0.052335 |
| ENSMUSG00000027230 | 0 | 0 | 2.341294 | 0.150266 |
| ENSMUSG00000029178 | 0 | 0 | 2.336014 | 0.189388 |
| ENSMUSG00000028671 | 0 | 0 | 2.331721 | 0.102214 |
| ENSMUSG00000045664 | 0 | 0 | 2.327696 | 0.274784 |
| ENSMUSG00000089901 | 0 | 0 | 2.32746 | 0.072878 |
| ENSMUSG00000060147 | 0 | 0 | 2.327015 | 0.05274 |
| ENSMUSG00000001473 | 0 | 0 | 2.326194 | 0.117936 |
| ENSMUSG00000018171 | 0 | 0 | 2.325876 | 0.015316 |
| ENSMUSG00000029446 | 0 | 0 | 2.325863 | 0.059537 |
| ENSMUSG00000092572 | 0 | 0 | 2.323953 | 0.438627 |
| ENSMUSG00000038172 | 0 | 0 | 2.321792 | 0.103656 |
| ENSMUSG00000025287 | 0 | 0 | 2.320833 | 0.118813 |
| ENSMUSG00000054855 | 0 | 0 | 2.319071 | 0.125592 |
| ENSMUSG00000028497 | 0 | 0 | 2.316737 | 0.308861 |
| ENSMUSG00000071656 | 0 | 0 | 2.314374 | 0.237972 |
| ENSMUSG00000032452 | 0 | 0 | 2.309617 | 0.035306 |
| ENSMUSG00000041889 | 0 | 0 | 2.307725 | 0.057439 |
| ENSMUSG00000033060 | 0 | 0 | 2.299809 | 0.158498 |
| ENSMUSG00000005043 | 0 | 0 | 2.295376 | 0.24317 |
| ENSMUSG00000028583 | 0 | 0 | 2.294036 | 0.091168 |
| ENSMUSG00000056185 | 0 | 0 | 2.289476 | 0.097314 |
| ENSMUSG00000038244 | 0 | 0 | 2.288933 | 0.223366 |
| ENSMUSG00000041608 | 0 | 0 | 2.285467 | 0.186521 |
| ENSMUSG00000054484 | 0 | 0 | 2.284482 | 0.090018 |
| ENSMUSG00000042363 | 0 | 0 | 2.280231 | 0.006913 |
| ENSMUSG00000097039 | 0 | 0 | 2.279891 | 0.123752 |
| ENSMUSG00000031207 | 0 | 0 | 2.279683 | 0.2174 |
| ENSMUSG00000029860 | 0 | 0 | 2.279012 | 0.175719 |
| ENSMUSG00000091243 | 0 | 0 | 2.272053 | 0.068674 |
| ENSMUSG00000026399 | 0 | 0 | 2.265054 | 0.114948 |
| ENSMUSG00000021094 | 0 | 0 | 2.264389 | 0.257351 |
| ENSMUSG00000026672 | 0 | 0 | 2.263588 | 0.002057 |
| ENSMUSG00000025757 | 0 | 0 | 2.261841 | 0.180848 |
| ENSMUSG00000060961 | 0 | 0 | 2.255998 | 0.009791 |
| ENSMUSG00000032332 | 0 | 0 | 2.255958 | 0.119905 |
| ENSMUSG00000028893 | 0 | 0 | 2.252393 | 0.175058 |
| ENSMUSG00000024063 | 0 | 0 | 2.249269 | 0.064068 |
| ENSMUSG00000025408 | 0 | 0 | 2.248446 | 0.131871 |
| ENSMUSG00000026519 | 0 | 0 | 2.246613 | 0.13163 |
| ENSMUSG00000025324 | 0 | 0 | 2.243282 | 0.002065 |
| ENSMUSG00000031375 | 0 | 0 | 2.240684 | 0.108258 |
| ENSMUSG00000021773 | 0 | 0 | 2.240178 | 0.144679 |
| ENSMUSG00000060802 | 0 | 0 | 2.238614 | 0.014974 |
| ENSMUSG00000044881 | 0 | 0 | 2.238581 | 0.287682 |
| ENSMUSG00000029070 | 0 | 0 | 2.23772 | 0.169819 |
| ENSMUSG00000039232 | 0 | 0 | 2.23424 | 0.07667 |
| ENSMUSG00000060260 | 0 | 0 | 2.232682 | 0.03773 |
| ENSMUSG00000021171 | 0 | 0 | 2.225645 | 0.011278 |
| ENSMUSG00000029634 | 0 | 0 | 2.224295 | 0.16375 |
| ENSMUSG00000040918 | 0 | 0 | 2.219238 | 0.036582 |
| ENSMUSG00000020023 | 0 | 0 | 2.217395 | 0.114975 |
| ENSMUSG00000052295 | 0 | 0 | 2.211974 | 0.423229 |
| ENSMUSG00000052688 | 0 | 0 | 2.210584 | 0.464559 |
| ENSMUSG00000090084 | 0 | 0 | 2.210189 | 0.0463 |
| ENSMUSG00000006445 | 0 | 0 | 2.209934 | 0.216288 |
| ENSMUSG00000032565 | 0 | 0 | 2.207292 | 0.36654 |
| ENSMUSG00000015653 | 0 | 0 | 2.204528 | 0.083425 |
| ENSMUSG00000055116 | 0 | 0 | 2.203469 | 0.104585 |
| ENSMUSG00000035916 | 0 | 0 | 2.196851 | 0.310502 |
| ENSMUSG00000039182 | 0 | 0 | 2.195017 | 0.160552 |
| ENSMUSG00000005087 | 0 | 0 | 2.192904 | 0.138752 |
| ENSMUSG00000049950 | 0 | 0 | 2.189874 | 0.289897 |
| ENSMUSG00000061603 | 0 | 0 | 2.187519 | 0.093101 |
| ENSMUSG00000022892 | 0 | 0 | 2.186774 | 0.021276 |
| ENSMUSG00000069516 | 0 | 0 | 2.180208 | 0.303499 |
| ENSMUSG00000031490 | 0 | 0 | 2.178904 | 0.083348 |
| ENSMUSG00000026535 | 0 | 0 | 2.175742 | 0.017695 |
| ENSMUSG00000028007 | 0 | 0 | 2.172154 | 0.051443 |
| ENSMUSG00000015094 | 0 | 0 | 2.170964 | 0.025488 |
| ENSMUSG00000025521 | 0 | 0 | 2.170789 | 0.060867 |
| ENSMUSG00000025856 | 0 | 0 | 2.164547 | 0.064639 |
| ENSMUSG00000009376 | 0 | 0 | 2.16344 | 0.445277 |
| ENSMUSG00000061232 | 0 | 0 | 2.161027 | 0.180738 |
| ENSMUSG00000024087 | 0 | 0 | 2.153157 | 0.009229 |
| ENSMUSG00000029007 | 0 | 0 | 2.151475 | 0.136416 |
| ENSMUSG00000040212 | 0 | 0 | 2.148105 | 0.13216 |
| ENSMUSG00000022565 | 0 | 0 | 2.145403 | 0.015123 |
| ENSMUSG00000036002 | 0 | 0 | 2.144759 | 0.019626 |
| ENSMUSG00000035517 | 0 | 0 | 2.142292 | 0.021824 |
| ENSMUSG00000021867 | 0 | 0 | 2.14116 | 0.309108 |
| ENSMUSG00000072676 | 0 | 0 | 2.133416 | 0.323977 |
| ENSMUSG00000029718 | 0 | 0 | 2.130611 | 0.133504 |
| ENSMUSG00000039361 | 0 | 0 | 2.127375 | 0.081127 |
| ENSMUSG00000031342 | 0 | 0 | 2.12549 | 0.191813 |
| ENSMUSG00000051811 | 0 | 0 | 2.124704 | 0.761367 |
| ENSMUSG00000031519 | 0 | 0 | 2.122359 | 0.013926 |
| ENSMUSG00000007891 | 0 | 0 | 2.114972 | 0.006333 |
| ENSMUSG00000030353 | 0 | 0 | 2.111029 | 0.411464 |
| ENSMUSG00000036534 | 0 | 0 | 2.108872 | 0.141677 |
| ENSMUSG00000032656 | 0 | 0 | 2.107869 | 0.002179 |
| ENSMUSG00000032372 | 0 | 0 | 2.105744 | 0.045773 |
| ENSMUSG00000026193 | 0 | 0 | 2.105237 | 0.191287 |
| ENSMUSG00000028600 | 0 | 0 | 2.103628 | 0.036602 |
| ENSMUSG00000020682 | 0 | 0 | 2.095284 | 0.021452 |
| ENSMUSG00000019832 | 0 | 0 | 2.094806 | 0.130737 |
| ENSMUSG00000020473 | 0 | 0 | 2.090657 | 0.084305 |
| ENSMUSG00000021493 | 0 | 0 | 2.088846 | 0.173171 |
| ENSMUSG00000048482 | 0 | 0 | 2.087245 | 0.25312 |
| ENSMUSG00000011463 | 0 | 0 | 2.077437 | 0.140999 |
| ENSMUSG00000020032 | 0 | 0 | 2.073013 | 0.058263 |
| ENSMUSG00000020142 | 0 | 0 | 2.072423 | 0.018492 |
| ENSMUSG00000050578 | 0 | 0 | 2.072047 | 0.544964 |
| ENSMUSG00000028542 | 0 | 0 | 2.071072 | 0.031872 |
| ENSMUSG00000024074 | 0 | 0 | 2.066672 | 0.287082 |
| ENSMUSG00000029364 | 0 | 0 | 2.065072 | 0.138174 |
| ENSMUSG00000034853 | 0 | 0 | 2.061829 | 0.024794 |
| ENSMUSG00000011256 | 0 | 0 | 2.056751 | 0.033222 |
| ENSMUSG00000024942 | 0 | 0 | 2.056045 | 0.117315 |
| ENSMUSG00000024854 | 0 | 0 | 2.055116 | 0.090127 |
| ENSMUSG00000024544 | 0 | 0 | 2.054379 | 0.005632 |
| ENSMUSG00000036820 | 0 | 0 | 2.051367 | 0.020866 |
| ENSMUSG00000016487 | 0 | 0 | 2.051164 | 0.12462 |
| ENSMUSG00000041313 | 0 | 0 | 2.048458 | 0.103993 |
| ENSMUSG00000003873 | 0 | 0 | 2.046776 | 0.07908 |
| ENSMUSG00000026958 | 0 | 0 | 2.039083 | 0.014292 |
| ENSMUSG00000000693 | 0 | 0 | 2.038176 | 0.205907 |
| ENSMUSG00000025278 | 0 | 0 | 2.0354 | 0.281447 |
| ENSMUSG00000052942 | 0 | 0 | 2.034748 | 0.098305 |
| ENSMUSG00000027087 | 0 | 0 | 2.033034 | 0.072151 |
| ENSMUSG00000033855 | 0 | 0 | 2.032286 | 0.147549 |
| ENSMUSG00000039286 | 0 | 0 | 2.031842 | 0.073357 |
| ENSMUSG00000039943 | 0 | 0 | 2.023688 | 0.331786 |
| ENSMUSG00000045827 | 0 | 0 | 2.020727 | 0.233885 |
| ENSMUSG00000020467 | 0 | 0 | 2.019799 | 0.055374 |
| ENSMUSG00000026479 | 0 | 0 | 2.018758 | 0.438905 |
| ENSMUSG00000020828 | 0 | 0 | 2.017784 | 0.064051 |
| ENSMUSG00000024769 | 0 | 0 | 2.009551 | 0.162266 |
| ENSMUSG00000012889 | 0 | 0 | 2.009286 | 0.206024 |
| ENSMUSG00000073411 | 0 | 0 | 2.005238 | 0.09051 |
| ENSMUSG00000054988 | 0 | 0 | 2.001127 | 0.416356 |
| ENSMUSG00000022505 | 0 | 0 | 1.997266 | 0.001796 |
| ENSMUSG00000054474 | 0 | 0 | 1.990695 | 0.485281 |
| ENSMUSG00000002105 | 0 | 0 | 1.990226 | 0.102559 |
| ENSMUSG00000059883 | 0 | 0 | 1.990221 | 0.074321 |
| ENSMUSG00000000753 | 0 | 0 | 1.988355 | 0.180079 |
| ENSMUSG00000038068 | 0 | 0 | 1.987347 | 0.217185 |
| ENSMUSG00000041886 | 0 | 0 | 1.983773 | 0.172778 |
| ENSMUSG00000045790 | 0 | 0 | 1.982245 | 0.050758 |
| ENSMUSG00000024511 | 0 | 0 | 1.980549 | 0.269588 |
| ENSMUSG00000021585 | 0 | 0 | 1.979715 | 0.107556 |
| ENSMUSG00000002257 | 0 | 0 | 1.979672 | 0.145795 |
| ENSMUSG00000050295 | 0 | 0 | 1.977023 | 0.148346 |
| ENSMUSG00000000275 | 0 | 0 | 1.976582 | 0.122682 |
| ENSMUSG00000022184 | 0 | 0 | 1.976058 | 0.121334 |
| ENSMUSG00000028159 | 0 | 0 | 1.972927 | 0.014889 |
| ENSMUSG00000023088 | 0 | 0 | 1.972438 | 0.059258 |
| ENSMUSG00000045374 | 0 | 0 | 1.967883 | 0.092293 |
| ENSMUSG00000037071 | 0 | 0 | 1.965796 | 0.19008 |
| ENSMUSG00000025212 | 0 | 0 | 1.961911 | 0.079165 |
| ENSMUSG00000045251 | 0 | 0 | 1.961393 | 0.252391 |
| ENSMUSG00000052430 | 0 | 0 | 1.9594 | 0.266465 |
| ENSMUSG00000006390 | 0 | 0 | 1.959373 | 0.251056 |
| ENSMUSG00000029094 | 0 | 0 | 1.957929 | 0.028113 |
| ENSMUSG00000062380 | 0 | 0 | 1.955903 | 0.046923 |
| ENSMUSG00000032243 | 0 | 0 | 1.954458 | 0.293209 |
| ENSMUSG00000031558 | 0 | 0 | 1.953349 | 0.073921 |
| ENSMUSG00000070942 | 0 | 0 | 1.952504 | 0.134603 |
| ENSMUSG00000019842 | 0 | 0 | 1.951144 | 0.043521 |
| ENSMUSG00000002897 | 0 | 0 | 1.949735 | 0.165971 |
| ENSMUSG00000017764 | 0 | 0 | 1.949575 | 0.340727 |
| ENSMUSG00000028466 | 0 | 0 | 1.946628 | 0.040684 |
| ENSMUSG00000097993 | 0 | 0 | 1.942792 | 0.028869 |
| ENSMUSG00000018042 | 0 | 0 | 1.942127 | 0.03347 |
| ENSMUSG00000050721 | 0 | 0 | 1.94019 | 0.049507 |
| ENSMUSG00000032507 | 0 | 0 | 1.934054 | 0.090803 |
| ENSMUSG00000020736 | 0 | 0 | 1.931109 | 0.092636 |
| ENSMUSG00000030748 | 0 | 0 | 1.930897 | 0.157338 |
| ENSMUSG00000026767 | 0 | 0 | 1.93069 | 0.133606 |
| ENSMUSG00000064215 | 0 | 0 | 1.930212 | 0.093503 |
| ENSMUSG00000029313 | 0 | 0 | 1.929727 | 0.143306 |
| ENSMUSG00000059714 | 0 | 0 | 1.928906 | 0.051318 |
| ENSMUSG00000093989 | 0 | 0 | 1.927538 | 0.186266 |
| ENSMUSG00000021477 | 0 | 0 | 1.926465 | 0.183443 |
| ENSMUSG00000074364 | 0 | 0 | 1.917138 | 0.128252 |
| ENSMUSG00000063430 | 0 | 0 | 1.914105 | 0.062067 |
| ENSMUSG00000026109 | 0 | 0 | 1.913901 | 0.115791 |
| ENSMUSG00000029482 | 0 | 0 | 1.912868 | 0.156098 |
| ENSMUSG00000030609 | 0 | 0 | 1.910434 | 0.267235 |
| ENSMUSG00000030157 | 0 | 0 | 1.909572 | 0.04553 |
| ENSMUSG00000048277 | 0 | 0 | 1.907642 | 0.090834 |
| ENSMUSG00000025068 | 0 | 0 | 1.907113 | 0.01478 |
| ENSMUSG00000047428 | 0 | 0 | 1.906808 | 0.30551 |
| ENSMUSG00000033998 | 0 | 0 | 1.905072 | 0.005963 |
| ENSMUSG00000034744 | 0 | 0 | 1.904611 | 0.0452 |
| ENSMUSG00000039601 | 0 | 0 | 1.903622 | 0.292088 |
| ENSMUSG00000045665 | 0 | 0 | 1.903469 | 0.078301 |
| ENSMUSG00000020132 | 0 | 0 | 1.902278 | 0.099547 |
| ENSMUSG00000028059 | 0 | 0 | 1.896361 | 0.057233 |
| ENSMUSG00000022844 | 0 | 0 | 1.893658 | 0.321825 |
| ENSMUSG00000026727 | 0 | 0 | 1.893303 | 0.183979 |
| ENSMUSG00000028164 | 0 | 0 | 1.888293 | 0.227424 |
| ENSMUSG00000050912 | 0 | 0 | 1.886344 | 0.019166 |
| ENSMUSG00000032643 | 0 | 0 | 1.885065 | 0.442877 |
| ENSMUSG00000020100 | 0 | 0 | 1.882062 | 0.112006 |
| ENSMUSG00000031328 | 0 | 0 | 1.881711 | 0 |
| ENSMUSG00000027763 | 0 | 0 | 1.881528 | 0.160994 |
| ENSMUSG00000054150 | 0 | 0 | 1.881489 | 0.053542 |
| ENSMUSG00000026980 | 0 | 0 | 1.880546 | 0.192776 |
| ENSMUSG00000026604 | 0 | 0 | 1.87857 | 0.076319 |
| ENSMUSG00000024065 | 0 | 0 | 1.877284 | 0.009819 |
| ENSMUSG00000031119 | 0 | 0 | 1.875532 | 0.179981 |
| ENSMUSG00000029185 | 0 | 0 | 1.875324 | 0.081065 |
| ENSMUSG00000048040 | 0 | 0 | 1.874786 | 0.024274 |
| ENSMUSG00000006356 | 0 | 0 | 1.874758 | 0.046875 |
| ENSMUSG00000046169 | 0 | 0 | 1.874408 | 0.089451 |
| ENSMUSG00000028041 | 0 | 0 | 1.873476 | 0.009765 |
| ENSMUSG00000045629 | 0 | 0 | 1.872789 | 0.153029 |
| ENSMUSG00000040010 | 0 | 0 | 1.871953 | 0.127809 |
| ENSMUSG00000032702 | 0 | 0 | 1.870768 | 0.022072 |
| ENSMUSG00000000126 | 0 | 0 | 1.868319 | 0.017157 |
| ENSMUSG00000036966 | 0 | 0 | 1.865187 | 0.077742 |
| ENSMUSG00000031129 | 0 | 0 | 1.859221 | 0.057469 |
| ENSMUSG00000023224 | 0 | 0 | 1.858919 | 0.243106 |
| ENSMUSG00000022906 | 0 | 0 | 1.858879 | 0.126536 |
| ENSMUSG00000018819 | 0 | 0 | 1.858871 | 0.025084 |
| ENSMUSG00000029651 | 0 | 0 | 1.856299 | 0.046785 |
| ENSMUSG00000026796 | 0 | 0 | 1.855312 | 0.137026 |
| ENSMUSG00000019775 | 0 | 0 | 1.854091 | 0.039213 |
| ENSMUSG00000027134 | 0 | 0 | 1.853843 | 0.01884 |
| ENSMUSG00000006717 | 0 | 0 | 1.853811 | 0.117987 |
| ENSMUSG00000022074 | 0 | 0 | 1.852802 | 0.08875 |
| ENSMUSG00000025283 | 0 | 0 | 1.851698 | 0.046611 |
| ENSMUSG00000075705 | 0 | 0 | 1.850478 | 0.134343 |
| ENSMUSG00000031007 | 0 | 0 | 1.848546 | 0.033216 |
| ENSMUSG00000006931 | 0 | 0 | 1.847246 | 0.177941 |
| ENSMUSG00000030605 | 0 | 0 | 1.845084 | 0.113209 |
| ENSMUSG00000029777 | 0 | 0 | 1.842481 | 0.005774 |
| ENSMUSG00000037966 | 0 | 0 | 1.836555 | 0.032264 |
| ENSMUSG00000073643 | 0 | 0 | 1.835779 | 0.177907 |
| ENSMUSG00000026072 | 0 | 0 | 1.833724 | 0.211042 |
| ENSMUSG00000029759 | 0 | 0 | 1.83191 | 0.242507 |
| ENSMUSG00000015932 | 0 | 0 | 1.831023 | 0.1639 |
| ENSMUSG00000030347 | 0 | 0 | 1.829795 | 0.133079 |
| ENSMUSG00000025854 | 0 | 0 | 1.828881 | 0.077763 |
| ENSMUSG00000030659 | 0 | 0 | 1.828516 | 0.134553 |
| ENSMUSG00000036918 | 0 | 0 | 1.820296 | 0.00091 |
| ENSMUSG00000005667 | 0 | 0 | 1.820042 | 0.116774 |
| ENSMUSG00000033542 | 0 | 0 | 1.818569 | 0.184705 |
| ENSMUSG00000019528 | 0 | 0 | 1.817647 | 0.094062 |
| ENSMUSG00000028480 | 0 | 0 | 1.814793 | 0.027298 |
| ENSMUSG00000069633 | 0 | 0 | 1.813326 | 0.041177 |
| ENSMUSG00000078317 | 0 | 0 | 1.813272 | 0.334948 |
| ENSMUSG00000081534 | 0 | 0 | 1.811292 | 0.136098 |
| ENSMUSG00000004768 | 0 | 0 | 1.811011 | 0.080274 |
| ENSMUSG00000037649 | 0 | 0 | 1.810952 | 0.161795 |
| ENSMUSG00000046532 | 0 | 0 | 1.808155 | 0.134635 |
| ENSMUSG00000044551 | 0 | 0 | 1.807894 | 0.194443 |
| ENSMUSG00000020689 | 0 | 0 | 1.807633 | 0.273783 |
| ENSMUSG00000046731 | 0 | 0 | 1.806121 | 0.378816 |
| ENSMUSG00000027560 | 0 | 0 | 1.803106 | 0.149235 |
| ENSMUSG00000041324 | 0 | 0 | 1.801579 | 0.147249 |
| ENSMUSG00000039910 | 0 | 0 | 1.800879 | 0.036233 |
| ENSMUSG00000039461 | 0 | 0 | 1.80016 | 0.134603 |
| ENSMUSG00000040433 | 0 | 0 | 1.799956 | 0.080713 |
| ENSMUSG00000027859 | 0 | 0 | 1.798192 | 0.230553 |
| ENSMUSG00000028613 | 0 | 0 | 1.795436 | 0.295258 |
| ENSMUSG00000051319 | 0 | 0 | 1.793892 | 0.094032 |
| ENSMUSG00000025083 | 0 | 0 | 1.793399 | 0.419593 |
| ENSMUSG00000048707 | 0 | 0 | 1.790104 | 0.099253 |
| ENSMUSG00000029175 | 0 | 0 | 1.789494 | 0.144827 |
| ENSMUSG00000071083 | 0 | 0 | 1.789036 | 0.275975 |
| ENSMUSG00000028019 | 0 | 0 | 1.78833 | 0.06904 |
| ENSMUSG00000024423 | 0 | 0 | 1.785336 | 0.057355 |
| ENSMUSG00000053846 | 0 | 0 | 1.784199 | 0.207449 |
| ENSMUSG00000038028 | 0 | 0 | 1.784072 | 0.395761 |
| ENSMUSG00000024955 | 0 | 0 | 1.782759 | 0.152259 |
| ENSMUSG00000010755 | 0 | 0 | 1.782313 | 0.069246 |
| ENSMUSG00000062101 | 0 | 0 | 1.781004 | 0.222449 |
| ENSMUSG00000019872 | 0 | 0 | 1.778003 | 0.110847 |
| ENSMUSG00000021665 | 0 | 0 | 1.77794 | 0.046283 |
| ENSMUSG00000020225 | 0 | 0 | 1.777705 | 0.0697 |
| ENSMUSG00000064284 | 0 | 0 | 1.77724 | 0.207798 |
| ENSMUSG00000024899 | 0 | 0 | 1.773897 | 0.083852 |
| ENSMUSG00000020044 | 0 | 0 | 1.77273 | 0.067706 |
| ENSMUSG00000020261 | 0 | 0 | 1.770775 | 0.054038 |
| ENSMUSG00000025507 | 0 | 0 | 1.767093 | 0.526209 |
| ENSMUSG00000043311 | 0 | 0 | 1.7637 | 0.260439 |
| ENSMUSG00000030598 | 0 | 0 | 1.762946 | 0.10879 |
| ENSMUSG00000028179 | 0 | 0 | 1.762736 | 0.029746 |
| ENSMUSG00000038807 | 0 | 0 | 1.759755 | 0.211081 |
| ENSMUSG00000017760 | 0 | 0 | 1.755799 | 0.079095 |
| ENSMUSG00000004994 | 0 | 0 | 1.75492 | 0.298337 |
| ENSMUSG00000079317 | 0 | 0 | 1.748939 | 0.219957 |
| ENSMUSG00000016128 | 0 | 0 | 1.745416 | 0.025267 |
| ENSMUSG00000029552 | 0 | 0 | 1.745248 | 0.145984 |
| ENSMUSG00000089917 | 0 | 0 | 1.74291 | 0.055453 |
| ENSMUSG00000039470 | 0 | 0 | 1.742142 | 0.19931 |
| ENSMUSG00000033389 | 0 | 0 | 1.74155 | 0.082655 |
| ENSMUSG00000027777 | 0 | 0 | 1.741515 | 0.16606 |
| ENSMUSG00000050088 | 0 | 0 | 1.739263 | 0.049132 |
| ENSMUSG00000026578 | 0 | 0 | 1.738486 | 0.183008 |
| ENSMUSG00000034327 | 0 | 0 | 1.738061 | 0.126547 |
| ENSMUSG00000055172 | 0 | 0 | 1.736285 | 0.331783 |
| ENSMUSG00000023960 | 0 | 0 | 1.735033 | 0.218376 |
| ENSMUSG00000094935 | 0 | 0 | 1.733616 | 0.3996 |
| ENSMUSG00000043336 | 0 | 0 | 1.732241 | 0.085723 |
| ENSMUSG00000024875 | 0 | 0 | 1.730526 | 6.66E-05 |
| ENSMUSG00000057554 | 0 | 0 | 1.728917 | 0.082151 |
| ENSMUSG00000030101 | 0 | 0 | 1.728876 | 0.13121 |
| ENSMUSG00000039747 | 0 | 0 | 1.727217 | 0.182258 |
| ENSMUSG00000021959 | 0 | 0 | 1.726736 | 0.176339 |
| ENSMUSG00000034708 | 0 | 0 | 1.723606 | 0.076026 |
| ENSMUSG00000050973 | 0 | 0 | 1.72344 | 0.420063 |
| ENSMUSG00000068699 | 0 | 0 | 1.722578 | 0.076174 |
| ENSMUSG00000024897 | 0 | 0 | 1.719693 | 0.177116 |
| ENSMUSG00000028656 | 0 | 0 | 1.718746 | 0.076857 |
| ENSMUSG00000022564 | 0 | 0 | 1.718388 | 0.159136 |
| ENSMUSG00000030538 | 0 | 0 | 1.714483 | 0.128608 |
| ENSMUSG00000062944 | 0 | 0 | 1.713953 | 0.152936 |
| ENSMUSG00000024493 | 0 | 0 | 1.712342 | 0.098539 |
| ENSMUSG00000052102 | 0 | 0 | 1.710581 | 0.012807 |
| ENSMUSG00000017466 | 0 | 0 | 1.70976 | 0.038377 |
| ENSMUSG00000026725 | 0 | 0 | 1.709662 | 0.264372 |
| ENSMUSG00000095595 | 0 | 0 | 1.709483 | 0.020556 |
| ENSMUSG00000002083 | 0 | 0 | 1.708439 | 0.397442 |
| ENSMUSG00000020432 | 0 | 0 | 1.706042 | 0.076951 |
| ENSMUSG00000025964 | 0 | 0 | 1.704239 | 0.126122 |
| ENSMUSG00000030401 | 0 | 0 | 1.703861 | 0.166495 |
| ENSMUSG00000034614 | 0 | 0 | 1.703798 | 0.121142 |
| ENSMUSG00000030615 | 0 | 0 | 1.70332 | 0.065825 |
| ENSMUSG00000019916 | 0 | 0 | 1.702628 | 0.096138 |
| ENSMUSG00000039623 | 0 | 0 | 1.702044 | 0.089175 |
| ENSMUSG00000029851 | 0 | 0 | 1.70186 | 0.051553 |
| ENSMUSG00000064105 | 0 | 0 | 1.701497 | 0.156291 |
| ENSMUSG00000042292 | 0 | 0 | 1.701376 | 0.327394 |
| ENSMUSG00000003518 | 0 | 0 | 1.700608 | 0.110481 |
| ENSMUSG00000047963 | 0 | 0 | 1.700503 | 0.086794 |
| ENSMUSG00000047123 | 0 | 0 | 1.698412 | 0.244133 |
| ENSMUSG00000021814 | 0 | 0 | 1.696916 | 0.090163 |
| ENSMUSG00000021583 | 0 | 0 | 1.694796 | 0.096668 |
| ENSMUSG00000028048 | 0 | 0 | 1.694768 | 0.005253 |
| ENSMUSG00000047414 | 0 | 0 | 1.694333 | 0.012381 |
| ENSMUSG00000089945 | 0 | 0 | 1.693882 | 0.007847 |
| ENSMUSG00000061288 | 0 | 0 | 1.693254 | 0.24565 |
| ENSMUSG00000046027 | 0 | 0 | 1.690578 | 0.060251 |
| ENSMUSG00000019158 | 0 | 0 | 1.687753 | 0.027401 |
| ENSMUSG00000086502 | 0 | 0 | 1.686994 | 0.091839 |
| ENSMUSG00000025647 | 0 | 0 | 1.684024 | 0.006131 |
| ENSMUSG00000027605 | 0 | 0 | 1.683783 | 0.128233 |
| ENSMUSG00000020846 | 0 | 0 | 1.683513 | 0.00248 |
| ENSMUSG00000026131 | 0 | 0 | 1.678942 | 0.137252 |
| ENSMUSG00000030077 | 0 | 0 | 1.676848 | 0.098935 |
| ENSMUSG00000037852 | 0 | 0 | 1.676324 | 0.075775 |
| ENSMUSG00000016382 | 0 | 0 | 1.676129 | 0.253012 |
| ENSMUSG00000075702 | 0 | 0 | 1.675777 | 0.021749 |
| ENSMUSG00000031337 | 0 | 0 | 1.675177 | 0.162678 |
| ENSMUSG00000024981 | 0 | 0 | 1.672654 | 0.112958 |
| ENSMUSG00000032504 | 0 | 0 | 1.672608 | 0.102033 |
| ENSMUSG00000022533 | 0 | 0 | 1.67257 | 0.272276 |
| ENSMUSG00000022295 | 0 | 0 | 1.671094 | 0.058188 |
| ENSMUSG00000028518 | 0 | 0 | 1.670966 | 0.259198 |
| ENSMUSG00000068220 | 0 | 0 | 1.670279 | 0.038436 |
| ENSMUSG00000016534 | 0 | 0 | 1.670231 | 0.112416 |
| ENSMUSG00000044447 | 0 | 0 | 1.668457 | 0.147261 |
| ENSMUSG00000025225 | 0 | 0 | 1.667916 | 0.004572 |
| ENSMUSG00000041660 | 0 | 0 | 1.667702 | 0.103081 |
| ENSMUSG00000048264 | 0 | 0 | 1.667554 | 0.000463 |
| ENSMUSG00000069892 | 0 | 0 | 1.665975 | 0.373467 |
| ENSMUSG00000031461 | 0 | 0 | 1.664918 | 0.346875 |
| ENSMUSG00000032336 | 0 | 0 | 1.663824 | 0.030833 |
| ENSMUSG00000028194 | 0 | 0 | 1.662958 | 0.163424 |
| ENSMUSG00000020354 | 0 | 0 | 1.661928 | 0.151128 |
| ENSMUSG00000043531 | 0 | 0 | 1.661863 | 0.241035 |
| ENSMUSG00000021831 | 0 | 0 | 1.659605 | 0.120384 |
| ENSMUSG00000051323 | 0 | 0 | 1.658002 | 0.030651 |
| ENSMUSG00000017747 | 0 | 0 | 1.656943 | 0.033454 |
| ENSMUSG00000041488 | 0 | 0 | 1.654134 | 0.144096 |
| ENSMUSG00000026688 | 0 | 0 | 1.652734 | 0.120015 |
| ENSMUSG00000048439 | 0 | 0 | 1.651003 | 0.01814 |
| ENSMUSG00000020056 | 0 | 0 | 1.650926 | 0.089416 |
| ENSMUSG00000033540 | 0 | 0 | 1.649042 | 0.01936 |
| ENSMUSG00000049396 | 0 | 0 | 1.648983 | 0.011443 |
| ENSMUSG00000031513 | 0 | 0 | 1.647996 | 0.026371 |
| ENSMUSG00000061666 | 0 | 0 | 1.647978 | 0.424612 |
| ENSMUSG00000041028 | 0 | 0 | 1.647179 | 0.000938 |
| ENSMUSG00000023048 | 0 | 0 | 1.645947 | 0.113433 |
| ENSMUSG00000022772 | 0 | 0 | 1.645739 | 0.104687 |
| ENSMUSG00000025171 | 0 | 0 | 1.639711 | 0.115703 |
| ENSMUSG00000014444 | 0 | 0 | 1.637786 | 0.060631 |
| ENSMUSG00000030536 | 0 | 0 | 1.637516 | 0.124052 |
| ENSMUSG00000002580 | 0 | 0 | 1.637432 | 0.025007 |
| ENSMUSG00000041923 | 0 | 0 | 1.63719 | 0.040421 |
| ENSMUSG00000031557 | 0 | 0 | 1.636849 | 0.349435 |
| ENSMUSG00000014668 | 0 | 0 | 1.636697 | 0.174515 |
| ENSMUSG00000027035 | 0 | 0 | 1.634725 | 0.029039 |
| ENSMUSG00000045441 | 0 | 0 | 1.633355 | 0.155258 |
| ENSMUSG00000024130 | 0 | 0 | 1.632096 | 0.121278 |
| ENSMUSG00000050188 | 0 | 0 | 1.628953 | 0.345193 |
| ENSMUSG00000019975 | 0 | 0 | 1.62638 | 0.011985 |
| ENSMUSG00000004891 | 0 | 0 | 1.624453 | 0.135529 |
| ENSMUSG00000020743 | 0 | 0 | 1.622022 | 0.111916 |
| ENSMUSG00000047821 | 0 | 0 | 1.621834 | 0.162202 |
| ENSMUSG00000031897 | 0 | 0 | 1.62175 | 0.056747 |
| ENSMUSG00000028980 | 0 | 0 | 1.621044 | 0.052224 |
| ENSMUSG00000072437 | 0 | 0 | 1.62094 | 0.096512 |
| ENSMUSG00000031767 | 0 | 0 | 1.620265 | 0.065313 |
| ENSMUSG00000004846 | 0 | 0 | 1.61943 | 0.039137 |
| ENSMUSG00000028962 | 0 | 0 | 1.616818 | 0.165188 |
| ENSMUSG00000031349 | 0 | 0 | 1.614535 | 0.127726 |
| ENSMUSG00000026637 | 0 | 0 | 1.613888 | 0.078361 |
| ENSMUSG00000028763 | 0 | 0 | 1.61381 | 0.112013 |
| ENSMUSG00000025357 | 0 | 0 | 1.613295 | 0.202498 |
| ENSMUSG00000024587 | 0 | 0 | 1.611928 | 0.049442 |
| ENSMUSG00000053819 | 0 | 0 | 1.610792 | 0.022648 |
| ENSMUSG00000056383 | 0 | 0 | 1.608661 | 0.017343 |
| ENSMUSG00000030220 | 0 | 0 | 1.608637 | 0.039892 |
| ENSMUSG00000031438 | 0 | 0 | 1.606364 | 0.024053 |
| ENSMUSG00000030339 | 0 | 0 | 1.606101 | 0.051348 |
| ENSMUSG00000042284 | 0 | 0 | 1.605343 | 0.089875 |
| ENSMUSG00000007038 | 0 | 0 | 1.603908 | 0.087589 |
| ENSMUSG00000061778 | 0 | 0 | 1.60256 | 0.086574 |
| ENSMUSG00000020829 | 0 | 0 | 1.601078 | 0.012653 |
| ENSMUSG00000031447 | 0 | 0 | 1.600566 | 0.128532 |
| ENSMUSG00000001555 | 0 | 0 | 1.595887 | 0.06346 |
| ENSMUSG00000021253 | 0 | 0 | 1.592591 | 0.145699 |
| ENSMUSG00000014771 | 0 | 0 | 1.584363 | 0.022639 |
| ENSMUSG00000034205 | 0 | 0 | 1.58364 | 0.038446 |
| ENSMUSG00000029859 | 0 | 0 | 1.583206 | 0.331306 |
| ENSMUSG00000015143 | 0 | 0 | 1.582345 | 0.145873 |
| ENSMUSG00000020184 | 0 | 0 | 1.581192 | 0.088337 |
| ENSMUSG00000037190 | 0 | 0 | 1.580325 | 0.054357 |
| ENSMUSG00000055670 | 0 | 0 | 1.579443 | 0.248666 |
| ENSMUSG00000078453 | 0 | 0 | 1.579002 | 0.165531 |
| ENSMUSG00000060032 | 0 | 0 | 1.578465 | 0.114494 |
| ENSMUSG00000015002 | 0 | 0 | 1.574543 | 0.08227 |
| ENSMUSG00000000278 | 0 | 0 | 1.572887 | 0.012403 |
| ENSMUSG00000054469 | 0 | 0 | 1.567917 | 0.045127 |
| ENSMUSG00000041390 | 0 | 0 | 1.564668 | 0.200428 |
| ENSMUSG00000020605 | 0 | 0 | 1.563229 | 0.176704 |
| ENSMUSG00000015575 | 0 | 0 | 1.562986 | 0.026847 |
| ENSMUSG00000041779 | 0 | 0 | 1.561279 | 0.26578 |
| ENSMUSG00000031563 | 0 | 0 | 1.560322 | 0.048598 |
| ENSMUSG00000035969 | 0 | 0 | 1.555885 | 0.051114 |
| ENSMUSG00000051978 | 0 | 0 | 1.552934 | 0.184257 |
| ENSMUSG00000027995 | 0 | 0 | 1.552382 | 0.092926 |
| ENSMUSG00000032802 | 0 | 0 | 1.551451 | 0.054297 |
| ENSMUSG00000060923 | 0 | 0 | 1.55116 | 0.091245 |
| ENSMUSG00000024436 | 0 | 0 | 1.550143 | 0.131441 |
| ENSMUSG00000049775 | 0 | 0 | 1.548535 | 0.113186 |
| ENSMUSG00000026837 | 0 | 0 | 1.548301 | 0 |
| ENSMUSG00000033809 | 0 | 0 | 1.547822 | 0.070922 |
| ENSMUSG00000041301 | 0 | 0 | 1.54757 | 0.149951 |
| ENSMUSG00000020272 | 0 | 0 | 1.543612 | 0.02748 |
| ENSMUSG00000039787 | 0 | 0 | 1.542718 | 0.180627 |
| ENSMUSG00000048677 | 0 | 0 | 1.54265 | 0.311318 |
| ENSMUSG00000004665 | 0 | 0 | 1.539344 | 0.073826 |
| ENSMUSG00000053641 | 0 | 0 | 1.539012 | 0.098897 |
| ENSMUSG00000033793 | 0 | 0 | 1.53814 | 0.006369 |
| ENSMUSG00000052512 | 0 | 0 | 1.537689 | 0.326306 |
| ENSMUSG00000046808 | 0 | 0 | 1.534189 | 0.064542 |
| ENSMUSG00000038181 | 0 | 0 | 1.53329 | 0.065318 |
| ENSMUSG00000027907 | 0 | 0 | 1.533188 | 0.020652 |
| ENSMUSG00000037349 | 0 | 0 | 1.530771 | 0.326073 |
| ENSMUSG00000038604 | 0 | 0 | 1.53018 | 0.190386 |
| ENSMUSG00000030287 | 0 | 0 | 1.530038 | 0.073758 |
| ENSMUSG00000036782 | 0 | 0 | 1.529844 | 0.205692 |
| ENSMUSG00000021360 | 0 | 0 | 1.529363 | 0.17408 |
| ENSMUSG00000072825 | 0 | 0 | 1.528178 | 0.089699 |
| ENSMUSG00000003812 | 0 | 0 | 1.52805 | 0.159502 |
| ENSMUSG00000003849 | 0 | 0 | 1.524356 | 0.009325 |
| ENSMUSG00000027999 | 0 | 0 | 1.523037 | 0.161715 |
| ENSMUSG00000073016 | 0 | 0 | 1.522365 | 0.142604 |
| ENSMUSG00000048772 | 0 | 0 | 1.52159 | 0.19947 |
| ENSMUSG00000027533 | 0 | 0 | 1.521577 | 0.152116 |
| ENSMUSG00000036402 | 0 | 0 | 1.518495 | 0.081064 |
| ENSMUSG00000025790 | 0 | 0 | 1.518254 | 0.068807 |
| ENSMUSG00000020053 | 0 | 0 | 1.517834 | 0.014026 |
| ENSMUSG00000003528 | 0 | 0 | 1.516404 | 0.014005 |
| ENSMUSG00000022742 | 0 | 0 | 1.515573 | 0.025472 |
| ENSMUSG00000002147 | 0 | 0 | 1.514538 | 0.139877 |
| ENSMUSG00000097789 | 0 | 0 | 1.514487 | 0.033237 |
| ENSMUSG00000049439 | 0 | 0 | 1.512859 | 0.044294 |
| ENSMUSG00000021838 | 0 | 0 | 1.512673 | 0.1404 |
| ENSMUSG00000037049 | 0 | 0 | 1.511766 | 0.009168 |
| ENSMUSG00000028646 | 0 | 0 | 1.511324 | 0.083895 |
| ENSMUSG00000034271 | 0 | 0 | 1.508864 | 0.139796 |
| ENSMUSG00000024640 | 0 | 0 | 1.50776 | 0.195682 |
| ENSMUSG00000051341 | 0 | 0 | 1.507551 | 0.122722 |
| ENSMUSG00000001098 | 0 | 0 | 1.506916 | 0.084568 |
| ENSMUSG00000070392 | 0 | 0 | 1.504558 | 0.241049 |
| ENSMUSG00000029553 | 0 | 0 | 1.503415 | 0.066117 |
| ENSMUSG00000029093 | 0 | 0 | 1.50306 | 0.130924 |
| ENSMUSG00000000934 | 0 | 0 | 1.501642 | 0.006651 |
| ENSMUSG00000078877 | 0 | 0 | 1.500545 | 0.137499 |
| ENSMUSG00000033350 | 0 | 0 | -1.50053 | 0.043193 |
| ENSMUSG00000000787 | 0 | 0 | -1.50102 | 0.194446 |
| ENSMUSG00000078247 | 0 | 0 | -1.5022 | 0.016562 |
| ENSMUSG00000060216 | 0 | 0 | -1.5033 | 0.022607 |
| ENSMUSG00000071337 | 0 | 0 | -1.50342 | 0.006747 |
| ENSMUSG00000038623 | 0 | 0 | -1.50651 | 0.408362 |
| ENSMUSG00000026447 | 0 | 0 | -1.5069 | 0.113561 |
| ENSMUSG00000037031 | 0 | 0 | -1.50892 | 0.007942 |
| ENSMUSG00000019966 | 0 | 0 | -1.51171 | 0.107985 |
| ENSMUSG00000038175 | 0 | 0 | -1.51193 | 0.017698 |
| ENSMUSG00000030393 | 0 | 0 | -1.5122 | 0.111024 |
| ENSMUSG00000091931 | 0 | 0 | -1.51288 | 0.121848 |
| ENSMUSG00000026669 | 0 | 0 | -1.51479 | 0.094722 |
| ENSMUSG00000030203 | 0 | 0 | -1.51515 | 0.056594 |
| ENSMUSG00000057406 | 0 | 0 | -1.51544 | 0.040161 |
| ENSMUSG00000101355 | 0 | 0 | -1.51586 | 0.045494 |
| ENSMUSG00000030621 | 0 | 0 | -1.51604 | 0.347609 |
| ENSMUSG00000028289 | 0 | 0 | -1.5165 | 0.016301 |
| ENSMUSG00000052581 | 0 | 0 | -1.51912 | 0.286667 |
| ENSMUSG00000097048 | 0 | 0 | -1.51946 | 0.108965 |
| ENSMUSG00000022205 | 0 | 0 | -1.52033 | 0.06051 |
| ENSMUSG00000051065 | 0 | 0 | -1.52121 | 0.106366 |
| ENSMUSG00000025358 | 0 | 0 | -1.52281 | 0.041514 |
| ENSMUSG00000005899 | 0 | 0 | -1.52406 | 0.048741 |
| ENSMUSG00000000093 | 0 | 0 | -1.52448 | 0.030607 |
| ENSMUSG00000052336 | 0 | 0 | -1.52524 | 0.158667 |
| ENSMUSG00000022253 | 0 | 0 | -1.52757 | 0.007227 |
| ENSMUSG00000000028 | 0 | 0 | -1.52911 | 0.045018 |
| ENSMUSG00000021767 | 0 | 0 | -1.52915 | 0.04286 |
| ENSMUSG00000035828 | 0 | 0 | -1.53044 | 0.132977 |
| ENSMUSG00000021215 | 0 | 0 | -1.53126 | 0.009094 |
| ENSMUSG00000024754 | 0 | 0 | -1.53201 | 0.079613 |
| ENSMUSG00000004980 | 0 | 0 | -1.53231 | 0.036671 |
| ENSMUSG00000026646 | 0 | 0 | -1.53654 | 0.060982 |
| ENSMUSG00000072893 | 0 | 0 | -1.53838 | 0.112444 |
| ENSMUSG00000024713 | 0 | 0 | -1.53928 | 0.303579 |
| ENSMUSG00000048402 | 0 | 0 | -1.5412 | 0.165971 |
| ENSMUSG00000025008 | 0 | 0 | -1.54241 | 0.095389 |
| ENSMUSG00000015937 | 0 | 0 | -1.54344 | 0.038838 |
| ENSMUSG00000059898 | 0 | 0 | -1.54381 | 0.187939 |
| ENSMUSG00000032064 | 0 | 0 | -1.54384 | 0.114955 |
| ENSMUSG00000058773 | 0 | 0 | -1.54468 | 0.162598 |
| ENSMUSG00000062510 | 0 | 0 | -1.54555 | 0.222268 |
| ENSMUSG00000017550 | 0 | 0 | -1.54628 | 0.131831 |
| ENSMUSG00000021287 | 0 | 0 | -1.5466 | 0.284757 |
| ENSMUSG00000005610 | 0 | 0 | -1.5467 | 0 |
| ENSMUSG00000040084 | 0 | 0 | -1.54694 | 0.046144 |
| ENSMUSG00000074622 | 0 | 0 | -1.54722 | 0.33014 |
| ENSMUSG00000028341 | 0 | 0 | -1.54818 | 0.051553 |
| ENSMUSG00000022723 | 0 | 0 | -1.54972 | 0.004187 |
| ENSMUSG00000032397 | 0 | 0 | -1.55008 | 0.031624 |
| ENSMUSG00000038481 | 0 | 0 | -1.55037 | 0.054874 |
| ENSMUSG00000064267 | 0 | 0 | -1.5518 | 0.087844 |
| ENSMUSG00000025747 | 0 | 0 | -1.55381 | 0.161362 |
| ENSMUSG00000039697 | 0 | 0 | -1.55867 | 0.068213 |
| ENSMUSG00000024921 | 0 | 0 | -1.55935 | 0.186919 |
| ENSMUSG00000039713 | 0 | 0 | -1.56028 | 0.089455 |
| ENSMUSG00000038893 | 0 | 0 | -1.56187 | 0.044165 |
| ENSMUSG00000034681 | 0 | 0 | -1.56195 | 0.013516 |
| ENSMUSG00000030725 | 0 | 0 | -1.56196 | 0.215629 |
| ENSMUSG00000038644 | 0 | 0 | -1.56273 | 0.078865 |
| ENSMUSG00000067586 | 0 | 0 | -1.5651 | 0.281111 |
| ENSMUSG00000053965 | 0 | 0 | -1.56754 | 0.057208 |
| ENSMUSG00000030315 | 0 | 0 | -1.56818 | 0.100456 |
| ENSMUSG00000052551 | 0 | 0 | -1.56944 | 0.132773 |
| ENSMUSG00000020658 | 0 | 0 | -1.57128 | 0.060244 |
| ENSMUSG00000087497 | 0 | 0 | -1.5717 | 0.164983 |
| ENSMUSG00000070858 | 0 | 0 | -1.5725 | 0.142007 |
| ENSMUSG00000021127 | 0 | 0 | -1.57371 | 0.21643 |
| ENSMUSG00000056476 | 0 | 0 | -1.57623 | 0.102223 |
| ENSMUSG00000030357 | 0 | 0 | -1.57728 | 0.060007 |
| ENSMUSG00000028184 | 0 | 0 | -1.57852 | 0.035438 |
| ENSMUSG00000052504 | 0 | 0 | -1.57853 | 0.157487 |
| ENSMUSG00000074476 | 0 | 0 | -1.57897 | 0.079787 |
| ENSMUSG00000099481 | 0 | 0 | -1.57973 | 0.115706 |
| ENSMUSG00000099583 | 0 | 0 | -1.58065 | 0.160035 |
| ENSMUSG00000030849 | 0 | 0 | -1.58104 | 0.147363 |
| ENSMUSG00000033904 | 0 | 0 | -1.58193 | 0.186115 |
| ENSMUSG00000040746 | 0 | 0 | -1.58469 | 0.068787 |
| ENSMUSG00000042807 | 0 | 0 | -1.58513 | 0.007822 |
| ENSMUSG00000073295 | 0 | 0 | -1.58553 | 0.32481 |
| ENSMUSG00000034460 | 0 | 0 | -1.58731 | 0.071694 |
| ENSMUSG00000035407 | 0 | 0 | -1.58865 | 0.114699 |
| ENSMUSG00000026872 | 0 | 0 | -1.5892 | 0.148021 |
| ENSMUSG00000020069 | 0 | 0 | -1.59004 | 0.093983 |
| ENSMUSG00000094338 | 0 | 0 | -1.5904 | 0.13507 |
| ENSMUSG00000022762 | 0 | 0 | -1.59042 | 0.199296 |
| ENSMUSG00000048058 | 0 | 0 | -1.5937 | 0.043892 |
| ENSMUSG00000098188 | 0 | 0 | -1.5938 | 0.008162 |
| ENSMUSG00000022999 | 0 | 0 | -1.60184 | 0.097091 |
| ENSMUSG00000017724 | 0 | 0 | -1.60262 | 0.318644 |
| ENSMUSG00000002870 | 0 | 0 | -1.60558 | 0.05646 |
| ENSMUSG00000040699 | 0 | 0 | -1.60576 | 0.056319 |
| ENSMUSG00000032666 | 0 | 0 | -1.6061 | 0.241694 |
| ENSMUSG00000027641 | 0 | 0 | -1.61081 | 0.008222 |
| ENSMUSG00000019952 | 0 | 0 | -1.61115 | 0.027453 |
| ENSMUSG00000000031 | 0 | 0 | -1.61232 | 0 |
| ENSMUSG00000060981 | 0 | 0 | -1.61239 | 0.125426 |
| ENSMUSG00000019880 | 0 | 0 | -1.61669 | 0.083369 |
| ENSMUSG00000010803 | 0 | 0 | -1.61734 | 0.052513 |
| ENSMUSG00000015568 | 0 | 0 | -1.61736 | 0.166991 |
| ENSMUSG00000053604 | 0 | 0 | -1.62162 | 0.33221 |
| ENSMUSG00000036768 | 0 | 0 | -1.62523 | 0.005747 |
| ENSMUSG00000047003 | 0 | 0 | -1.6258 | 0.071557 |
| ENSMUSG00000005447 | 0 | 0 | -1.62684 | 0.034571 |
| ENSMUSG00000053552 | 0 | 0 | -1.62867 | 0.027748 |
| ENSMUSG00000024098 | 0 | 0 | -1.62905 | 0.241229 |
| ENSMUSG00000024472 | 0 | 0 | -1.6313 | 0.08885 |
| ENSMUSG00000069270 | 0 | 0 | -1.63392 | 0.14891 |
| ENSMUSG00000032113 | 0 | 0 | -1.63636 | 0.017435 |
| ENSMUSG00000026923 | 0 | 0 | -1.63661 | 0.153668 |
| ENSMUSG00000025049 | 0 | 0 | -1.63821 | 0.151772 |
| ENSMUSG00000015749 | 0 | 0 | -1.64014 | 0.071634 |
| ENSMUSG00000019944 | 0 | 0 | -1.64074 | 0.210702 |
| ENSMUSG00000037236 | 0 | 0 | -1.64178 | 0.093001 |
| ENSMUSG00000094777 | 0 | 0 | -1.64247 | 0.133492 |
| ENSMUSG00000029836 | 0 | 0 | -1.64424 | 0.027336 |
| ENSMUSG00000079470 | 0 | 0 | -1.64611 | 0.088828 |
| ENSMUSG00000021377 | 0 | 0 | -1.64757 | 0.048604 |
| ENSMUSG00000069300 | 0 | 0 | -1.65013 | 0.037172 |
| ENSMUSG00000033970 | 0 | 0 | -1.65028 | 0.090089 |
| ENSMUSG00000061353 | 0 | 0 | -1.651 | 0.139678 |
| ENSMUSG00000026434 | 0 | 0 | -1.65123 | 0.040615 |
| ENSMUSG00000069310 | 0 | 0 | -1.65427 | 0.076104 |
| ENSMUSG00000043614 | 0 | 0 | -1.65547 | 0.343818 |
| ENSMUSG00000031827 | 0 | 0 | -1.65559 | 0.041772 |
| ENSMUSG00000020441 | 0 | 0 | -1.65576 | 0.079029 |
| ENSMUSG00000018379 | 0 | 0 | -1.65724 | 0.011004 |
| ENSMUSG00000021485 | 0 | 0 | -1.6578 | 0.033131 |
| ENSMUSG00000002489 | 0 | 0 | -1.6584 | 0.069798 |
| ENSMUSG00000093565 | 0 | 0 | -1.66327 | 0.342988 |
| ENSMUSG00000029245 | 0 | 0 | -1.66795 | 0.034353 |
| ENSMUSG00000017499 | 0 | 0 | -1.66945 | 0.096738 |
| ENSMUSG00000071172 | 0 | 0 | -1.66962 | 0.004065 |
| ENSMUSG00000049225 | 0 | 0 | -1.67224 | 0.205848 |
| ENSMUSG00000074793 | 0 | 0 | -1.67429 | 0.000839 |
| ENSMUSG00000027478 | 0 | 0 | -1.67683 | 0.118112 |
| ENSMUSG00000028034 | 0 | 0 | -1.67737 | 0.001595 |
| ENSMUSG00000084883 | 0 | 0 | -1.67778 | 0.118522 |
| ENSMUSG00000059878 | 0 | 0 | -1.67825 | 0.033914 |
| ENSMUSG00000099587 | 0 | 0 | -1.68452 | 0.47043 |
| ENSMUSG00000054520 | 0 | 0 | -1.68473 | 0.067722 |
| ENSMUSG00000024030 | 0 | 0 | -1.68748 | 0.471175 |
| ENSMUSG00000005225 | 0 | 0 | -1.68778 | 0.02568 |
| ENSMUSG00000059474 | 0 | 0 | -1.69155 | 0.069588 |
| ENSMUSG00000020935 | 0 | 0 | -1.69793 | 0.078479 |
| ENSMUSG00000087177 | 0 | 0 | -1.69921 | 0.161127 |
| ENSMUSG00000054679 | 0 | 0 | -1.70096 | 0.168384 |
| ENSMUSG00000022858 | 0 | 0 | -1.7031 | 0.080967 |
| ENSMUSG00000028031 | 0 | 0 | -1.70392 | 0.097703 |
| ENSMUSG00000042462 | 0 | 0 | -1.70455 | 0.09207 |
| ENSMUSG00000033502 | 0 | 0 | -1.70527 | 0.206285 |
| ENSMUSG00000007850 | 0 | 0 | -1.70543 | 0.291275 |
| ENSMUSG00000027829 | 0 | 0 | -1.70669 | 0.159654 |
| ENSMUSG00000030946 | 0 | 0 | -1.70878 | 0.273999 |
| ENSMUSG00000036478 | 0 | 0 | -1.71701 | 0.135166 |
| ENSMUSG00000027620 | 0 | 0 | -1.71799 | 0.038192 |
| ENSMUSG00000028358 | 0 | 0 | -1.72043 | 0.105875 |
| ENSMUSG00000024097 | 0 | 0 | -1.721 | 0.010768 |
| ENSMUSG00000037465 | 0 | 0 | -1.72289 | 0.213547 |
| ENSMUSG00000030279 | 0 | 0 | -1.72376 | 0.033927 |
| ENSMUSG00000041598 | 0 | 0 | -1.72752 | 0.09597 |
| ENSMUSG00000021062 | 0 | 0 | -1.72796 | 0.285263 |
| ENSMUSG00000020601 | 0 | 0 | -1.72963 | 0.092872 |
| ENSMUSG00000024190 | 0 | 0 | -1.73016 | 0.27552 |
| ENSMUSG00000028278 | 0 | 0 | -1.73114 | 0.074568 |
| ENSMUSG00000001497 | 0 | 0 | -1.73147 | 0.150108 |
| ENSMUSG00000024975 | 0 | 0 | -1.73306 | 0.231797 |
| ENSMUSG00000069274 | 0 | 0 | -1.73432 | 0.206725 |
| ENSMUSG00000005483 | 0 | 0 | -1.73602 | 0.054489 |
| ENSMUSG00000069114 | 0 | 0 | -1.73638 | 0.156719 |
| ENSMUSG00000053199 | 0 | 0 | -1.73735 | 0.320044 |
| ENSMUSG00000007817 | 0 | 0 | -1.73877 | 0.091905 |
| ENSMUSG00000018102 | 0 | 0 | -1.73922 | 0.14422 |
| ENSMUSG00000019874 | 0 | 0 | -1.74033 | 0.148152 |
| ENSMUSG00000035566 | 0 | 0 | -1.74086 | 0.107964 |
| ENSMUSG00000001016 | 0 | 0 | -1.74101 | 0.049125 |
| ENSMUSG00000041235 | 0 | 0 | -1.74105 | 0.282171 |
| ENSMUSG00000026494 | 0 | 0 | -1.74252 | 0.466592 |
| ENSMUSG00000028933 | 0 | 0 | -1.74586 | 0.008598 |
| ENSMUSG00000043881 | 0 | 0 | -1.74708 | 0.155642 |
| ENSMUSG00000052613 | 0 | 0 | -1.74726 | 0.330324 |
| ENSMUSG00000068101 | 0 | 0 | -1.74785 | 0.054987 |
| ENSMUSG00000004099 | 0 | 0 | -1.74899 | 0.003488 |
| ENSMUSG00000096768 | 0 | 0 | -1.75306 | 0.001469 |
| ENSMUSG00000022010 | 0 | 0 | -1.75791 | 0.077911 |
| ENSMUSG00000044197 | 0 | 0 | -1.75852 | 0.090654 |
| ENSMUSG00000041757 | 0 | 0 | -1.76014 | 0.003623 |
| ENSMUSG00000020547 | 0 | 0 | -1.76218 | 0.060994 |
| ENSMUSG00000023018 | 0 | 0 | -1.76379 | 0.0615 |
| ENSMUSG00000028532 | 0 | 0 | -1.76448 | 0.044942 |
| ENSMUSG00000038305 | 0 | 0 | -1.76557 | 0.271571 |
| ENSMUSG00000024222 | 0 | 0 | -1.76673 | 0.198886 |
| ENSMUSG00000049313 | 0 | 0 | -1.76839 | 0.016527 |
| ENSMUSG00000027074 | 0 | 0 | -1.77096 | 0.056443 |
| ENSMUSG00000095217 | 0 | 0 | -1.77114 | 0.333273 |
| ENSMUSG00000028884 | 0 | 0 | -1.77202 | 0.016486 |
| ENSMUSG00000058385 | 0 | 0 | -1.7736 | 0.234187 |
| ENSMUSG00000023150 | 0 | 0 | -1.77487 | 0.00769 |
| ENSMUSG00000000861 | 0 | 0 | -1.77834 | 0.07363 |
| ENSMUSG00000028799 | 0 | 0 | -1.78147 | 0.06011 |
| ENSMUSG00000033910 | 0 | 0 | -1.78413 | 0.008367 |
| ENSMUSG00000020831 | 0 | 0 | -1.785 | 0.043492 |
| ENSMUSG00000068854 | 0 | 0 | -1.78863 | 0.212699 |
| ENSMUSG00000029438 | 0 | 0 | -1.78894 | 0.005275 |
| ENSMUSG00000071478 | 0 | 0 | -1.79045 | 0.165046 |
| ENSMUSG00000045871 | 0 | 0 | -1.79096 | 0.420835 |
| ENSMUSG00000006585 | 0 | 0 | -1.79203 | 0.037203 |
| ENSMUSG00000001802 | 0 | 0 | -1.79219 | 0.278414 |
| ENSMUSG00000028863 | 0 | 0 | -1.79259 | 0.035807 |
| ENSMUSG00000009575 | 0 | 0 | -1.7926 | 0.002651 |
| ENSMUSG00000052632 | 0 | 0 | -1.79446 | 0.010656 |
| ENSMUSG00000028820 | 0 | 0 | -1.79561 | 0.013159 |
| ENSMUSG00000006715 | 0 | 0 | -1.79915 | 0.002547 |
| ENSMUSG00000027306 | 0 | 0 | -1.8002 | 0.050184 |
| ENSMUSG00000051517 | 0 | 0 | -1.80123 | 0.017561 |
| ENSMUSG00000020661 | 0 | 0 | -1.80151 | 0.075802 |
| ENSMUSG00000034255 | 0 | 0 | -1.80368 | 0.204697 |
| ENSMUSG00000020866 | 0 | 0 | -1.80395 | 0.197353 |
| ENSMUSG00000019850 | 0 | 0 | -1.80457 | 0.336323 |
| ENSMUSG00000039985 | 0 | 0 | -1.80565 | 0.027783 |
| ENSMUSG00000060743 | 0 | 0 | -1.80634 | 0.114879 |
| ENSMUSG00000069268 | 0 | 0 | -1.80656 | 0.176605 |
| ENSMUSG00000027416 | 0 | 0 | -1.80987 | 0.034744 |
| ENSMUSG00000029675 | 0 | 0 | -1.81054 | 0.162091 |
| ENSMUSG00000038267 | 0 | 0 | -1.81167 | 0.033729 |
| ENSMUSG00000067430 | 0 | 0 | -1.81282 | 0.007059 |
| ENSMUSG00000045062 | 0 | 0 | -1.8152 | 0.363835 |
| ENSMUSG00000049001 | 0 | 0 | -1.81585 | 0.161262 |
| ENSMUSG00000039419 | 0 | 0 | -1.81829 | 0.099292 |
| ENSMUSG00000031112 | 0 | 0 | -1.82229 | 0.070327 |
| ENSMUSG00000069301 | 0 | 0 | -1.82278 | 0.165593 |
| ENSMUSG00000040260 | 0 | 0 | -1.8241 | 0.007754 |
| ENSMUSG00000063455 | 0 | 0 | -1.82725 | 0.068643 |
| ENSMUSG00000016559 | 0 | 0 | -1.82733 | 0.328153 |
| ENSMUSG00000054074 | 0 | 0 | -1.82787 | 0.067963 |
| ENSMUSG00000021763 | 0 | 0 | -1.82841 | 0.136963 |
| ENSMUSG00000029730 | 0 | 0 | -1.82844 | 0.109107 |
| ENSMUSG00000048814 | 0 | 0 | -1.82992 | 0.395108 |
| ENSMUSG00000023911 | 0 | 0 | -1.83067 | 0.069581 |
| ENSMUSG00000047146 | 0 | 0 | -1.83358 | 0.258336 |
| ENSMUSG00000033308 | 0 | 0 | -1.83532 | 0.018487 |
| ENSMUSG00000021175 | 0 | 0 | -1.83628 | 0.015882 |
| ENSMUSG00000023908 | 0 | 0 | -1.83685 | 0.134239 |
| ENSMUSG00000050379 | 0 | 0 | -1.83752 | 0.00217 |
| ENSMUSG00000024642 | 0 | 0 | -1.83925 | 0.158857 |
| ENSMUSG00000034957 | 0 | 0 | -1.84259 | 0.523806 |
| ENSMUSG00000098318 | 0 | 0 | -1.84714 | 0.375595 |
| ENSMUSG00000028906 | 0 | 0 | -1.84727 | 0.006151 |
| ENSMUSG00000029687 | 0 | 0 | -1.84737 | 0.037441 |
| ENSMUSG00000035275 | 0 | 0 | -1.84972 | 0.137968 |
| ENSMUSG00000030811 | 0 | 0 | -1.85024 | 0.00862 |
| ENSMUSG00000046794 | 0 | 0 | -1.85213 | 0.130386 |
| ENSMUSG00000032228 | 0 | 0 | -1.85254 | 0.040991 |
| ENSMUSG00000045193 | 0 | 0 | -1.85762 | 0.15675 |
| ENSMUSG00000020092 | 0 | 0 | -1.85793 | 0.237185 |
| ENSMUSG00000031351 | 0 | 0 | -1.85839 | 0.003723 |
| ENSMUSG00000040430 | 0 | 0 | -1.86428 | 0.143805 |
| ENSMUSG00000045071 | 0 | 0 | -1.86436 | 0.007307 |
| ENSMUSG00000061482 | 0 | 0 | -1.86483 | 0.183577 |
| ENSMUSG00000057098 | 0 | 0 | -1.86548 | 0.155361 |
| ENSMUSG00000029090 | 0 | 0 | -1.86557 | 0.017838 |
| ENSMUSG00000072949 | 0 | 0 | -1.86656 | 0.106194 |
| ENSMUSG00000032076 | 0 | 0 | -1.86818 | 0.159978 |
| ENSMUSG00000053897 | 0 | 0 | -1.86891 | 0.140912 |
| ENSMUSG00000069769 | 0 | 0 | -1.8695 | 0.023901 |
| ENSMUSG00000085396 | 0 | 0 | -1.87048 | 0.084669 |
| ENSMUSG00000026556 | 0 | 0 | -1.87113 | 0.031567 |
| ENSMUSG00000047786 | 0 | 0 | -1.87257 | 0.190723 |
| ENSMUSG00000033364 | 0 | 0 | -1.87329 | 0.082826 |
| ENSMUSG00000075707 | 0 | 0 | -1.87827 | 0.056028 |
| ENSMUSG00000019906 | 0 | 0 | -1.87852 | 0.097219 |
| ENSMUSG00000025892 | 0 | 0 | -1.88053 | 0.133385 |
| ENSMUSG00000025395 | 0 | 0 | -1.88078 | 0.049367 |
| ENSMUSG00000069302 | 0 | 0 | -1.88258 | 0.02055 |
| ENSMUSG00000046269 | 0 | 0 | -1.88305 | 0.063771 |
| ENSMUSG00000038552 | 0 | 0 | -1.88873 | 0.002016 |
| ENSMUSG00000041859 | 0 | 0 | -1.89259 | 0.007407 |
| ENSMUSG00000048251 | 0 | 0 | -1.89309 | 0.324207 |
| ENSMUSG00000014592 | 0 | 0 | -1.8931 | 0.270133 |
| ENSMUSG00000078773 | 0 | 0 | -1.89524 | 0.092519 |
| ENSMUSG00000034245 | 0 | 0 | -1.89703 | 0.169142 |
| ENSMUSG00000040841 | 0 | 0 | -1.89761 | 0.175321 |
| ENSMUSG00000020893 | 0 | 0 | -1.89837 | 0.504747 |
| ENSMUSG00000025272 | 0 | 0 | -1.90014 | 0.318411 |
| ENSMUSG00000026288 | 0 | 0 | -1.90111 | 0.117762 |
| ENSMUSG00000074682 | 0 | 0 | -1.90304 | 0.243747 |
| ENSMUSG00000014850 | 0 | 0 | -1.90457 | 0.008734 |
| ENSMUSG00000037211 | 0 | 0 | -1.906 | 0.082755 |
| ENSMUSG00000021608 | 0 | 0 | -1.90745 | 0.005724 |
| ENSMUSG00000024151 | 0 | 0 | -1.90955 | 0.038917 |
| ENSMUSG00000047749 | 0 | 0 | -1.91108 | 0.072914 |
| ENSMUSG00000069309 | 0 | 0 | -1.91333 | 0.219828 |
| ENSMUSG00000028076 | 0 | 0 | -1.91486 | 0.336629 |
| ENSMUSG00000037892 | 0 | 0 | -1.91528 | 0.150287 |
| ENSMUSG00000009876 | 0 | 0 | -1.91606 | 0.074668 |
| ENSMUSG00000024427 | 0 | 0 | -1.91901 | 0.105435 |
| ENSMUSG00000060639 | 0 | 0 | -1.91941 | 0.043483 |
| ENSMUSG00000024601 | 0 | 0 | -1.9198 | 0.133818 |
| ENSMUSG00000047793 | 0 | 0 | -1.92093 | 0.035171 |
| ENSMUSG00000025927 | 0 | 0 | -1.92146 | 0.470953 |
| ENSMUSG00000021895 | 0 | 0 | -1.92654 | 0.213775 |
| ENSMUSG00000044712 | 0 | 0 | -1.9322 | 0.186416 |
| ENSMUSG00000006720 | 0 | 0 | -1.93275 | 0.06469 |
| ENSMUSG00000020027 | 0 | 0 | -1.93294 | 0.00811 |
| ENSMUSG00000030319 | 0 | 0 | -1.93304 | 0.001593 |
| ENSMUSG00000084910 | 0 | 0 | -1.9336 | 0.043568 |
| ENSMUSG00000032024 | 0 | 0 | -1.93891 | 0.277466 |
| ENSMUSG00000031066 | 0 | 0 | -1.94523 | 0.174572 |
| ENSMUSG00000040785 | 0 | 0 | -1.94609 | 0.024973 |
| ENSMUSG00000071516 | 0 | 0 | -1.94785 | 0.238035 |
| ENSMUSG00000022309 | 0 | 0 | -1.95269 | 0.216946 |
| ENSMUSG00000026355 | 0 | 0 | -1.95453 | 0.062408 |
| ENSMUSG00000023391 | 0 | 0 | -1.95606 | 0.075048 |
| ENSMUSG00000026825 | 0 | 0 | -1.95777 | 0.333129 |
| ENSMUSG00000087365 | 0 | 0 | -1.96362 | 0.135664 |
| ENSMUSG00000039706 | 0 | 0 | -1.96381 | 0.2288 |
| ENSMUSG00000049539 | 0 | 0 | -1.96413 | 0.156852 |
| ENSMUSG00000022203 | 0 | 0 | -1.96703 | 0.098408 |
| ENSMUSG00000042606 | 0 | 0 | -1.96746 | 0.094776 |
| ENSMUSG00000038390 | 0 | 0 | -1.96802 | 0.112064 |
| ENSMUSG00000047181 | 0 | 0 | -1.97008 | 0.015946 |
| ENSMUSG00000092035 | 0 | 0 | -1.97195 | 0.21612 |
| ENSMUSG00000004642 | 0 | 0 | -1.97204 | 0.002995 |
| ENSMUSG00000030638 | 0 | 0 | -1.97408 | 0.052077 |
| ENSMUSG00000056947 | 0 | 0 | -1.98183 | 0.161917 |
| ENSMUSG00000024112 | 0 | 0 | -1.98208 | 0.051948 |
| ENSMUSG00000024791 | 0 | 0 | -1.9834 | 0.101205 |
| ENSMUSG00000018012 | 0 | 0 | -1.98355 | 0.153486 |
| ENSMUSG00000020902 | 0 | 0 | -1.98929 | 0.304161 |
| ENSMUSG00000042834 | 0 | 0 | -1.9893 | 0.284986 |
| ENSMUSG00000050666 | 0 | 0 | -1.99614 | 0.332127 |
| ENSMUSG00000097142 | 0 | 0 | -1.99765 | 0.051028 |
| ENSMUSG00000036019 | 0 | 0 | -2.00089 | 0.061776 |
| ENSMUSG00000022220 | 0 | 0 | -2.0019 | 0.033802 |
| ENSMUSG00000093656 | 0 | 0 | -2.00199 | 0.102935 |
| ENSMUSG00000036875 | 0 | 0 | -2.00644 | 0.108092 |
| ENSMUSG00000075029 | 0 | 0 | -2.0089 | 0.064434 |
| ENSMUSG00000019139 | 0 | 0 | -2.0113 | 0.078205 |
| ENSMUSG00000062727 | 0 | 0 | -2.01172 | 0.011967 |
| ENSMUSG00000064264 | 0 | 0 | -2.01213 | 0.163398 |
| ENSMUSG00000079242 | 0 | 0 | -2.0128 | 0.420908 |
| ENSMUSG00000040270 | 0 | 0 | -2.01386 | 0.082666 |
| ENSMUSG00000028484 | 0 | 0 | -2.01468 | 0.034604 |
| ENSMUSG00000012443 | 0 | 0 | -2.01774 | 0.011769 |
| ENSMUSG00000032249 | 0 | 0 | -2.01915 | 0.149097 |
| ENSMUSG00000067608 | 0 | 0 | -2.0193 | 0.03526 |
| ENSMUSG00000021182 | 0 | 0 | -2.02007 | 0.188268 |
| ENSMUSG00000031134 | 0 | 0 | -2.02106 | 0.27913 |
| ENSMUSG00000040310 | 0 | 0 | -2.02293 | 0.282454 |
| ENSMUSG00000069265 | 0 | 0 | -2.02753 | 0.181946 |
| ENSMUSG00000039568 | 0 | 0 | -2.03123 | 0.064373 |
| ENSMUSG00000091337 | 0 | 0 | -2.03149 | 0.024078 |
| ENSMUSG00000027796 | 0 | 0 | -2.03466 | 0.061611 |
| ENSMUSG00000031209 | 0 | 0 | -2.03491 | 0.077518 |
| ENSMUSG00000068617 | 0 | 0 | -2.03703 | 0.285267 |
| ENSMUSG00000028565 | 0 | 0 | -2.04263 | 0.066209 |
| ENSMUSG00000049252 | 0 | 0 | -2.04432 | 0.013389 |
| ENSMUSG00000020086 | 0 | 0 | -2.0445 | 0.107474 |
| ENSMUSG00000019960 | 0 | 0 | -2.0478 | 0.100374 |
| ENSMUSG00000050936 | 0 | 0 | -2.04781 | 0.023787 |
| ENSMUSG00000001228 | 0 | 0 | -2.04961 | 0.02446 |
| ENSMUSG00000037474 | 0 | 0 | -2.052 | 0.035546 |
| ENSMUSG00000059674 | 0 | 0 | -2.05204 | 0.367582 |
| ENSMUSG00000023087 | 0 | 0 | -2.05441 | 0.083903 |
| ENSMUSG00000080058 | 0 | 0 | -2.05718 | 0.201699 |
| ENSMUSG00000073888 | 0 | 0 | -2.05869 | 0.057734 |
| ENSMUSG00000026890 | 0 | 0 | -2.06081 | 0.132616 |
| ENSMUSG00000030075 | 0 | 0 | -2.06162 | 0.154753 |
| ENSMUSG00000074807 | 0 | 0 | -2.06193 | 0.196408 |
| ENSMUSG00000025026 | 0 | 0 | -2.06814 | 0.120328 |
| ENSMUSG00000032221 | 0 | 0 | -2.07015 | 0.237296 |
| ENSMUSG00000029348 | 0 | 0 | -2.07738 | 0.090177 |
| ENSMUSG00000032827 | 0 | 0 | -2.08185 | 0.135844 |
| ENSMUSG00000046591 | 0 | 0 | -2.08199 | 0.17568 |
| ENSMUSG00000086119 | 0 | 0 | -2.08211 | 0.110985 |
| ENSMUSG00000049823 | 0 | 0 | -2.08236 | 0.227289 |
| ENSMUSG00000047992 | 0 | 0 | -2.08481 | 0.194822 |
| ENSMUSG00000085936 | 0 | 0 | -2.08667 | 0.057149 |
| ENSMUSG00000016756 | 0 | 0 | -2.08696 | 0.216919 |
| ENSMUSG00000093738 | 0 | 0 | -2.08796 | 0.004759 |
| ENSMUSG00000064168 | 0 | 0 | -2.09237 | 0.167405 |
| ENSMUSG00000073910 | 0 | 0 | -2.09786 | 0.251888 |
| ENSMUSG00000021069 | 0 | 0 | -2.09951 | 0.066433 |
| ENSMUSG00000040209 | 0 | 0 | -2.10105 | 0.157557 |
| ENSMUSG00000031099 | 0 | 0 | -2.10307 | 0.048564 |
| ENSMUSG00000031714 | 0 | 0 | -2.10372 | 0.060174 |
| ENSMUSG00000029510 | 0 | 0 | -2.10877 | 0.265108 |
| ENSMUSG00000067276 | 0 | 0 | -2.10894 | 0.266746 |
| ENSMUSG00000092595 | 0 | 0 | -2.10934 | 0.342987 |
| ENSMUSG00000060093 | 0 | 0 | -2.11402 | 0.201712 |
| ENSMUSG00000001985 | 0 | 0 | -2.11622 | 0.259379 |
| ENSMUSG00000070720 | 0 | 0 | -2.11666 | 0.025786 |
| ENSMUSG00000021098 | 0 | 0 | -2.12684 | 0.48441 |
| ENSMUSG00000097347 | 0 | 0 | -2.13059 | 0.307387 |
| ENSMUSG00000032501 | 0 | 0 | -2.13247 | 0.103405 |
| ENSMUSG00000030726 | 0 | 0 | -2.14533 | 0.020807 |
| ENSMUSG00000071847 | 0 | 0 | -2.14566 | 0.134848 |
| ENSMUSG00000020212 | 0 | 0 | -2.14816 | 0.094139 |
| ENSMUSG00000008575 | 0 | 0 | -2.14968 | 0.089694 |
| ENSMUSG00000031442 | 0 | 0 | -2.15292 | 0.575146 |
| ENSMUSG00000056394 | 0 | 0 | -2.15432 | 0.156958 |
| ENSMUSG00000016494 | 0 | 0 | -2.15556 | 0.099193 |
| ENSMUSG00000037405 | 0 | 0 | -2.15802 | 0.315959 |
| ENSMUSG00000027342 | 0 | 0 | -2.15945 | 0.096142 |
| ENSMUSG00000046178 | 0 | 0 | -2.1622 | 0.185442 |
| ENSMUSG00000043384 | 0 | 0 | -2.1686 | 0.036637 |
| ENSMUSG00000036528 | 0 | 0 | -2.16921 | 0.085313 |
| ENSMUSG00000075028 | 0 | 0 | -2.1696 | 0.127908 |
| ENSMUSG00000027954 | 0 | 0 | -2.17891 | 0.010815 |
| ENSMUSG00000054115 | 0 | 0 | -2.17894 | 0.181891 |
| ENSMUSG00000062175 | 0 | 0 | -2.18325 | 0.217407 |
| ENSMUSG00000064373 | 0 | 0 | -2.18443 | 0.19992 |
| ENSMUSG00000026034 | 0 | 0 | -2.18917 | 0.103694 |
| ENSMUSG00000025478 | 0 | 0 | -2.18953 | 0.183539 |
| ENSMUSG00000055022 | 0 | 0 | -2.18971 | 0.030776 |
| ENSMUSG00000040128 | 0 | 0 | -2.19652 | 0.304096 |
| ENSMUSG00000038518 | 0 | 0 | -2.19713 | 0.065916 |
| ENSMUSG00000046434 | 0 | 0 | -2.19737 | 0.036182 |
| ENSMUSG00000036356 | 0 | 0 | -2.19794 | 0.372437 |
| ENSMUSG00000020607 | 0 | 0 | -2.19925 | 0.580101 |
| ENSMUSG00000021469 | 0 | 0 | -2.20177 | 0.545233 |
| ENSMUSG00000038517 | 0 | 0 | -2.203 | 0.047101 |
| ENSMUSG00000042581 | 0 | 0 | -2.20543 | 0.435543 |
| ENSMUSG00000048960 | 0 | 0 | -2.2064 | 0.200958 |
| ENSMUSG00000066551 | 0 | 0 | -2.20862 | 0.027035 |
| ENSMUSG00000030592 | 0 | 0 | -2.21561 | 0.054654 |
| ENSMUSG00000043635 | 0 | 0 | -2.21761 | 0.048586 |
| ENSMUSG00000005410 | 0 | 0 | -2.21801 | 0.043426 |
| ENSMUSG00000002985 | 0 | 0 | -2.22077 | 0.42624 |
| ENSMUSG00000044258 | 0 | 0 | -2.22497 | 0.250312 |
| ENSMUSG00000063021 | 0 | 0 | -2.22857 | 0.142714 |
| ENSMUSG00000041911 | 0 | 0 | -2.22937 | 0.127209 |
| ENSMUSG00000040204 | 0 | 0 | -2.23011 | 0.007505 |
| ENSMUSG00000070822 | 0 | 0 | -2.23123 | 0.109107 |
| ENSMUSG00000063632 | 0 | 0 | -2.23184 | 0.121134 |
| ENSMUSG00000023913 | 0 | 0 | -2.23358 | 0.307631 |
| ENSMUSG00000064141 | 0 | 0 | -2.23423 | 0.274581 |
| ENSMUSG00000028654 | 0 | 0 | -2.23897 | 0.085713 |
| ENSMUSG00000029591 | 0 | 0 | -2.2444 | 0.039418 |
| ENSMUSG00000001525 | 0 | 0 | -2.24727 | 0 |
| ENSMUSG00000022885 | 0 | 0 | -2.25039 | 0.123534 |
| ENSMUSG00000060572 | 0 | 0 | -2.25138 | 0.178043 |
| ENSMUSG00000024232 | 0 | 0 | -2.25155 | 0.073258 |
| ENSMUSG00000100210 | 0 | 0 | -2.25291 | 0.156514 |
| ENSMUSG00000048997 | 0 | 0 | -2.25479 | 0.285783 |
| ENSMUSG00000029288 | 0 | 0 | -2.25979 | 0.485509 |
| ENSMUSG00000026676 | 0 | 0 | -2.26304 | 0.391554 |
| ENSMUSG00000020914 | 0 | 0 | -2.26812 | 0.066193 |
| ENSMUSG00000039994 | 0 | 0 | -2.26871 | 0.022551 |
| ENSMUSG00000042116 | 0 | 0 | -2.26899 | 0.286811 |
| ENSMUSG00000071369 | 0 | 0 | -2.26969 | 0.300283 |
| ENSMUSG00000064288 | 0 | 0 | -2.27528 | 0.069151 |
| ENSMUSG00000024134 | 0 | 0 | -2.28087 | 0.03406 |
| ENSMUSG00000024140 | 0 | 0 | -2.28242 | 0.604801 |
| ENSMUSG00000059005 | 0 | 0 | -2.28476 | 0.070159 |
| ENSMUSG00000041797 | 0 | 0 | -2.2862 | 0.190978 |
| ENSMUSG00000040896 | 0 | 0 | -2.28652 | 0.416027 |
| ENSMUSG00000028545 | 0 | 0 | -2.28703 | 0.173741 |
| ENSMUSG00000031994 | 0 | 0 | -2.2886 | 0.188194 |
| ENSMUSG00000031822 | 0 | 0 | -2.28964 | 0.206332 |
| ENSMUSG00000020524 | 0 | 0 | -2.29036 | 0.079008 |
| ENSMUSG00000024042 | 0 | 0 | -2.29109 | 0.400601 |
| ENSMUSG00000019961 | 0 | 0 | -2.29376 | 0.066199 |
| ENSMUSG00000022422 | 0 | 0 | -2.29468 | 0.038744 |
| ENSMUSG00000032624 | 0 | 0 | -2.30983 | 0.066737 |
| ENSMUSG00000039153 | 0 | 0 | -2.31225 | 0.025681 |
| ENSMUSG00000025529 | 0 | 0 | -2.31492 | 0.041341 |
| ENSMUSG00000041112 | 0 | 0 | -2.32656 | 0.163455 |
| ENSMUSG00000069273 | 0 | 0 | -2.33105 | 0.223868 |
| ENSMUSG00000063445 | 0 | 0 | -2.33355 | 0.116402 |
| ENSMUSG00000022673 | 0 | 0 | -2.33488 | 0.101298 |
| ENSMUSG00000032101 | 0 | 0 | -2.33738 | 0.013189 |
| ENSMUSG00000038331 | 0 | 0 | -2.34443 | 0.199059 |
| ENSMUSG00000051855 | 0 | 0 | -2.34993 | 0.512499 |
| ENSMUSG00000053113 | 0 | 0 | -2.35507 | 0.357437 |
| ENSMUSG00000026955 | 0 | 0 | -2.35515 | 0.113359 |
| ENSMUSG00000046982 | 0 | 0 | -2.35516 | 0.200885 |
| ENSMUSG00000029283 | 0 | 0 | -2.35799 | 0.033111 |
| ENSMUSG00000028524 | 0 | 0 | -2.35827 | 0.217696 |
| ENSMUSG00000052673 | 0 | 0 | -2.36701 | 0.175074 |
| ENSMUSG00000078851 | 0 | 0 | -2.36857 | 0.064884 |
| ENSMUSG00000021702 | 0 | 0 | -2.36872 | 0.214455 |
| ENSMUSG00000025154 | 0 | 0 | -2.36953 | 0.101059 |
| ENSMUSG00000029335 | 0 | 0 | -2.36963 | 0.127083 |
| ENSMUSG00000028832 | 0 | 0 | -2.37099 | 0.078583 |
| ENSMUSG00000028909 | 0 | 0 | -2.37391 | 0.278724 |
| ENSMUSG00000007805 | 0 | 0 | -2.37596 | 0.1228 |
| ENSMUSG00000096807 | 0 | 0 | -2.38199 | 0.083551 |
| ENSMUSG00000021676 | 0 | 0 | -2.38226 | 0.106172 |
| ENSMUSG00000070527 | 0 | 0 | -2.3825 | 0.260221 |
| ENSMUSG00000056145 | 0 | 0 | -2.38561 | 0.03996 |
| ENSMUSG00000098557 | 0 | 0 | -2.3859 | 0.085429 |
| ENSMUSG00000022519 | 0 | 0 | -2.38606 | 0.025682 |
| ENSMUSG00000042155 | 0 | 0 | -2.38852 | 0.001942 |
| ENSMUSG00000027454 | 0 | 0 | -2.39081 | 0.074345 |
| ENSMUSG00000044469 | 0 | 0 | -2.3917 | 0.10661 |
| ENSMUSG00000043004 | 0 | 0 | -2.39281 | 0.180467 |
| ENSMUSG00000074480 | 0 | 0 | -2.39498 | 0.003618 |
| ENSMUSG00000025658 | 0 | 0 | -2.39506 | 0.001079 |
| ENSMUSG00000061991 | 0 | 0 | -2.39582 | 0.257634 |
| ENSMUSG00000039157 | 0 | 0 | -2.39807 | 0.362533 |
| ENSMUSG00000029287 | 0 | 0 | -2.39958 | 0.036807 |
| ENSMUSG00000029231 | 0 | 0 | -2.40325 | 0.259051 |
| ENSMUSG00000051316 | 0 | 0 | -2.40959 | 0.398128 |
| ENSMUSG00000041126 | 0 | 0 | -2.40996 | 0.316719 |
| ENSMUSG00000030707 | 0 | 0 | -2.41007 | 0.063806 |
| ENSMUSG00000031383 | 0 | 0 | -2.41307 | 0.03655 |
| ENSMUSG00000097312 | 0 | 0 | -2.41418 | 0.38687 |
| ENSMUSG00000035936 | 0 | 0 | -2.4171 | 0.138421 |
| ENSMUSG00000020908 | 0 | 0 | -2.41831 | 0.698031 |
| ENSMUSG00000025577 | 0 | 0 | -2.42082 | 0.014369 |
| ENSMUSG00000034413 | 0 | 0 | -2.42218 | 0.06077 |
| ENSMUSG00000069272 | 0 | 0 | -2.42514 | 0.220361 |
| ENSMUSG00000022883 | 0 | 0 | -2.42532 | 0.238646 |
| ENSMUSG00000028036 | 0 | 0 | -2.43086 | 0.187228 |
| ENSMUSG00000049100 | 0 | 0 | -2.43152 | 0.167764 |
| ENSMUSG00000006369 | 0 | 0 | -2.45353 | 0.175403 |
| ENSMUSG00000097040 | 0 | 0 | -2.45627 | 0.303971 |
| ENSMUSG00000050240 | 0 | 0 | -2.46809 | 0.072215 |
| ENSMUSG00000020642 | 0 | 0 | -2.47971 | 0.297309 |
| ENSMUSG00000022371 | 0 | 0 | -2.48457 | 0.322888 |
| ENSMUSG00000043419 | 0 | 0 | -2.48467 | 0.010566 |
| ENSMUSG00000020388 | 0 | 0 | -2.48667 | 0.030739 |
| ENSMUSG00000047907 | 0 | 0 | -2.49262 | 0.230651 |
| ENSMUSG00000041592 | 0 | 0 | -2.49428 | 0.02456 |
| ENSMUSG00000052684 | 0 | 0 | -2.49617 | 0.359651 |
| ENSMUSG00000030092 | 0 | 0 | -2.50036 | 0.065231 |
| ENSMUSG00000067455 | 0 | 0 | -2.50147 | 0.122883 |
| ENSMUSG00000037754 | 0 | 0 | -2.50211 | 0.090844 |
| ENSMUSG00000038235 | 0 | 0 | -2.50499 | 0.219147 |
| ENSMUSG00000101698 | 0 | 0 | -2.51313 | 0.106828 |
| ENSMUSG00000097554 | 0 | 0 | -2.51786 | 0.064183 |
| ENSMUSG00000024014 | 0 | 0 | -2.52078 | 0.280896 |
| ENSMUSG00000034771 | 0 | 0 | -2.52519 | 0.122067 |
| ENSMUSG00000020183 | 0 | 0 | -2.53061 | 0.135802 |
| ENSMUSG00000003038 | 0 | 0 | -2.53556 | 0.06925 |
| ENSMUSG00000028931 | 0 | 0 | -2.53928 | 0.265311 |
| ENSMUSG00000032850 | 0 | 0 | -2.54888 | 0.144311 |
| ENSMUSG00000021097 | 0 | 0 | -2.5496 | 0.285207 |
| ENSMUSG00000024621 | 0 | 0 | -2.55024 | 0.052943 |
| ENSMUSG00000028197 | 0 | 0 | -2.55182 | 0.1134 |
| ENSMUSG00000025776 | 0 | 0 | -2.55413 | 0.02325 |
| ENSMUSG00000003824 | 0 | 0 | -2.55504 | 0.333045 |
| ENSMUSG00000000409 | 0 | 0 | -2.55611 | 0.405099 |
| ENSMUSG00000066705 | 0 | 0 | -2.55961 | 0.05919 |
| ENSMUSG00000019888 | 0 | 0 | -2.56195 | 0.124229 |
| ENSMUSG00000022865 | 0 | 0 | -2.56961 | 0.22054 |
| ENSMUSG00000032578 | 0 | 0 | -2.57193 | 0.082754 |
| ENSMUSG00000033152 | 0 | 0 | -2.59165 | 0.520293 |
| ENSMUSG00000066724 | 0 | 0 | -2.59241 | 0.091275 |
| ENSMUSG00000030600 | 0 | 0 | -2.59356 | 0.265785 |
| ENSMUSG00000046314 | 0 | 0 | -2.59438 | 0.21147 |
| ENSMUSG00000033981 | 0 | 0 | -2.59534 | 0.085873 |
| ENSMUSG00000055866 | 0 | 0 | -2.59831 | 0.103238 |
| ENSMUSG00000061143 | 0 | 0 | -2.60208 | 0.05549 |
| ENSMUSG00000005718 | 0 | 0 | -2.60403 | 0.059625 |
| ENSMUSG00000002289 | 0 | 0 | -2.6042 | 0.034738 |
| ENSMUSG00000016386 | 0 | 0 | -2.60949 | 0.126048 |
| ENSMUSG00000026826 | 0 | 0 | -2.61177 | 0.63024 |
| ENSMUSG00000040093 | 0 | 0 | -2.61652 | 0.442222 |
| ENSMUSG00000029371 | 0 | 0 | -2.63018 | 0.293129 |
| ENSMUSG00000040710 | 0 | 0 | -2.6343 | 0.110823 |
| ENSMUSG00000033083 | 0 | 0 | -2.64528 | 0.051833 |
| ENSMUSG00000000223 | 0 | 0 | -2.66603 | 0.40989 |
| ENSMUSG00000044927 | 0 | 0 | -2.66733 | 0.080274 |
| ENSMUSG00000074794 | 0 | 0 | -2.6734 | 0.500069 |
| ENSMUSG00000027748 | 0 | 0 | -2.67788 | 0.172029 |
| ENSMUSG00000022483 | 0 | 0 | -2.67881 | 0.122001 |
| ENSMUSG00000051043 | 0 | 0 | -2.6801 | 0.224348 |
| ENSMUSG00000040723 | 0 | 0 | -2.68428 | 0.077097 |
| ENSMUSG00000034648 | 0 | 0 | -2.68863 | 0.623506 |
| ENSMUSG00000033965 | 0 | 0 | -2.68954 | 0.287479 |
| ENSMUSG00000022199 | 0 | 0 | -2.69745 | 0.243545 |
| ENSMUSG00000027221 | 0 | 0 | -2.70283 | 0.251269 |
| ENSMUSG00000027217 | 0 | 0 | -2.70471 | 0.003111 |
| ENSMUSG00000032020 | 0 | 0 | -2.70979 | 0.112362 |
| ENSMUSG00000029309 | 0 | 0 | -2.71087 | 0.162695 |
| ENSMUSG00000031480 | 0 | 0 | -2.71153 | 0.062918 |
| ENSMUSG00000048834 | 0 | 0 | -2.71274 | 0.313927 |
| ENSMUSG00000024109 | 0 | 0 | -2.71311 | 0.0233 |
| ENSMUSG00000055612 | 0 | 0 | -2.71453 | 0.21569 |
| ENSMUSG00000035305 | 0 | 0 | -2.72386 | 0.187504 |
| ENSMUSG00000027358 | 0 | 0 | -2.72408 | 0.096143 |
| ENSMUSG00000036292 | 0 | 0 | -2.72769 | 0.268212 |
| ENSMUSG00000022015 | 0 | 0 | -2.73328 | 0.063278 |
| ENSMUSG00000039579 | 0 | 0 | -2.73463 | 0.058893 |
| ENSMUSG00000045333 | 0 | 0 | -2.7506 | 0.128989 |
| ENSMUSG00000025491 | 0 | 0 | -2.75836 | 0.340489 |
| ENSMUSG00000027820 | 0 | 0 | -2.76685 | 0.186868 |
| ENSMUSG00000010476 | 0 | 0 | -2.76967 | 0.318994 |
| ENSMUSG00000070713 | 0 | 0 | -2.77225 | 0.428061 |
| ENSMUSG00000003070 | 0 | 0 | -2.77326 | 0.325771 |
| ENSMUSG00000073530 | 0 | 0 | -2.78228 | 0.407867 |
| ENSMUSG00000020644 | 0 | 0 | -2.79374 | 0.096017 |
| ENSMUSG00000096010 | 0 | 0 | -2.79884 | 0.261852 |
| ENSMUSG00000002578 | 0 | 0 | -2.80325 | 0.086226 |
| ENSMUSG00000038777 | 0 | 0 | -2.80367 | 0.517157 |
| ENSMUSG00000026110 | 0 | 0 | -2.80944 | 0.225013 |
| ENSMUSG00000018899 | 0 | 0 | -2.80994 | 0.177339 |
| ENSMUSG00000069303 | 0 | 0 | -2.81568 | 0.287167 |
| ENSMUSG00000039328 | 0 | 0 | -2.81949 | 0.182015 |
| ENSMUSG00000034266 | 0 | 0 | -2.82096 | 0.052129 |
| ENSMUSG00000031661 | 0 | 0 | -2.82512 | 0.353844 |
| ENSMUSG00000064293 | 0 | 0 | -2.83105 | 0.065812 |
| ENSMUSG00000068614 | 0 | 0 | -2.83384 | 0.420563 |
| ENSMUSG00000028214 | 0 | 0 | -2.84443 | 0.063367 |
| ENSMUSG00000039976 | 0 | 0 | -2.84715 | 0.134265 |
| ENSMUSG00000019838 | 0 | 0 | -2.85213 | 0.045235 |
| ENSMUSG00000022231 | 0 | 0 | -2.85585 | 0.322606 |
| ENSMUSG00000063450 | 0 | 0 | -2.86358 | 0.266661 |
| ENSMUSG00000046402 | 0 | 0 | -2.86934 | 0.202305 |
| ENSMUSG00000037664 | 0 | 0 | -2.88063 | 0.178659 |
| ENSMUSG00000027111 | 0 | 0 | -2.88312 | 0.185569 |
| ENSMUSG00000028023 | 0 | 0 | -2.88647 | 0.525812 |
| ENSMUSG00000037108 | 0 | 0 | -2.88963 | 0.535782 |
| ENSMUSG00000032515 | 0 | 0 | -2.90327 | 0.459882 |
| ENSMUSG00000100750 | 0 | 0 | -2.90734 | 0.174122 |
| ENSMUSG00000060402 | 0 | 0 | -2.91574 | 0.318056 |
| ENSMUSG00000035228 | 0 | 0 | -2.91902 | 0.226679 |
| ENSMUSG00000099937 | 0 | 0 | -2.9217 | 0.678411 |
| ENSMUSG00000028581 | 0 | 0 | -2.9296 | 0.345929 |
| ENSMUSG00000051817 | 0 | 0 | -2.93073 | 0.291762 |
| ENSMUSG00000057329 | 0 | 0 | -2.94081 | 0.308864 |
| ENSMUSG00000029228 | 0 | 0 | -2.94636 | 0.063739 |
| ENSMUSG00000029673 | 0 | 0 | -2.95025 | 0.003504 |
| ENSMUSG00000042195 | 0 | 0 | -2.96005 | 0.394535 |
| ENSMUSG00000033032 | 0 | 0 | -2.97339 | 0.228427 |
| ENSMUSG00000057722 | 0 | 0 | -2.97734 | 0.21989 |
| ENSMUSG00000028693 | 0 | 0 | -2.98903 | 0.116347 |
| ENSMUSG00000042978 | 0 | 0 | -2.99236 | 0.20346 |
| ENSMUSG00000054717 | 0 | 0 | -3.00076 | 0.18698 |
| ENSMUSG00000054889 | 0 | 0 | -3.01401 | 0.54925 |
| ENSMUSG00000047246 | 0 | 0 | -3.01628 | 0.309318 |
| ENSMUSG00000037206 | 0 | 0 | -3.01714 | 0.278919 |
| ENSMUSG00000026712 | 0 | 0 | -3.02121 | 0.202194 |
| ENSMUSG00000032492 | 0 | 0 | -3.03284 | 0.06145 |
| ENSMUSG00000027765 | 0 | 0 | -3.0338 | 0.13722 |
| ENSMUSG00000068740 | 0 | 0 | -3.05813 | 0.032889 |
| ENSMUSG00000043456 | 0 | 0 | -3.07576 | 0.286552 |
| ENSMUSG00000038264 | 0 | 0 | -3.07963 | 0.079204 |
| ENSMUSG00000045589 | 0 | 0 | -3.08739 | 0.132038 |
| ENSMUSG00000039349 | 0 | 0 | -3.09126 | 0.36673 |
| ENSMUSG00000018909 | 0 | 0 | -3.10238 | 0.024461 |
| ENSMUSG00000059022 | 0 | 0 | -3.10437 | 0.124226 |
| ENSMUSG00000038065 | 0 | 0 | -3.1066 | 0.060127 |
| ENSMUSG00000074505 | 0 | 0 | -3.1156 | 0.184678 |
| ENSMUSG00000074577 | 0 | 0 | -3.11846 | 0.050604 |
| ENSMUSG00000044177 | 0 | 0 | -3.12674 | 0.093332 |
| ENSMUSG00000021108 | 0 | 0 | -3.12902 | 0.233636 |
| ENSMUSG00000021998 | 0 | 0 | -3.13842 | 0.068033 |
| ENSMUSG00000002980 | 0 | 0 | -3.15046 | 0.080585 |
| ENSMUSG00000029333 | 0 | 0 | -3.15751 | 0.2106 |
| ENSMUSG00000009246 | 0 | 0 | -3.16166 | 0.181342 |
| ENSMUSG00000024534 | 0 | 0 | -3.16558 | 0.063015 |
| ENSMUSG00000042604 | 0 | 0 | -3.18042 | 0.394478 |
| ENSMUSG00000070802 | 0 | 0 | -3.18469 | 0.323465 |
| ENSMUSG00000044461 | 0 | 0 | -3.18501 | 0.053432 |
| ENSMUSG00000069308 | 0 | 0 | -3.18576 | 0.115082 |
| ENSMUSG00000027200 | 0 | 0 | -3.19357 | 0.166994 |
| ENSMUSG00000024590 | 0 | 0 | -3.19824 | 0.131054 |
| ENSMUSG00000021294 | 0 | 0 | -3.20225 | 0.071284 |
| ENSMUSG00000030091 | 0 | 0 | -3.21782 | 0.037979 |
| ENSMUSG00000022449 | 0 | 0 | -3.22055 | 0.217707 |
| ENSMUSG00000039115 | 0 | 0 | -3.22215 | 0.055224 |
| ENSMUSG00000091050 | 0 | 0 | -3.2235 | 0.206371 |
| ENSMUSG00000004558 | 0 | 0 | -3.22928 | 0.635498 |
| ENSMUSG00000026090 | 0 | 0 | -3.24352 | 0.390118 |
| ENSMUSG00000057457 | 0 | 0 | -3.24374 | 0.264042 |
| ENSMUSG00000057182 | 0 | 0 | -3.24657 | 0.056082 |
| ENSMUSG00000022773 | 0 | 0 | -3.26965 | 0.467685 |
| ENSMUSG00000017491 | 0 | 0 | -3.27043 | 0.216411 |
| ENSMUSG00000027996 | 0 | 0 | -3.27321 | 0.10238 |
| ENSMUSG00000022876 | 0 | 0 | -3.28784 | 0.772921 |
| ENSMUSG00000061080 | 0 | 0 | -3.2938 | 0.552199 |
| ENSMUSG00000068522 | 0 | 0 | -3.30722 | 0.234515 |
| ENSMUSG00000043015 | 0 | 0 | -3.31038 | 0.05955 |
| ENSMUSG00000005220 | 0 | 0 | -3.3262 | 0.064993 |
| ENSMUSG00000090122 | 0 | 0 | -3.32734 | 0.693637 |
| ENSMUSG00000015217 | 0 | 0 | -3.34753 | 0.07857 |
| ENSMUSG00000052276 | 0 | 0 | -3.359 | 0.222282 |
| ENSMUSG00000025780 | 0 | 0 | -3.37527 | 0.274394 |
| ENSMUSG00000045930 | 0 | 0 | -3.38911 | 0.381752 |
| ENSMUSG00000027544 | 0 | 0 | -3.39922 | 0.790959 |
| ENSMUSG00000026473 | 0 | 0 | -3.41443 | 0.018208 |
| ENSMUSG00000078747 | 0 | 0 | -3.41572 | 0.250155 |
| ENSMUSG00000040867 | 0 | 0 | -3.41857 | 0.036526 |
| ENSMUSG00000026828 | 0 | 0 | -3.43246 | 0.443166 |
| ENSMUSG00000020653 | 0 | 0 | -3.44891 | 0.303964 |
| ENSMUSG00000023886 | 0 | 0 | -3.45116 | 0.366132 |
| ENSMUSG00000043008 | 0 | 0 | -3.45335 | 0.256596 |
| ENSMUSG00000068606 | 0 | 0 | -3.45595 | 0.507639 |
| ENSMUSG00000022658 | 0 | 0 | -3.4612 | 0.341814 |
| ENSMUSG00000019990 | 0 | 0 | -3.46201 | 0.541716 |
| ENSMUSG00000058145 | 0 | 0 | -3.4722 | 0.19527 |
| ENSMUSG00000040624 | 0 | 0 | -3.47233 | 0.303325 |
| ENSMUSG00000022456 | 0 | 0 | -3.47658 | 0.149401 |
| ENSMUSG00000087006 | 0 | 0 | -3.47733 | 0.179665 |
| ENSMUSG00000031616 | 0 | 0 | -3.47842 | 0.442553 |
| ENSMUSG00000052544 | 0 | 0 | -3.48483 | 0.295987 |
| ENSMUSG00000044468 | 0 | 0 | -3.48709 | 0.060244 |
| ENSMUSG00000059857 | 0 | 0 | -3.50115 | 0.137308 |
| ENSMUSG00000024247 | 0 | 0 | -3.54378 | 0.0275 |
| ENSMUSG00000026360 | 0 | 0 | -3.5518 | 0.515898 |
| ENSMUSG00000039621 | 0 | 0 | -3.56181 | 0.345535 |
| ENSMUSG00000072966 | 0 | 0 | -3.56182 | 0.051814 |
| ENSMUSG00000018334 | 0 | 0 | -3.56769 | 0.225683 |
| ENSMUSG00000020357 | 0 | 0 | -3.57623 | 0.699235 |
| ENSMUSG00000022330 | 0 | 0 | -3.57862 | 0.176301 |
| ENSMUSG00000010529 | 0 | 0 | -3.58003 | 0.002723 |
| ENSMUSG00000026344 | 0 | 0 | -3.58091 | 0.298772 |
| ENSMUSG00000063689 | 0 | 0 | -3.59073 | 0.17869 |
| ENSMUSG00000050751 | 0 | 0 | -3.59087 | 0.103847 |
| ENSMUSG00000022623 | 0 | 0 | -3.59321 | 0.382324 |
| ENSMUSG00000016763 | 0 | 0 | -3.61523 | 0.364847 |
| ENSMUSG00000019737 | 0 | 0 | -3.62295 | 0.388633 |
| ENSMUSG00000030123 | 0 | 0 | -3.62682 | 0.015021 |
| ENSMUSG00000027338 | 0 | 0 | -3.63293 | 0.058723 |
| ENSMUSG00000057123 | 0 | 0 | -3.64141 | 0.032371 |
| ENSMUSG00000087535 | 0 | 0 | -3.64616 | 0.036968 |
| ENSMUSG00000026840 | 0 | 0 | -3.65162 | 0.029728 |
| ENSMUSG00000022602 | 0 | 0 | -3.65235 | 0.041808 |
| ENSMUSG00000090063 | 0 | 0 | -3.67861 | 0.095118 |
| ENSMUSG00000031772 | 0 | 0 | -3.68352 | 0.029257 |
| ENSMUSG00000040181 | 0 | 0 | -3.72492 | 0.062341 |
| ENSMUSG00000038486 | 0 | 0 | -3.72944 | 0.045674 |
| ENSMUSG00000026921 | 0 | 0 | -3.73485 | 0.000568 |
| ENSMUSG00000001348 | 0 | 0 | -3.73515 | 0.856589 |
| ENSMUSG00000027239 | 0 | 0 | -3.74502 | 0.102868 |
| ENSMUSG00000037813 | 0 | 0 | -3.75104 | 0.289339 |
| ENSMUSG00000032179 | 0 | 0 | -3.76096 | 0.693041 |
| ENSMUSG00000018166 | 0 | 0 | -3.76315 | 0.221359 |
| ENSMUSG00000031790 | 0 | 0 | -3.77283 | 0.110511 |
| ENSMUSG00000020623 | 0 | 0 | -3.78858 | 0.225213 |
| ENSMUSG00000054871 | 0 | 0 | -3.78949 | 0.060738 |
| ENSMUSG00000024873 | 0 | 0 | -3.8067 | 0.216277 |
| ENSMUSG00000048120 | 0 | 0 | -3.80856 | 0.519083 |
| ENSMUSG00000034164 | 0 | 0 | -3.81227 | 0.469653 |
| ENSMUSG00000048572 | 0 | 0 | -3.82142 | 0.235941 |
| ENSMUSG00000047139 | 0 | 0 | -3.83774 | 0.258949 |
| ENSMUSG00000079662 | 0 | 0 | -3.84121 | 0.181897 |
| ENSMUSG00000032698 | 0 | 0 | -3.84906 | 0.259369 |
| ENSMUSG00000040653 | 0 | 0 | -3.85091 | 0.139564 |
| ENSMUSG00000019647 | 0 | 0 | -3.85972 | 0.152908 |
| ENSMUSG00000001227 | 0 | 0 | -3.86326 | 0.146613 |
| ENSMUSG00000050505 | 0 | 0 | -3.86845 | 0.202916 |
| ENSMUSG00000028360 | 0 | 0 | -3.88031 | 0.012018 |
| ENSMUSG00000051375 | 0 | 0 | -3.88351 | 0.218539 |
| ENSMUSG00000031028 | 0 | 0 | -3.889 | 0.419521 |
| ENSMUSG00000035835 | 0 | 0 | -3.89712 | 0.032433 |
| ENSMUSG00000038156 | 0 | 0 | -3.90118 | 0.062712 |
| ENSMUSG00000051041 | 0 | 0 | -3.92504 | 0.128424 |
| ENSMUSG00000006386 | 0 | 0 | -3.95333 | 0.376372 |
| ENSMUSG00000054072 | 0 | 0 | -3.98379 | 0.032106 |
| ENSMUSG00000039004 | 0 | 0 | -3.98882 | 0.081629 |
| ENSMUSG00000063415 | 0 | 0 | -4.00366 | 0.226643 |
| ENSMUSG00000032717 | 0 | 0 | -4.01135 | 0.191388 |
| ENSMUSG00000030249 | 0 | 0 | -4.01199 | 0.467217 |
| ENSMUSG00000038077 | 0 | 0 | -4.01624 | 0.176214 |
| ENSMUSG00000056313 | 0 | 0 | -4.02003 | 0.750423 |
| ENSMUSG00000079055 | 0 | 0 | -4.03389 | 0.108242 |
| ENSMUSG00000018983 | 0 | 0 | -4.03847 | 0.195069 |
| ENSMUSG00000096847 | 0 | 0 | -4.06259 | 0.731369 |
| ENSMUSG00000030905 | 0 | 0 | -4.0691 | 0.427612 |
| ENSMUSG00000058297 | 0 | 0 | -4.09283 | 0.392449 |
| ENSMUSG00000053414 | 0 | 0 | -4.0943 | 0.220173 |
| ENSMUSG00000055540 | 0 | 0 | -4.09808 | 1.523361 |
| ENSMUSG00000044647 | 0 | 0 | -4.09914 | 0.774293 |
| ENSMUSG00000040289 | 0 | 0 | -4.1057 | 0.040651 |
| ENSMUSG00000028871 | 0 | 0 | -4.12732 | 0.160184 |
| ENSMUSG00000060044 | 0 | 0 | -4.12781 | 0.082206 |
| ENSMUSG00000023232 | 0 | 0 | -4.13184 | 0.294059 |
| ENSMUSG00000005672 | 0 | 0 | -4.1425 | 1.014033 |
| ENSMUSG00000032554 | 0 | 0 | -4.14388 | 1.004908 |
| ENSMUSG00000097336 | 0 | 0 | -4.14828 | 0.470717 |
| ENSMUSG00000041992 | 0 | 0 | -4.15468 | 0.106225 |
| ENSMUSG00000084946 | 0 | 0 | -4.16759 | 0.352292 |
| ENSMUSG00000039109 | 0 | 0 | -4.17577 | 0.09531 |
| ENSMUSG00000049409 | 0 | 0 | -4.19916 | 0.492946 |
| ENSMUSG00000024008 | 0 | 0 | -4.20597 | 0.196973 |
| ENSMUSG00000004633 | 0 | 0 | -4.22564 | 0.541791 |
| ENSMUSG00000061535 | 0 | 0 | -4.23261 | 0.386527 |
| ENSMUSG00000027716 | 0 | 0 | -4.24316 | 0.182554 |
| ENSMUSG00000033453 | 0 | 0 | -4.24321 | 0.457167 |
| ENSMUSG00000028024 | 0 | 0 | -4.26473 | 0.451177 |
| ENSMUSG00000022489 | 0 | 0 | -4.29045 | 0.463392 |
| ENSMUSG00000038775 | 0 | 0 | -4.29934 | 0.057751 |
| ENSMUSG00000053522 | 0 | 0 | -4.30361 | 0.306904 |
| ENSMUSG00000094910 | 0 | 0 | -4.3104 | 0.094323 |
| ENSMUSG00000028339 | 0 | 0 | -4.32969 | 0.228058 |
| ENSMUSG00000019027 | 0 | 0 | -4.34475 | 0.167766 |
| ENSMUSG00000021943 | 0 | 0 | -4.3457 | 0.249758 |
| ENSMUSG00000059049 | 0 | 0 | -4.40795 | 0.529067 |
| ENSMUSG00000021466 | 0 | 0 | -4.42092 | 0.023462 |
| ENSMUSG00000027985 | 0 | 0 | -4.46046 | 0.135759 |
| ENSMUSG00000020577 | 0 | 0 | -4.46142 | 0.031425 |
| ENSMUSG00000028782 | 0 | 0 | -4.46567 | 0.375963 |
| ENSMUSG00000052384 | 0 | 0 | -4.46981 | 0.669346 |
| ENSMUSG00000015957 | 0 | 0 | -4.48537 | 0.225377 |
| ENSMUSG00000041828 | 0 | 0 | -4.51198 | 0.304635 |
| ENSMUSG00000026594 | 0 | 0 | -4.52134 | 0.1515 |
| ENSMUSG00000041731 | 0 | 0 | -4.52183 | 0.179888 |
| ENSMUSG00000021223 | 0 | 0 | -4.53766 | 0.368943 |
| ENSMUSG00000042286 | 0 | 0 | -4.55058 | 0.397309 |
| ENSMUSG00000042961 | 0 | 0 | -4.55443 | 0.192447 |
| ENSMUSG00000016028 | 0 | 0 | -4.55511 | 0.084168 |
| ENSMUSG00000027435 | 0 | 0 | -4.58522 | 0.168208 |
| ENSMUSG00000022055 | 0 | 0 | -4.59927 | 0.423028 |
| ENSMUSG00000005237 | 0 | 0 | -4.63558 | 0.132951 |
| ENSMUSG00000026638 | 0 | 0 | -4.6595 | 0.852434 |
| ENSMUSG00000041515 | 0 | 0 | -4.66025 | 0.134095 |
| ENSMUSG00000041482 | 0 | 0 | -4.67192 | 0.228702 |
| ENSMUSG00000057337 | 0 | 0 | -4.70055 | 0.284639 |
| ENSMUSG00000015396 | 0 | 0 | -4.73088 | 1.036939 |
| ENSMUSG00000042607 | 0 | 0 | -4.73207 | 0.356527 |
| ENSMUSG00000021256 | 0 | 0 | -4.73361 | 0.496153 |
| ENSMUSG00000050071 | 0 | 0 | -4.7478 | 0.36647 |
| ENSMUSG00000037995 | 0 | 0 | -4.74795 | 0.364904 |
| ENSMUSG00000031906 | 0 | 0 | -4.77424 | 0.043833 |
| ENSMUSG00000017978 | 0 | 0 | -4.77968 | 0.343929 |
| ENSMUSG00000001053 | 0 | 0 | -4.78942 | 0.20458 |
| ENSMUSG00000041119 | 0 | 0 | -4.79609 | 0.334447 |
| ENSMUSG00000011118 | 0 | 0 | -4.80152 | 0.384941 |
| ENSMUSG00000033544 | 0 | 0 | -4.81835 | 0.844881 |
| ENSMUSG00000067786 | 0 | 0 | -4.8366 | 0.540366 |
| ENSMUSG00000037492 | 0 | 0 | -4.87003 | 0.557811 |
| ENSMUSG00000049336 | 0 | 0 | -4.91504 | 0.082382 |
| ENSMUSG00000063564 | 0 | 0 | -4.92064 | 0.306394 |
| ENSMUSG00000032649 | 0 | 0 | -4.99589 | 0.628675 |
| ENSMUSG00000008153 | 0 | 0 | -5.00654 | 0.050174 |
| ENSMUSG00000037370 | 0 | 0 | -5.01686 | 0.101255 |
| ENSMUSG00000030653 | 0 | 0 | -5.03052 | 0.04859 |
| ENSMUSG00000022376 | 0 | 0 | -5.0679 | 0.237111 |
| ENSMUSG00000045394 | 0 | 0 | -5.07169 | 0.48039 |
| ENSMUSG00000020140 | 0 | 0 | -5.10173 | 0.049495 |
| ENSMUSG00000031517 | 0 | 0 | -5.12258 | 0.364112 |
| ENSMUSG00000041445 | 0 | 0 | -5.12613 | 0.141772 |
| ENSMUSG00000068874 | 0 | 0 | -5.15823 | 0.052236 |
| ENSMUSG00000047976 | 0 | 0 | -5.15891 | 0.63711 |
| ENSMUSG00000016179 | 0 | 0 | -5.1719 | 0.637476 |
| ENSMUSG00000058897 | 0 | 0 | -5.17569 | 0.046589 |
| ENSMUSG00000070304 | 0 | 0 | -5.18338 | 0.06439 |
| ENSMUSG00000001240 | 0 | 0 | -5.19696 | 0.33786 |
| ENSMUSG00000054793 | 0 | 0 | -5.20375 | 0.558498 |
| ENSMUSG00000026398 | 0 | 0 | -5.22612 | 0.498239 |
| ENSMUSG00000022122 | 0 | 0 | -5.22681 | 0.384801 |
| ENSMUSG00000054252 | 0 | 0 | -5.22851 | 0.008003 |
| ENSMUSG00000056492 | 0 | 0 | -5.2629 | 0.43311 |
| ENSMUSG00000022032 | 0 | 0 | -5.31934 | 0.718535 |
| ENSMUSG00000028640 | 0 | 0 | -5.3552 | 0.498584 |
| ENSMUSG00000025375 | 0 | 0 | -5.35976 | 0.392718 |
| ENSMUSG00000024451 | 0 | 0 | -5.39802 | 0.221756 |
| ENSMUSG00000017737 | 0 | 0 | -5.40227 | 0.312494 |
| ENSMUSG00000046618 | 0 | 0 | -5.42801 | 0.087366 |
| ENSMUSG00000020723 | 0 | 0 | -5.49842 | 0.25676 |
| ENSMUSG00000030669 | 0 | 0 | -5.5318 | 0.559315 |
| ENSMUSG00000026678 | 0 | 0 | -5.53482 | 0.016898 |
| ENSMUSG00000055254 | 0 | 0 | -5.54502 | 0.582639 |
| ENSMUSG00000069763 | 0 | 0 | -5.60579 | 0.225688 |
| ENSMUSG00000055629 | 0 | 0 | -5.61005 | 0.170331 |
| ENSMUSG00000027368 | 0 | 0 | -5.63094 | 0.157536 |
| ENSMUSG00000013584 | 0 | 0 | -5.66091 | 0.031366 |
| ENSMUSG00000017639 | 0 | 0 | -5.67679 | 0.608161 |
| ENSMUSG00000028766 | 0 | 0 | -5.73876 | 0.029874 |
| ENSMUSG00000029603 | 0 | 0 | -5.81767 | 0.410378 |
| ENSMUSG00000031837 | 0 | 0 | -5.86915 | 0.12023 |
| ENSMUSG00000037625 | 0 | 0 | -5.88022 | 0.597119 |
| ENSMUSG00000042750 | 0 | 0 | -5.89684 | 0.434787 |
| ENSMUSG00000062960 | 0 | 0 | -5.90376 | 0.599794 |
| ENSMUSG00000051159 | 0 | 0 | -5.92902 | 0.487688 |
| ENSMUSG00000044164 | 0 | 0 | -5.9314 | 0.159018 |
| ENSMUSG00000090698 | 0 | 0 | -5.97986 | 0.518653 |
| ENSMUSG00000036502 | 0 | 0 | -6.03804 | 0.229249 |
| ENSMUSG00000002799 | 0 | 0 | -6.07133 | 0.471128 |
| ENSMUSG00000081683 | 0 | 0 | -6.149 | 0.138018 |
| ENSMUSG00000036169 | 0 | 0 | -6.20706 | 0.286332 |
| ENSMUSG00000030376 | 0 | 0 | -6.21677 | 0.942521 |
| ENSMUSG00000022425 | 0 | 0 | -6.302 | 0.272408 |
| ENSMUSG00000026630 | 0 | 0 | -6.30216 | 0.411698 |
| ENSMUSG00000040856 | 0 | 0 | -6.41947 | 0.092315 |
| ENSMUSG00000024172 | 0 | 0 | -6.42915 | 0.017537 |
| ENSMUSG00000047085 | 0 | 0 | -6.47931 | 0.261462 |
| ENSMUSG00000031302 | 0 | 0 | -6.51678 | 0.428628 |
| ENSMUSG00000028626 | 0 | 0 | -6.52903 | 0.045435 |
| ENSMUSG00000000739 | 0 | 0 | -6.53572 | 0.36599 |
| ENSMUSG00000025608 | 0 | 0 | -6.54729 | 0.2059 |
| ENSMUSG00000024440 | 0 | 0 | -6.55705 | 0.175584 |
| ENSMUSG00000056895 | 0 | 0 | -6.57278 | 0.047623 |
| ENSMUSG00000045573 | 0 | 0 | -6.58433 | 0.264545 |
| ENSMUSG00000060780 | 0 | 0 | -6.60129 | 0.629995 |
| ENSMUSG00000001029 | 0 | 0 | -6.60338 | 0.48382 |
| ENSMUSG00000056222 | 0 | 0 | -6.6474 | 0.236221 |
| ENSMUSG00000000142 | 0 | 0 | -6.69761 | 0.009659 |
| ENSMUSG00000039037 | 0 | 0 | -6.70982 | 0.336237 |
| ENSMUSG00000060284 | 0 | 0 | -6.75129 | 0.130759 |
| ENSMUSG00000044562 | 0 | 0 | -6.75348 | 0.314487 |
| ENSMUSG00000025582 | 0 | 0 | -6.79556 | 0.314783 |
| ENSMUSG00000047867 | 0 | 0 | -6.82004 | 0.061622 |
| ENSMUSG00000054690 | 0 | 0 | -6.84501 | 0.246373 |
| ENSMUSG00000001946 | 0 | 0 | -6.85866 | 0.268519 |
| ENSMUSG00000027004 | 0 | 0 | -6.91008 | 0.097274 |
| ENSMUSG00000029086 | 0 | 0 | -6.92317 | 0.006175 |
| ENSMUSG00000028978 | 0 | 0 | -7.00112 | 0.026543 |
| ENSMUSG00000037169 | 0 | 0 | -7.06832 | 0.424067 |
| ENSMUSG00000018451 | 0 | 0 | -7.09637 | 0.212823 |
| ENSMUSG00000049103 | 0 | 0 | -7.19196 | 2.428675 |
| ENSMUSG00000000317 | 0 | 0 | -7.28476 | 0.152089 |
| ENSMUSG00000022577 | 0 | 0 | -7.29626 | 0.012362 |
| ENSMUSG00000058806 | 0 | 0 | -7.31378 | 0.606508 |
| ENSMUSG00000043496 | 0 | 0 | -7.49733 | 0.444615 |
| ENSMUSG00000022935 | 0 | 0 | -7.56519 | 2.158283 |
| ENSMUSG00000025422 | 0 | 0 | -7.6946 | 0.188866 |
| ENSMUSG00000060548 | 0 | 0 | -7.70483 | 0.141216 |
| ENSMUSG00000070498 | 0 | 0 | -7.79298 | 0.374176 |
| ENSMUSG00000020717 | 0 | 0 | -7.93116 | 0.945762 |
| ENSMUSG00000034127 | 0 | 0 | -8.06904 | 1.68518 |
| ENSMUSG00000004415 | 0 | 0 | -8.06907 | 0.089152 |
| ENSMUSG00000018169 | 0 | 0 | -8.14034 | 1.745079 |
| ENSMUSG00000024868 | 0 | 0 | -8.14264 | 2.09858 |
| ENSMUSG00000020218 | 0 | 0 | -8.2064 | 0.36037 |
| ENSMUSG00000078202 | 0 | 0 | -8.21322 | 1.732024 |
| ENSMUSG00000038188 | 0 | 0 | -8.27687 | 1.891612 |
| ENSMUSG00000000120 | 0 | 0 | -8.4092 | 0.226951 |
| ENSMUSG00000041708 | 0 | 0 | -8.51222 | 1.231518 |
| ENSMUSG00000034324 | 0 | 0 | -8.52989 | 0.068507 |
| ENSMUSG00000003934 | 0 | 0 | -8.54757 | 0.285077 |
| ENSMUSG00000004328 | 0 | 0 | -8.55691 | 1.538142 |
| ENSMUSG00000026587 | 0 | 0 | -8.65003 | 1.564002 |
| ENSMUSG00000027524 | 0 | 0 | -8.70662 | 1.592872 |
| ENSMUSG00000061527 | 0 | 0 | -8.83493 | 1.227447 |
| ENSMUSG00000047844 | 0 | 0 | -8.86266 | 2.268257 |
| ENSMUSG00000054641 | 0 | 0 | -9.0006 | 2.177809 |
| ENSMUSG00000030247 | 0 | 0 | -9.04404 | 1.205614 |
| ENSMUSG00000020810 | 0 | 0 | -9.12651 | 1.491222 |
| ENSMUSG00000015468 | 0 | 0 | -9.13536 | 1.817761 |
| ENSMUSG00000054435 | 0 | 0 | -9.31992 | 1.062533 |
| ENSMUSG00000034911 | 0 | 0 | -9.41132 | 1.272764 |
| ENSMUSG00000044338 | 0 | 0 | -9.48091 | 0.231349 |
| ENSMUSG00000073940 | 0 | 0 | -10.3075 | 0.437458 |
| ENSMUSG00000025270 | 0 | 0 | -10.4112 | 1.841556 |
| ENSMUSG00000029306 | 0 | 0 | -10.553 | 1.953002 |
| ENSMUSG00000069919 | 0 | 0 | -10.7619 | 0.741461 |
| ENSMUSG00000034845 | 0 | 0 | -10.9095 | 1.245479 |
| ENSMUSG00000034762 | 0 | 0 | -3.32823 | 1.591005 |
| ENSMUSG00000052305 | 0 | 0 | -11.3603 | 0.557951 |
| ENSMUSG00000055235 | 0 | 0 | -11.463 | 1.87048 |
| ENSMUSG00000050700 | 0 | 0 | -11.8844 | 1.156022 |
| ENSMUSG00000052187 | 0 | 0 | -12.6084 | 0.53201 |
