# Supplementary figures and images for: Insight into the maintenance of odontogenic potential in mouse dental mesenchymal cells based on transcriptomic analysis
Source: PeerJ. 2016 Feb 22;4:e1684. doi: 10.7717/peerj.1684 (PMC4768683; doi:10.7717/peerj.1684)

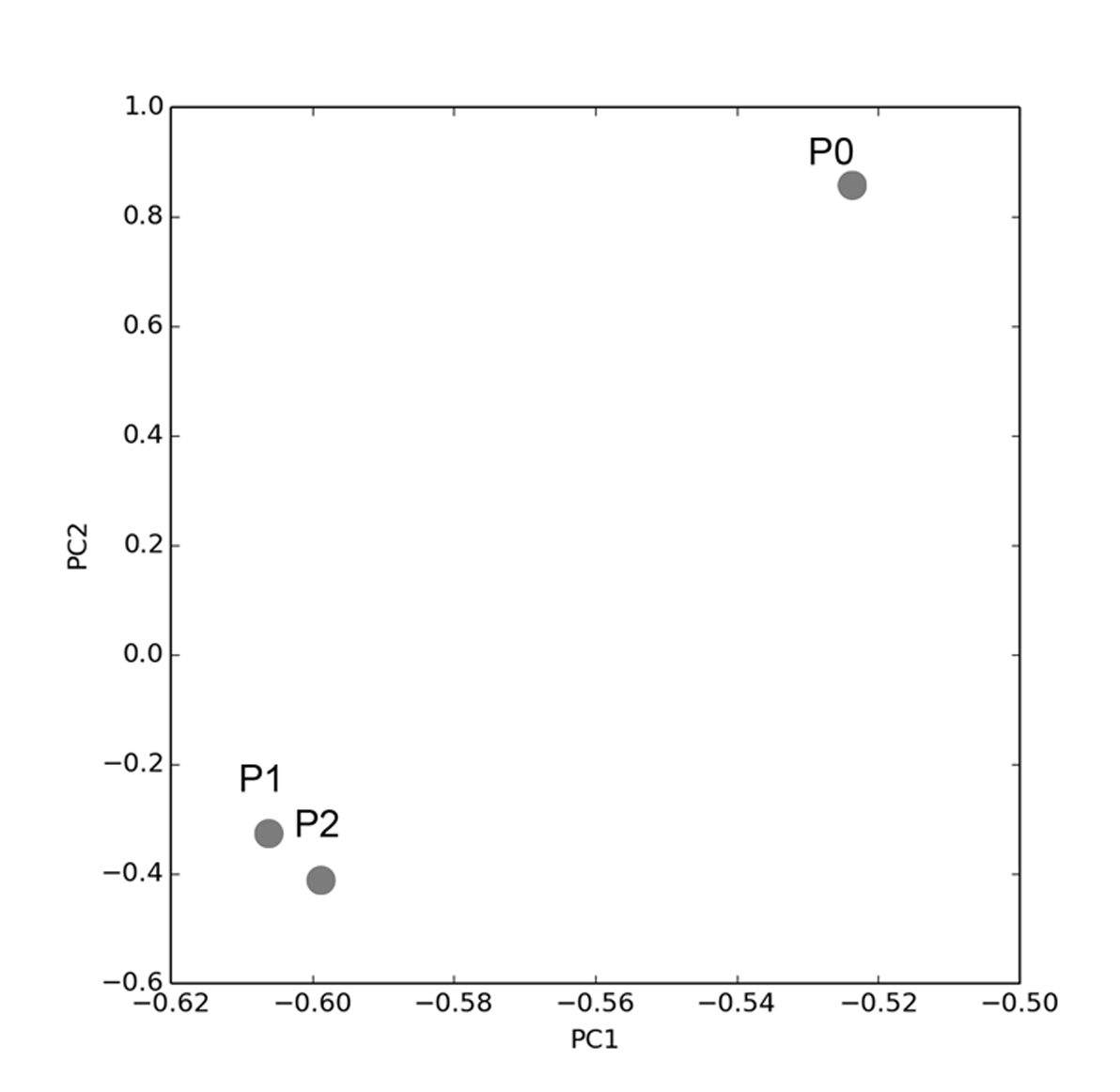

Supplement: Figure S1 — PCA revealed that the greatest variation in the transcriptional profile arose from differences in the P0 and P1 cells, while the P1 and P2 cells quite resemble each other. [file peerj-04-1684-s003.png]

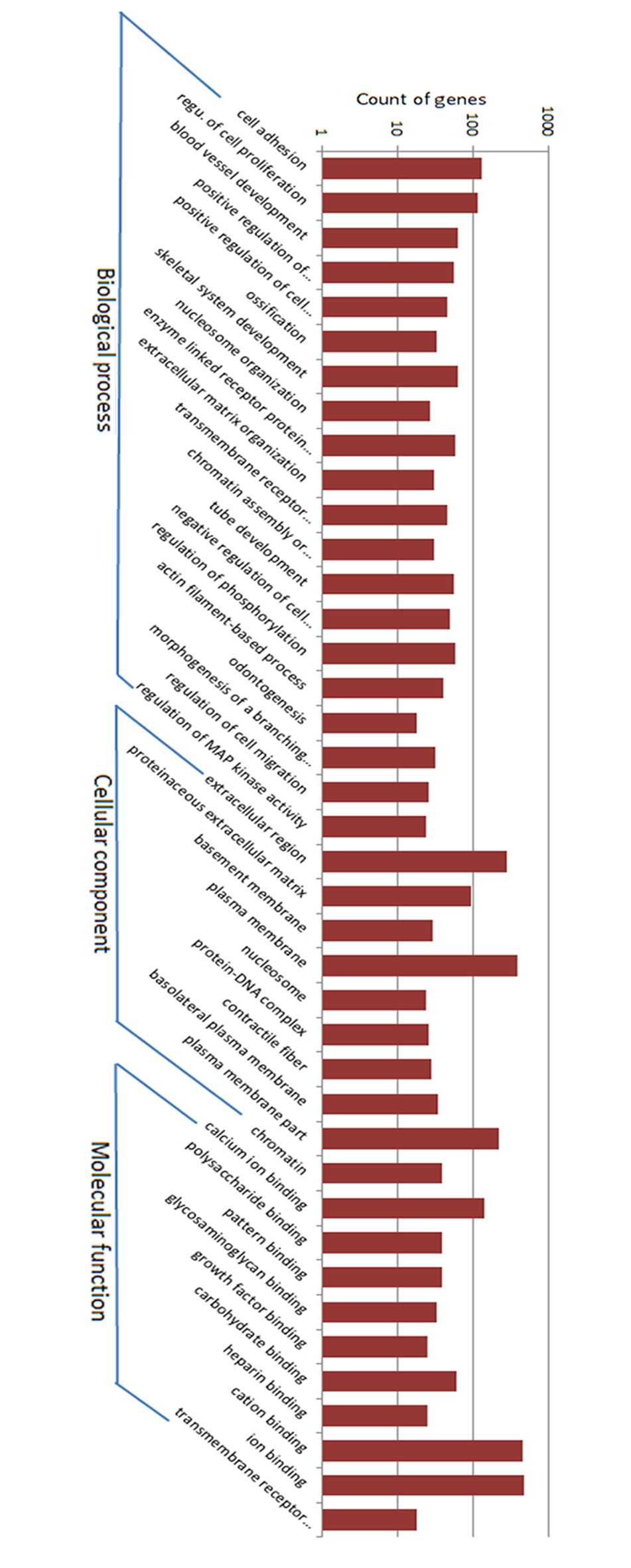

Supplement: Figure S2 — Significant GO biological processes, cellular components, and molecular function are listed (p < 1.60E-07). [file peerj-04-1684-s004.png]
